# Effect of Extra-Framework Anion Substitution on the Properties of a Chiral Crystalline Sponge

**DOI:** 10.1021/acs.cgd.3c00857

**Published:** 2023-09-28

**Authors:** Chenghua Deng, Bai-Qiao Song, Debobroto Sensharma, Mei-Yan Gao, Andrey A. Bezrukov, Varvara I. Nikolayenko, Matteo Lusi, Soumya Mukherjee, Michael J. Zaworotko

**Affiliations:** Bernal Institute, Department of Chemical Sciences, University of Limerick, Limerick V94 T9PX, Ireland

## Abstract

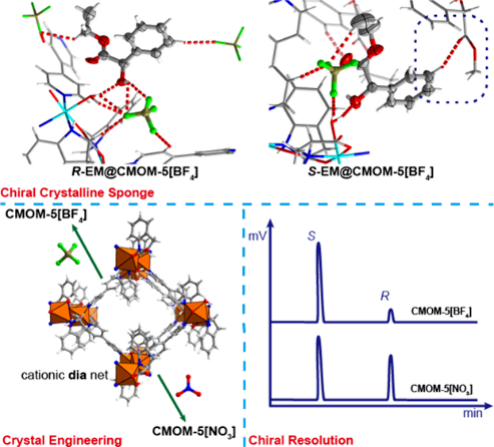

Chiral metal–organic materials, CMOMs, are of interest as they can offer selective binding sites for chiral guests. Such binding sites can enable CMOMs to serve as chiral crystalline sponges (CCSs) to determine molecular structure and/or purify enantiomers. We recently reported on the chiral recognition properties of a homochiral cationic diamondoid, dia, network {[Ni(*S*-IDEC)(bipy)(H_2_O)][NO_3_]}_*n*_ (*S*-IDEC = *S*-indoline-2-carboxylicate, bipy = 4,4′-bipyridine), **CMOM-5[NO**_**3**_**]**. The modularity of **CMOM-5[NO**_**3**_**]** means there are five feasible approaches to fine-tune structures and properties via substitution of one or more of the following components: metal cation (Ni^2+^); bridging ligand (*S*-IDEC); linker (bipy); extra-framework anion (NO_3_^–^); and terminal ligand (H_2_O). Herein, we report the effect of anion substitution on the CCS properties of **CMOM-5[NO**_**3**_**]** by preparing and characterizing {[Ni(*S*-IDEC)(bipy)(H_2_O)][BF_4_]}_*n*_, **CMOM-5[BF**_**4**_**]**. The chiral channels in **CMOM-5[BF**_**4**_**]** enabled it to function as a CCS for determination of the absolute crystal structures of both enantiomers of three chiral compounds: 1-phenyl-1-butanol (1P1B); methyl mandelate (MM); ethyl mandelate (EM). Chiral resolution experiments revealed **CMOM-5[BF**_**4**_**]** to be highly selective toward the *S*-isomers of MM and EM with enantiomeric excess, ee, values of 82.6 and 78.4%, respectively. The ee measured for *S*-EM surpasses the 64.3% exhibited by **[DyNaL(H**_**2**_**O)**_**4**_**] 6H**_**2**_**O** and far exceeds that of **CMOM-5[NO**_**3**_**]** (6.0%). Structural studies of the binding sites in **CMOM-5[BF**_**4**_**]** provide insight into their high enantioselectivity.

## Abstract 2

A new chiral metal−organic material provides a blueprint for designing families of chiral crystalline sponges that enable enantiomeric selectivity and determination of the crystal structures of liquid enantiomers.

## Introduction

The pharmaceutical and pesticide industries have developed products based upon enantiomerically pure active ingredients (AIs)^1−3^ because, whereas enantiomers have the same physicochemical properties, their biological properties can be distinctly different. For example, one enantiomer can be therapeutically effective and the other ineffective (e.g., verapamil) or toxic (e.g., thalidomide).^4,5^ Between 2018 and 2022, the US Food and Drug Administration approved 167 new drug products based upon small molecule AI(s); 101 of these are homochiral AIs and 9 are racemates.^6^ Access to homochiral compounds is therefore critical but challenging because enantiomers exhibit the same physicochemical properties.^7,8^ This renders their detection, separation, and synthesis challenging and/or costly.^7−10^ For example, whereas single-crystal X-ray diffraction (SCXRD) provides determination of absolute structures, it cannot be readily applied to ambient liquids or compounds only available in minute quantities, such as some natural products.^11,12^ In terms of separation, derivatives of organic oligomers (e.g., cyclodextrin) and polymers (e.g., cellulose) have been utilized as chiral materials for separation of racemates. Unfortunately, the strictly mobile phase requirement, low stability, and ambiguous structure can handicap commercial adoption.^13−15^ New chiral materials that can enable absolute structure determination and chiral separation and provide insights into binding mechanisms are therefore of topical interest.^16,17^

Metal–organic frameworks (MOFs), a subset of metal–organic materials (MOMs), are crystalline porous materials composed of organic linkers and metal-based building blocks, nodes, or clusters that form porous 2D or 3D networks.^18−21^ Thanks to their amenability to crystal engineering, when designed with the right pore size and pore chemistry, MOFs and related materials have afforded new benchmarks for gas/vapor storage and separations.^20−23^ In addition, the crystalline sponge method^24−26^ can harness the crystallinity of MOFs to enable adsorption and ordered packing of guests within their pores, in turn facilitating structure determination by SCXRD.^27−29^ The prototypal crystalline sponge, **[(ZnI**_**2**_**)**_**3**_**(tpt)**_**2**_**]**_***n***_ (tpt = tris(4-pyridyl)-1,3,5-triazine), exemplifies their potential utility, as in effect, it mimics enzymatic binding sites for a range of guest molecules.^27,30,31^

Chiral MOMs (CMOMs) can be generated using homochiral ligands^32−35^ and offer opportunities for chiral separation, detection, and catalysis. Derivatives of Schiff bases, binaphthols, and peptides have been studied in this context.^36−38^ Low-cost homochiral ligands such as camphorates have also been studied.^39^ Inspired by the latter approach, we have exploited the naturally occurring homochiral ligands mandelic acid and *S*-indoline-2-carboxylic acid (*S*-IDECH) to generate families of CMOMs through rod building blocks (RBBs).^6,40−43^ For mandelate CMOMs, four modular components can be readily substituted: metal cation (Co^2+^/Zn^2+^); bridging ligand (mandelate or their derivates); linker (4,4′-bipyridine, bipy); extra-framework counteranion (NO_3_^–^/OTf^–^/BF_4_^–^). The stoichiometry of metal cation/mandelate/bipy/counteranion is 1:1:1.5:1, and the RBBs formed by metal cations and mandelate anions are linked by three bipy linkers to afford cationic **bnn** topology networks.^40−43^ In the prototypal *S*-IDEC CMOM, {[Ni(*S*-IDEC)(bipy)(H_2_O)][NO_3_]}_*n*_, **CMOM-5[NO**_**3**_**]**, Ni^2+^ cations are bridged by *S*-IDEC anions to form RBBs cross-linked by bipy linkers to generate **dia** topology with NO_3_^–^ anions as extra-framework anions.^6^ The Ni^2+^ cations in the *S*-IDEC RBBs are linked by two bipy linkers, and the other coordination site is occupied by an aqua ligand. This means that **CMOM-5[NO**_**3**_**]** has five modular components in a 1:1:1:1:1 ratio. These robust CMOMs can serve as chiral crystalline sponges (CCSs) and as chiral stationary phases for gas chromatography (GC).^40−42^

Phenylalcohols and mandelate esters are well-known precursors for the synthesis of a wide range of pharmaceutical drug products.^44^ The enantiomers of 1-phenyl-1-butanol (1P1B), methyl mandelate (MM), and ethyl mandelate (EM) are used in the total synthesis of chiral APIs and several bioactive natural products, e.g., corticotropin-releasing factors, (−)-disorazole C1, and renin inhibitors.^45−49^ Our group has reported the structures of *R*-1P1B, *S*-1P1B, and *S*-MM based on **CMOM-3S** and **CMOM-5[NO**_**3**_**]**,^6,41^ but the crystal structures of the enantiomers of EM have not been reported in the CSD (Cambridge Structural Database) (Figures S3–S5, Table S1). In this work, we study how anion substitution affects the chiral recognition properties of **CMOM-5[NO**_**3**_**]** by substituting NO_3_^–^ with BF_4_^–^ to form **CMOM-5[BF**_**4**_**]**.

## Experimental Section

All reagents and solvents are commercially available and were used without further purification. Details of the experimental procedures are provided in the Supporting Information.

### Synthesis

A methanolic solution of Ni(BF_4_)_2_·6(H_2_O) and *S*-IDECH was mixed with a DMF (DMF = *N,N*-dimethylformamide) solution of bipy in a 15 mL glass vial. Blue crystals of {[Ni(*S*-IDEC)(bipy)(DMF)][BF_4_](DMF)}_*n*_ were obtained after the reaction mixture was heated at 85 °C for 1 day. Soaking the as-synthesized crystals in fresh batches of 10 mL of ethanol for 5 consecutive days resulted in transformation to {[Ni(*S*-IDEC)(bipy)(H_2_O)](BF_4_)(EtOH)_2_}_*n*_, **CMOM-5[BF**_**4**_**]·EtOH**. **CMOM-5[BF**_**4**_**]·EtOH** was activated by degassing on a SmartVacPrep using dynamic vacuum for 12 h at room temperature to afford {[Ni(*S*-IDEC)(bipy)(H_2_O)](BF_4_)}_*n*_, **CMOM-5[BF**_**4**_**]**.

### Characterization

SCXRD data were collected at 150 K using a Bruker D8 Quest diffractometer equipped with a Cu Kα IμS microfocus source (λ = 1.54178 Å) and a Photon II detector. Temperature was controlled by an Oxford Cryosystem with liquid nitrogen flow. Data were indexed by APEX4 (v2021.10–0); integrations were conducted by SAINT V8.40A in APEX4; absorption corrections were performed by SADABS in APEX4; space groups were determined by XPREP in APEX4. Crystal structures were solved by SHELXT through intrinsic phasing and refined by SHELXL through full-matrix least-squares on *F*^2^ in the Olex2–1.5 software package.^50−52^ Electron density corresponding to highly disordered guest molecules was addressed by PLATON SQUEEZE.^53^ Occupancies for chiral guest molecules were determined by considering MASK-calculated electrons and analyzing ^1^H nuclear magnetic resonance (NMR) data after sample digestion. NMR data were recorded using a JEOL ECX400 NMR spectrometer.^54,55^

### Chiral Resolution Studies

100 mg of **CMOM-5[BF**_**4**_**]·EtOH** crystals was soaked in 0.5 mL of ethanol containing 400 mg (or μL) of racemate. The screw cap was loosened to enable slow evaporation of ethanol over 5 days. Crystals were then filtered and washed with ethyl acetate (3 × 1 mL) and *n*-hexane (3 × 10 mL) to remove chiral molecules adhering to the surface of the crystals. Guest molecules in **CMOM-5[BF**_**4**_**]** were extracted by soaking the crystals in 10 mL of methanol for 3 days, after which the crystals were filtered and washed with methanol. The filtrates were combined, and the solvent was removed by using a rotary evaporator. The dried fractions were dissolved in 1 mL of isopropanol for ee analysis.

### Crystalline Sponge Studies

Crystals of **CMOM-5[BF**_**4**_**]·EtOH** were soaked in 0.5 mL of ethanol containing 40 mg (or μL) of an enantiomer of 1P1B, MM, or EM. The screw cap was loosened to enable slow evaporation of ethanol over 3 days; single crystals were isolated for SCXRD experiments.

## Results and Discussion

**Figure 1:**
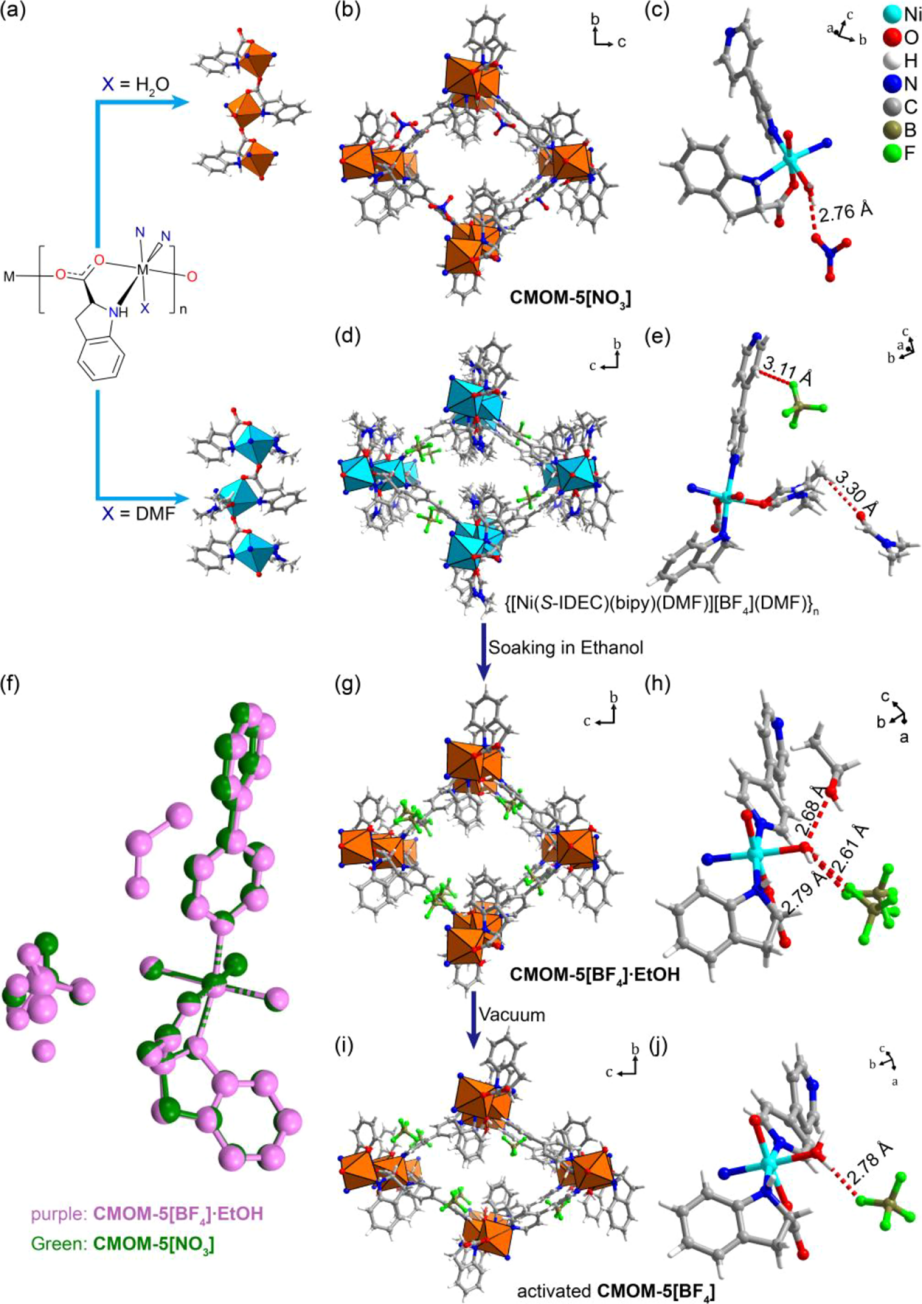
Crystal structures of **CMOM-5[NO**_**3**_**]** and **CMOM-5[BF**_**4**_**]**. (a) Schematic of the repeating unit in the *S*-IDEC RBB; X = terminal ligands (H_2_O or DMF). (b, d, g, and i) Structures of **CMOM-5[NO**_**3**_**]**, **CMOM-5[BF**_**4**_**]** DMF solvate, activated **CMOM-5[BF**_**4**_**]**, and **CMOM-5[BF**_**4**_**]·EtOH**, respectively, viewed along their channels; solvent molecules are omitted for clarity. (c, e, h, and j) Asymmetric units of **CMOM-5[NO**_**3**_**]**, **CMOM-5[BF**_**4**_**]** DMF solvate, **CMOM-5[BF**_**4**_**]·EtOH**, and activated **CMOM-5[BF**_**4**_**]**; donor-to-acceptor H-bonding interactions are labeled. (f) Overlay plot of the asymmetric units of **CMOM-5[NO**_**3**_**]** and **CMOM-5[BF**_**4**_**]·EtOH**; hydrogen atoms are omitted for clarity.

Blue single crystals of {[Ni(*S*-IDEC)(bipy)(DMF)](BF_4_)(DMF)}_*n*_ were obtained through solvothermal reaction of Ni(BF_4_)_2_, *S*-IDECH and bipy in DMF/methanol at 85 °C for 1 day (Figure S1a). SCXRD analysis revealed that the crystals had adopted the orthorhombic space group *P2*_*1*_*2*_*1*_*2*_*1*_ (Table S2). The crystal structure revealed that RBBs had formed via coordination of Ni^2+^ cations to *S*-IDEC bridging ligands in a manner similar to that for **CMOM-5[NO**_**3**_**]**. The terminally coordinated aqua ligand present in **CMOM-5[NO**_**3**_**]** was replaced by DMF (Figure 1a). RBBs were linked by bipy to form the expected **dia** net with BF_4_^–^ counteranions in channels (Figures 1d,e and S6). The remaining void space was occupied by DMF (Figure S10 and Table S10). TGA (thermogravimetric analysis) weight loss of 13.5 wt % at 145 °C (Figure S20) was consistent with the loss of DMF molecules.

Crystals of {[Ni(*S*-IDEC)(bipy)(DMF)][BF_4_](DMF)}_*n*_ were soaked in fresh batches of ethanol for five consecutive days. The as-synthesized crystals (Figure S1) were found to have split when examined under a microscope (Figure S2). SCXRD revealed them to have the composition {[Ni(*S*-IDEC)(bipy)(H_2_O)](BF_4_)(EtOH)_2_}_*n*_, **CMOM-5[BF**_**4**_**]·EtOH**, and that they had also crystallized in *P2*_*1*_*2*_*1*_*2*_*1*_ (Table S2) with aqua ligands instead of DMF ligands (Figures 1g and S6b). **CMOM-5[BF**_**4**_**]·EtOH** and **CMOM-5[NO**_**3**_**]** are isostructural and possess the same framework composition, only differing in their counteranions (Figure 1b,c,g,h). The counteranions in both **CMOM-5[BF**_**4**_**]·EtOH** and **CMOM-5[NO**_**3**_**]** were found to bind with the aqua ligand through the O–H···F (2.61–2.79 Å, Figures 1h and S11) and the O–H···O (2.76 Å, Figure 1c) hydrogen bonds, respectively (Figure 1f). The coordinated bipy ligand in **CMOM-5[BF**_**4**_**]·EtOH** has a narrower pyridine dihedral angle (30.52°) than that (37.72°) of **CMOM-5[NO**_**3**_**]** (Table S7). Around the C–C bond connecting the carboxylate and indoline moieties in *S*-IDEC ligands, the N–C–C–O torsion angle in **CMOM-5[BF**_**4**_**]·EtOH** (161.37°) is less than that in **CMOM-5[NO**_**3**_**]** (163.21°), whereas the C–C–C–O torsion angle (142.10°) is larger (137.72°). An ethanol molecule in the channel interacts with the BF4^–^ anion and aqua ligand through H-bonds (Figure S11 and Table S11). The ethanol molecule can be removed at 78 °C according to the TGA curve (Figure S21). That the structural changes also occur for the bulk sample is supported by PXRD diffractograms (Figures S23 and S24).

**CMOM-5[BF**_**4**_**]·EtOH** was found to undergo single-crystal-to-single-crystal (SCSC) transformation to activated **CMOM-5[BF**_**4**_**]** ({[Ni(*S*-IDEC)(bipy)(H_2_O)](BF_4_)}_*n*_) when exposed to vacuum at room temperature for 12 h. This transformation did not change the space group, but shrinkage of the structure (Figures 1i,j and S6b) occurred, as reflected in the unit cell volume (Table S3, Figures S7–S9, from 2928.1(4) to 2605.04(14) Å^3^). Removal of solvent molecules afforded 29.6% void space, as determined by PLATON SQUEEZE (Figure S12 and Table S12).^53^ Structural changes also occurred in the bulk sample, as validated by matching of calculated and experimental PXRD patterns (Figure S25). The TGA curve of activated **CMOM-5[BF**_**4**_**]** revealed a weight loss of 3.1 wt % at 78 °C (Figure S22), which we attribute to hydration.

**CMOM-5[BF**_**4**_**]** exhibited a two-step open-to-more-open Type F–II CO_2_ isotherm at 195 K (Figure S30).^56^ The first step occurred at *P*/*P*_0_ ∼ 0.13 with an uptake of 3.75 mmol g^–1^, while at *P*/*P*_0_ ∼ 1, the saturation uptake was 5.96 mmol g^–1^. For the fully open phase of **CMOM-5[BF**_**4**_**]**, the Langmuir surface area was determined to be 613 m^2^ g^–1^ (Figure S33). Four continuous cycles of 195 K CO_2_ sorption isotherms revealed that the phase change pressure and uptake are consistent over multiple cycles (Figure S31). The N_2_ adsorption isotherm for **CMOM-5[BF**_**4**_**]** registered a negligible uptake (Figure S30). The high-pressure CO_2_ adsorption isotherm at 298 K was also F–II isotherm type (Figure S32). The first step occurred at 8 bar with an uptake of 2.6 mmol g^–1^ and the second at 40 bar with an uptake of 5.1 mmol g^–1^.

**Figure 2:**
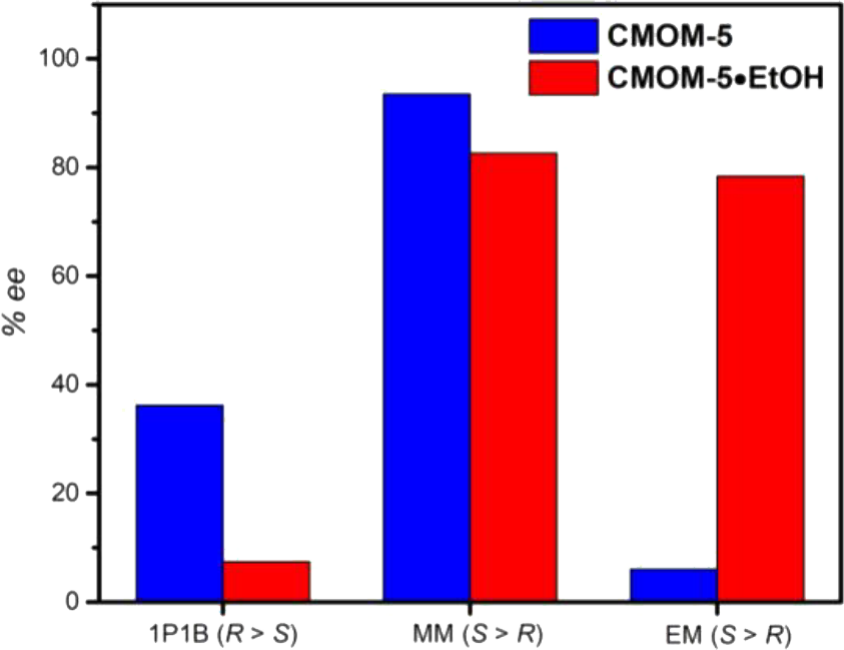
Chiral resolution performance of **CMOM-5[NO**_**3**_**]** and **CMOM-5[BF**_**4**_**]**.

Given its relatively high stability and flexibility, **CMOM-5[BF**_**4**_**]** was studied with respect to selective adsorption of enantiomers from racemates of 1P1B, MM, and EM. Single crystals of **CMOM-5[BF**_**4**_**]·EtOH** were soaked in ethanolic solutions of the racemates of 1P1B, MM, and EM for 5 days. Digestion with deuterium chloride and DMSO-*d*_6_ enabled us to use ^1^H NMR spectroscopy to determine the relative uptakes. The ratios of bipy/*S*-IDEC/1P1B, bipy/*S*-IDEC/MM, and bipy/*S*-IDEC/EM in the samples were found to be 1:1.01:1.16, 1:1:1.18, and 1:1.02:1.05, respectively (Figures S40–S42), meaning that each asymmetric unit accommodated one chiral guest molecule. The adsorbed guest molecules were extracted by methanol, and the ee values of the extracted chiral molecules were evaluated by high-performance liquid chromatography, HPLC, equipped with a chiral column (Figures S43–S54). For 1P1B, the *R*-isomer was preferentially sorbed with 7.4% ee, while for MM and EM, *S*- isomers were sorbed with 82.6% ee and 78.4% ee, respectively (Figure 2). When compared to **CMOM-5[NO**_**3**_**]**, although **CMOM-5[BF**_**4**_**]** exhibited reduced separation performances for 1P1B and MM, for EM, the enantioselectivity (78.4%) greatly exceeded that of **CMOM-5[NO**_**3**_**]** (6.0%, Figure S57). Indeed, the ee value for EM exceeds that (64.3%) of **[DyNaL(H**_**2**_**O)**_**4**_**] 6H**_**2**_**O** (H_4_L = 3,3′-di-*tert*-butyl-5,50-di(3,5-carboxyphenyl-1-yl)-6,6′-dimethylbiphenyl-2,2′-diol, Table S20).^57^

**CMOM-5[BF**_**4**_**]** was found to retain crystallinity even after the chiral resolution experiments, affording **CMOM-5[BF**_**4**_**]·MeOH** after extraction of the chiral guest using methanol (Table S3). Methanol molecules were observed to be disordered in channels with ca. 38.9% void space (Figure S13 and Table S13).^53^ PXRD patterns reveal that the bulk sample after chiral resolution experiments had transformed to **CMOM-5[BF**_**4**_**]·MeOH** (Figure S26) and that it could be reused for recycling and CCS studies. Crystals of **CMOM-5[BF**_**4**_**]·MeOH** were soaked in an ethanolic solution of racemic EM for the second and third cycles of chiral resolution experiments. The ee values were determined to be 79.9 and 79.4%, respectively, which revealed the feasibility of recycling **CMOM-5[BF**_**4**_**]** (Figures S55 and S56).

**Figure 3:**
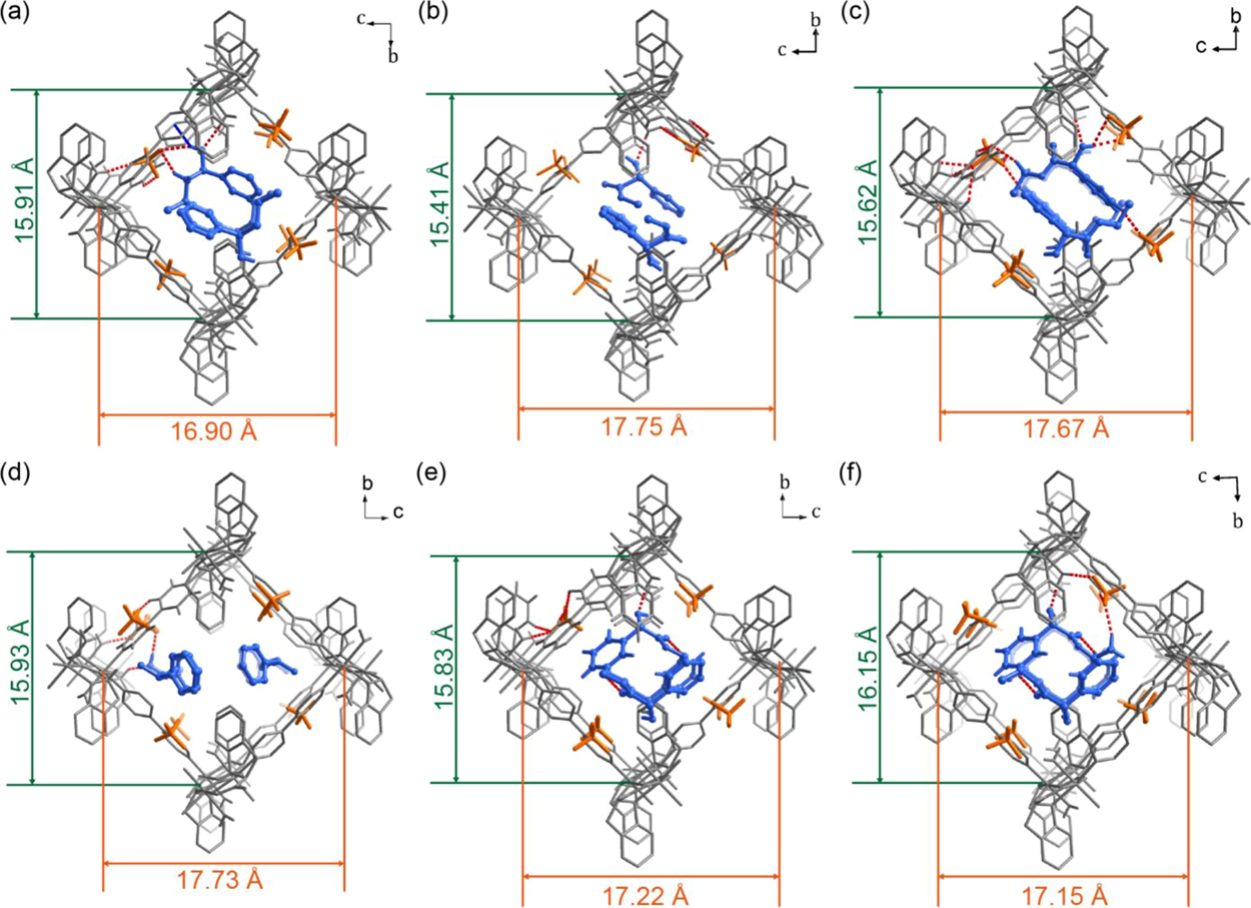
Binding sites of chiral guest molecules in **CMOM-5[BF**_**4**_**]**, as determined by SCXRD. (a–f) **CMOM-5[BF**_**4**_**]-***R***-1P1B**, **CMOM-5[BF**_**4**_**]-***R***-MM**, **CMOM-5[BF**_**4**_**]-***R***-EM**, **CMOM-5[BF**_**4**_**]-***S***-1P1B**, **CMOM-5[BF**_**4**_**]-***S***-MM**, and **CMOM-5[BF**_**4**_**]-***S***-EM**, respectively. Chiral guest molecules are colored blue; the host framework gray; and BF_4_^–^ anions orange; H-bonds are represented by red dashed lines. Hydrogen atoms in the C–H and N–H bonds are omitted for clarity.

In the guest-loaded structures, non-hydrogen atoms of the enantiomers were refined anisotropically (Figure 3). The unit cells of the phases accommodating chiral guests are similar to those of the as-synthesized solvate (Figures S7–S9, Tables S4–S6). However, there are differences after guest loading as seen from diagonal distances between Ni(II) cations across the channel (Figures 3 and S7). The linker ligand coordination geometries for bipy (Table S8) and *S*-IDEC (Table S9) indicate that the host framework had adapted itself to bind with the chiral guest molecules. PXRD patterns are consistent with the SCXRD data (Figures S27–S29). These subtle structural changes suggest that **CMOM-5[BF**_**4**_**]** is, in effect, a self-adaptive skeleton that adapts to guest molecules.

**Figure 4:**
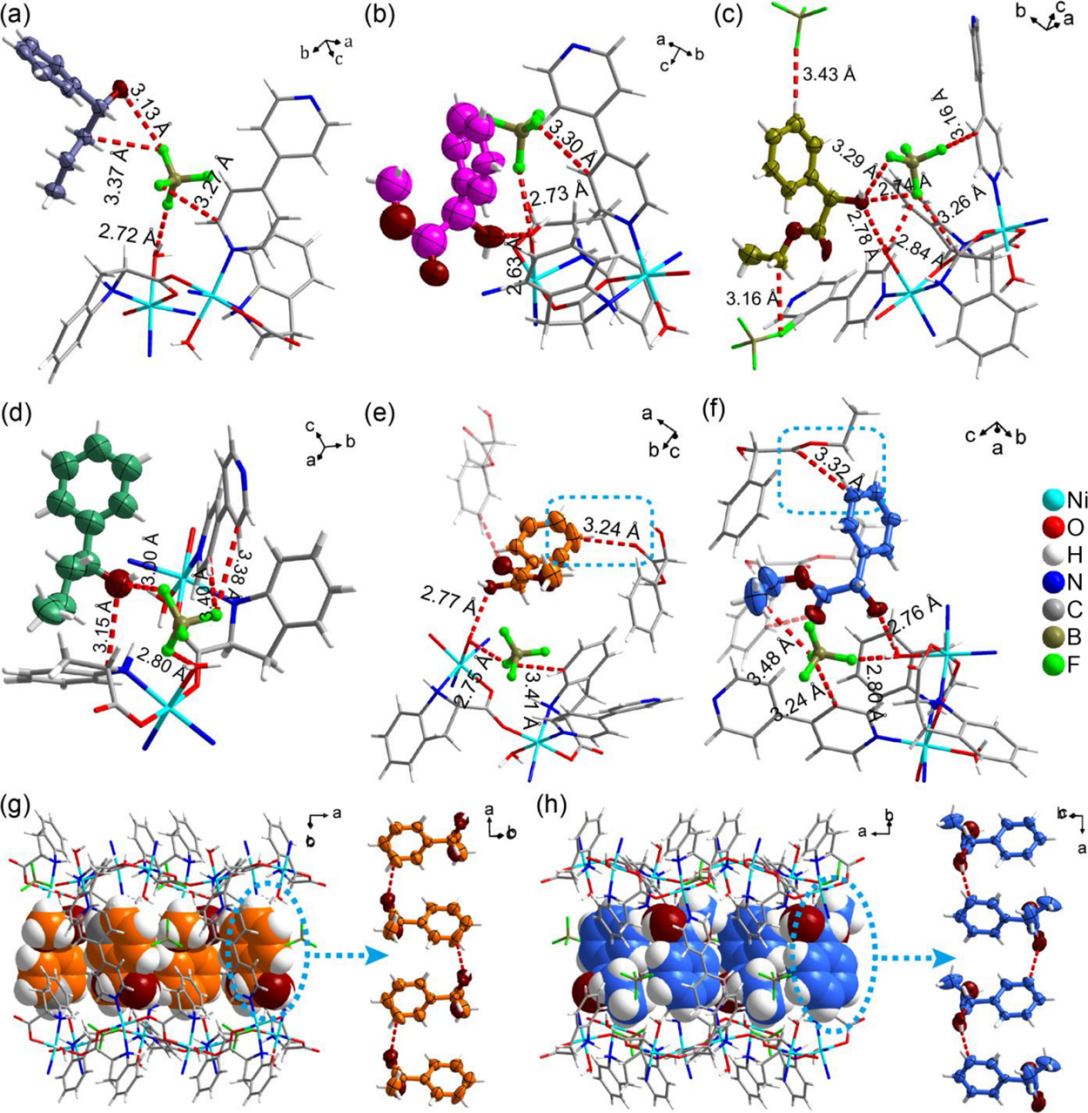
H-bonding interactions involving the chiral guest molecules in their binding sites. (a) to (f) correspond to *R*-1P1B, *R*-MM, *R*-EM, *S*-1P1B, *S*-MM, and *S*-EM. H-bonded chains of *S*-MM (g) and *S*-EM (h) lie in the channels of **CMOM-5[BF**_**4**_**]**. The non-hydrogen atoms of the chiral guest molecules are drawn in thermal ellipsoids at a 50% probability. The distances between donors and acceptors in the H-bonds marked by red dashed lines are given. Guest–guest H-bonds are highlighted by light blue dashed lines.

In the structures with chiral guest molecules, BF_4_^–^ anions form O–H···F (2.73∼2.84 Å) H-bonds with the aqua ligand (Figure 4). For *R*-1P1B, *R*-MM, *R*-MM, and *S*-EM, BF_4_^–^ anions exhibit C–H···F (3.16∼3.40 Å) interactions with bipy C–H moieties (Figure 4a–d,f). For *R*-EM, BF_4_^–^ formed C–H···F (3.26 Å) interactions with the C–H moieties on the chiral center of *S*-IDEC (Figure 4c). Conversely, in the presence of *S*-EM, BF_4_^–^ engages in C–H···F (3.41 Å) H-bonding with *S*-IDEC phenyl rings (Figure 4e).

In **CMOM-5[BF**_**4**_**]-***R***-MM** and **CMOM-5[BF**_**4**_**]-***R***-EM**, the homochiral guests were found to be 2-fold disordered (1:1) around the crystallographic inversion center.^58^ Site occupancy was verified by ^1^H NMR with bipy/*S*-IDEC/MM and bipy/*S*-IDEC/EM ratios of 1:1:0.55 (Figures S36 and S38). Site occupancies of the other chiral molecules were found to be 100% (Figures S34, S35, S37, and S39). Chiral guest molecules interact with bipy and *S*-IDEC ligands on the host framework through C–H···π interactions (Figures S14–S19). In the binding sites of *R*-1P1B and *S*-1P1B, the hydroxyl moiety of the 1P1B isomers interacts with a BF_4_^–^ anion through O–H···F H-bonds (3.13 and 3 Å, Figure 4a,d and Tables S14 and S15). The methylene group of *R*-1P1B interacts with the BF_4_^–^ anion through an O–H···F hydrogen bond (3.37 Å). In **CMOM-5[BF**_**4**_**]-***S***-1P1B**, the methanetriyl group of *S*-IDEC interacts with the hydroxyl group of *S*-1P1B through C–H···O H-bonding (3.15 Å). The channels of **CMOM-5[BF**_**4**_**]-***R***-1P1B** were fully occupied by *R*-1P1B molecules and BF_4_^–^ anions, while the disordered solvent molecules in **CMOM-5[BF**_**4**_**]-***S***-1P1B** left a 9.6% void space.^53^ The unit cell volume of **CMOM-5[BF**_**4**_**]-***R***-1P1B** (2863.00 Å^3^) is the smallest among the phases with chiral guest molecules, 93.8% that of **CMOM-[BF**_**4**_**]-***S***-1P1B**, which has the largest unit cell (3052.91 Å^3^, Figure S9 and Table S4). In the *R*-MM and *S*-MM binding sites, the hydroxyl groups of the chiral guests bind to aqua ligands through O–H···O H-bonds (2.63 and 2.77 Å, Figure 4b,e and Tables S16 and S17). Both *R*-MM and *S*-MM bind with the host framework through C–H···π and O–H···π interactions (Figures S16 and S17). In **CMOM-5[BF**_**4**_**]-***R***-MM,** disordered solvent molecules result in 10.0% void space.^53^ In **CMOM-5[BF**_**4**_**]-***S***-MM**, one *S*-MM molecule interacts with an adjacent *S*-MM through C–H···O H-bonding (3.24 Å, Figure 4e).

In **CMOM-5[BF**_**4**_**]-***R***-EM** and **CMOM-5[BF**_**4**_**]-***S***-EM**, the aqua ligands of the host framework interact with the hydroxyl group of the chiral guests through the O–H···O H-bonds (2.78 and 2.76 Å, Figure 4c,f and Tables S18 and S19). *R*-EM and *S*-EM also bind with bipy and *S*-IDEC through C–H···π interactions (Figures S18 and S19). Each *R*-EM interacts with three BF_4_^–^ anions through the H-bonds of O–H···F and C–H···F (Figure 4c). The methyl group of *S*-EM interacts with a BF_4_^–^ anion through C–H···F H-bonding (Figure 4f). The benzene ring of one *S*-EM interacts with the carbonyl group of another through a C–H···O H-bond (3.32 Å, Figure 4f).

In the structures associated with CCS studies, *S*-MM and *S*-EM engage in guest–guest H-bonding to form chains in the channels (Figure 4g,h). This was not observed in the structures of their enantiomers or in the isomers of 1P1B. The guest–guest H-bonding interactions could facilitate higher loading, and in turn, enable the observed enatioselectivity.^59,60^

## Conclusions

In summary, a Ni-*S*-IDEC RBB-based CMOM, **CMOM-5[BF**_**4**_**]**, was obtained as a variant of **CMOM-5[NO**_**3**_**]** through substitution of NO_3_^–^ by BF_4_^–^. **CMOM-5[BF**_**4**_**]** can serve as an enantioselective physisorbent, exhibiting higher affinity for *S*-MM and *S*-EM than their *R*- enantiomers. The ee value for the EM is much higher than that seen for **CMOM-5[NO**_**3**_**]**. The adaptive nature of **CMOM-5[BF**_**4**_**]** enabled it to serve as a CCS for X-ray crystallographic analysis of both the *R*- and *S*- isomers of 1P1B, MM, and EM. These results suggest that crystal engineering approaches can fine-tune the composition of CMOMs in order to control the enantioselectivity of chiral porous materials.

## Supporting Information Available

Experimental details, supplementary characterization results, additional figures, data analysis, and computational calculations (PDF)

## Abbreviations

CMOMs: chiral metal–organic materials
*S*-IDEC: *S*-indoline-2-carboxylicate
bipy: 4,4′-bipyridine
RBB: rod building block
1P1B: 1-phenyl-1-butanol
MM: methyl mandelate
EM: ethyl mandelate
CCS: chiral crystalline sponge
AI: active ingredient
SCXRD: single-crystal X-ray diffraction
MOMs: metal–organic materials
MOFs: metal–organic frameworks
tpt: tris(4-pyridyl)-1,3,5-triazine
GC: gas chromatography
NMR: nuclear magnetic resonance
DMF: *N*,*N*-dimethylformamide
dia: diamondoid
TGA: thermogravimetric analyses
PXRD: powder X-ray diffraction
CSD: Cambridge Structural Database
ee: enantiomeric excess
HPLC: high-performance liquid chromatography
H-bond: hydrogen bond

## References

[ref1] Ribeiro A. R. L.; Maia A. S.; Ribeiro C.; Tiritan M. E. Analysis of chiral drugs in environmental matrices: Current knowledge and trends in environmental, biodegradation and forensic fields. TrAC Trends Anal. Chem. 2020, 124, 115783 10.1016/j.trac.2019.115783.

[ref2] de Albuquerque N. C. P.; Carrão D. B.; Habenschus M. D.; de Oliveira A. R. M. Metabolism studies of chiral pesticides: A critical review. J. Pharm. Biomed. Anal. 2018, 147, 89–109. 10.1016/j.jpba.2017.08.011. 28844369

[ref3] Lin G.-Q.; You Q.-D.; Cheng J.-F. Chiral Drugs: Chemistry and Biological Action; John Wiley & Sons, 2011.

[ref4] Calcaterra A.; D’Acquarica I. The market of chiral drugs: Chiral switches versus de novo enantiomerically pure compounds. J. Pharm. Biomed. Anal. 2018, 147, 323–340. 10.1016/j.jpba.2017.07.008. 28942107

[ref5] Ye J.; Zhao M.; Niu L.; Liu W. Enantioselective Environmental Toxicology of Chiral Pesticides. Chem. Res. Toxicol. 2015, 28 (3), 325–338. 10.1021/tx500481n. 25643169

[ref6] Deng C.-H.; Song B.-Q.; Lusi M.; Bezrukov A. A.; Haskins M. M.; Gao M.-Y.; Peng Y.-L.; Ma J.-G.; Cheng P.; Mukherjee S.; Zaworotko M. J. Crystal Engineering of a Chiral Crystalline Sponge that Enables Absolute Structure Determination and Enantiomeric Separation. Cryst. Growth Des. 2023, 23 (7), 5211–5220. 10.1021/acs.cgd.3c00446. PMC10326857 37426545

[ref7] Beesley T. E.; Lee J. T. Method Development and Optimization of Enantioseparations Using Macrocylic Glycopeptide Chiral Stationary Phases, Chiral Separation Techniques: A Practical Approach, 3rd; Wiley-VCH, 2007; 1–28.

[ref8] Liu Y.; Wu Z.; Armstrong D. W.; Wolosker H.; Zheng Y. Detection and analysis of chiral molecules as disease biomarkers. Nat. Rev. Chem. 2023, 7, 355 10.1038/s41570-023-00476-z. 37117811 PMC10175202

[ref9] Xu W.; Cheng M.; Zhang S.; Wu Q.; Liu Z.; Dhinakaran M. K.; Liang F.; Kovaleva E. G.; Li H. Recent Advances in Chiral Discrimination on Host–guest Functionalized Interfaces. Chem. Commun. 2021, 57 (61), 7480–7492. 10.1039/D1CC01501J. 34264255

[ref10] Lorenz H.; Seidel-Morgenstern A. Processes to Separate Enantiomers. Angew. Chem., Int. Ed. 2014, 53 (5), 1218–1250. 10.1002/anie.201302823. 24442686

[ref11] Flack H. D.; Bernardinelli G. The Use of X-Ray Crystallography to Determine Absolute Configuration. Chirality 2008, 20 (5), 681–690. 10.1002/chir.20473. 17924422

[ref12] Thompson A. L.; Watkin D. J. X-ray Crystallography and Chirality: Understanding the Limitations. Tetrahedron: Asymmetry 2009, 20 (6–8), 712–717. 10.1016/j.tetasy.2009.02.025.

[ref13] Xiao Y.; Ng S.-C.; Tan T. T. Y.; Wang Y. Recent development of cyclodextrin chiral stationary phases and their applications in chromatography. J. Chromatogr. A 2012, 1269, 52–68. 10.1016/j.chroma.2012.08.049. 22959844

[ref14] Wang X.; Li H.; Quan K.; Zhao L.; Qiu H.; Li Z. Preparation and applications of cellulose-functionalized chiral stationary phases: A review. Talanta 2021, 225, 121987 10.1016/j.talanta.2020.121987. 33592735

[ref15] Shen J.; Okamoto Y. Efficient Separation of Enantiomers Using Stereoregular Chiral Polymers. Chem. Rev. 2016, 116 (3), 1094–1138. 10.1021/acs.chemrev.5b00317. 26402470

[ref16] Li J.; Liu R.; Wang L.; Liu X.; Gao H. Enantioseparation of Chiral Pharmaceuticals by Vancomycin-bonded Stationary Phase and Analysis of Chiral Recognition Mechanism. Chirality 2019, 31 (3), 236–247. 10.1002/chir.23052. 30677171

[ref17] Subramanian G. Chiral Separation Techniques: a Practical Approach; John Wiley & Sons, 2007.

[ref18] Zhang Z.; Zaworotko M. J. Template-directed Synthesis of Metal–organic Materials. Chem. Soc. Rev. 2014, 43 (16), 5444–5455. 10.1039/C4CS00075G. 24831589

[ref19] Cook T. R.; Zheng Y.-R.; Stang P. J. Metal–organic Frameworks and Self-assembled Supramolecular Coordination Complexes: Comparing and Contrasting the Design, Synthesis, and Functionality of Metal–organic Materials. Chem. Rev. 2013, 113 (1), 734–777. 10.1021/cr3002824. 23121121 PMC3764682

[ref20] Furukawa H.; Cordova K. E.; O’Keeffe M.; Yaghi O. M. The Chemistry and Applications of Metal-Organic Frameworks. Science 2013, 341 (6149), 1230444 10.1126/science.1230444. 23990564

[ref21] Kitagawa S.; Kitaura R.; Noro S.-I. Functional Porous Coordination Polymers. Angew. Chem., Int. Ed. 2004, 43 (18), 2334–2375. 10.1002/anie.200300610. 15114565

[ref22] Zhao X.; Wang Y.; Li D.; Bu X.; Feng P. Metal–organic Frameworks for Separation. Adv. Mater. 2018, 30 (37), 1705189 10.1002/adma.201705189. 29582482

[ref23] Li J.-R.; Sculley J.; Zhou H.-C. Metal–organic Frameworks for Separations. Chem. Rev. 2012, 112 (2), 869–932. 10.1021/cr200190s. 21978134

[ref24] Inokuma Y.; Arai T.; Fujita M. Networked Molecular Cages as Crystalline Sponges for Fullerenes and Other Guests. Nat. Chem. 2010, 2 (9), 780–783. 10.1038/nchem.742. 20729900

[ref25] Xiong R.-G.; You X.-Z.; Abrahams B. F.; Xue Z.; Che C.-M. Enantioseparation of Racemic Organic Molecules by a Zeolite Analogue. Angew. Chem., Int. Ed. 2001, 40 (23), 4422–4425. 10.1002/1521-3773(20011203)40:23<4422::AID-ANIE4422>3.0.CO;2-G. 12404434

[ref26] Ramadhar T. R.; Zheng S.-L.; Chen Y.-S.; Clardy J. The Crystalline Sponge Method: MOF Terminal Ligand Effects. Chem. Commun. 2015, 51 (56), 11252–11255. 10.1039/C5CC03840E. PMC4490044 26081991

[ref27] Zigon N.; Duplan V.; Wada N.; Fujita M. Crystalline Sponge Method: X-ray Structure Analysis of Small Molecules by Post-Orientation Within Porous Crystals—Principle and Proof-of-Concept Studies. Angew. Chem., Int. Ed. 2021, 60 (48), 25204–25222. 10.1002/anie.202106265. 34109717

[ref28] Rissanen K. Crystallography of Encapsulated Molecules. Chem. Soc. Rev. 2017, 46 (9), 2638–2648. 10.1039/C7CS00090A. 28379237

[ref29] Metherall J. P.; Carroll R. C.; Coles S. J.; Hall M. J.; Probert M. R. Advanced Crystallisation Methods for Small Organic Molecules. Chem. Soc. Rev. 2023, 52 (6), 1995–2010. 10.1039/D2CS00697A. 36857636

[ref30] Qin J.-S.; Yuan S.; Alsalme A.; Zhou H.-C. Flexible Zirconium MOF as the Crystalline Sponge for Coordinative Alignment of Dicarboxylates. ACS Appl. Mater. Interfaces 2017, 9 (39), 33408–33412. 10.1021/acsami.6b16264. 28165703

[ref31] Yan K.; Dubey R.; Arai T.; Inokuma Y.; Fujita M. Chiral Crystalline Sponges for the Absolute Structure Determination of Chiral Guests. J. Am. Chem. Soc. 2017, 139 (33), 11341–11344. 10.1021/jacs.7b06607. 28783333

[ref32] Gong W.; Chen Z.; Dong J.; Liu Y.; Cui Y. Chiral Metal–organic Frameworks. Chem. Rev. 2022, 122 (9), 9078–9144. 10.1021/acs.chemrev.1c00740. 35344663

[ref33] Han Z.; Shi W.; Cheng P. Synthetic strategies for chiral metal-organic frameworks. Chin. Chem. Lett. 2018, 29 (6), 819–822. 10.1016/j.cclet.2017.09.050.

[ref34] Pan M.; Wu K.; Zhang J.-H.; Su C.-Y. Chiral metal–organic cages/containers (MOCs): From structural and stereochemical design to applications. Coord. Chem. Rev. 2019, 378, 333–349. 10.1016/j.ccr.2017.10.031.

[ref35] Liu J.; Mukherjee S.; Wang F.; Fischer R. A.; Zhang J. Homochiral Metal–Organic Frameworks for Enantioseparation. Chem. Soc. Rev. 2021, 50 (9), 5706–5745. 10.1039/D0CS01236J. 33972960

[ref36] Yoon M.; Srirambalaji R.; Kim K. Homochiral Metal–organic Frameworks for Asymmetric Heterogeneous Catalysis. Chem. Rev. 2012, 112 (2), 1196–1231. 10.1021/cr2003147. 22084838

[ref37] Wang H.-S.; Wei J.-P. Emerging Enantiomeric Resolution Materials with Homochiral Nano-fabrications. Nanoscale 2015, 7 (28), 11815–11832. 10.1039/C5NR03048J. 26119977

[ref38] Schnitzer T.; Paenurk E.; Trapp N.; Gershoni-Poranne R.; Wennemers H. Peptide–metal Frameworks with Metal Strings Guided by Dispersion Interactions. J. Am. Chem. Soc. 2021, 143 (2), 644–648. 10.1021/jacs.0c11793. 33417437

[ref39] Gu Z.-G.; Zhan C.; Zhang J.; Bu X. Chiral Chemistry of Metal–camphorate Frameworks. Chem. Soc. Rev. 2016, 45 (11), 3122–3144. 10.1039/C6CS00051G. 27021070

[ref40] Zhang S.-Y.; Wojtas L.; Zaworotko M. J. Structural Insight into Guest Binding Sites in a Porous Homochiral Metal-Organic Material. J. Am. Chem. Soc. 2015, 137 (37), 12045–12049. 10.1021/jacs.5b06760. 26343355

[ref41] Zhang S.-Y.; Yang C.-X.; Shi W.; Yan X.-P.; Cheng P.; Wojtas L.; Zaworotko M. J. A Chiral Metal-Organic Material that Enables Enantiomeric Identification and Purification. Chem 2017, 3 (2), 281–289. 10.1016/j.chempr.2017.07.004.

[ref42] Zhang S.-Y.; Fairen-Jimenez D.; Zaworotko M. J. Structural Elucidation of the Mechanism of Molecular Recognition in Chiral Crystalline Sponges. Angew. Chem., Int. Ed. 2020, 59 (40), 17600–17606. 10.1002/anie.202006438. PMC7540565 32589318

[ref43] Zhang S.-Y.; Jensen S.; Tan K.; Wojtas L.; Roveto M.; Cure J.; Thonhauser T.; Chabal Y. J.; Zaworotko M. J. Modulation of Water Vapor Sorption by a Fourth-generation Metal–organic Material with a Rigid Framework and Self-Switching Pores. J. Am. Chem. Soc. 2018, 140 (39), 12545–12552. 10.1021/jacs.8b07290. 30196697

[ref44] Weckbecker A.; Gröger H.; Hummel W. Regeneration of Nicotinamide Coenzymes: Principles and Applications for the Synthesis of Chiral Compounds Adv. Biochem. Eng. Biotechnol., 2010 120; 195–242. 10.1007/10_2009_55. 20182929

[ref45] Lowe R. F.; Nelson J.; Dang T. N.; Crowe P. D.; Pahuja A.; Mccarthy J. R.; Grigoriadis D. E.; Conlon P.; Saunders J.; Chen; Szabo T.; Chen T. K.; Bozigian H. Rational Design, Synthesis, and Structure–activity Relationships of Aryltriazoles as Novel Corticotropin-releasing Factor-1 Receptor Antagonists. J. Med. Chem. 2005, 48 (5), 1540–1549. 10.1021/jm049339c. 15743196

[ref46] Mori Y.; Ogawa Y.; Mochizuki A.; Nakamura Y.; Fujimoto T.; Sugita C.; Miyazaki S.; Tamaki K.; Nagayama T.; Nagai Y.; Inoue S.-i.; Chiba K.; Nishi T. Synthesis And Optimization of Novel (3S,5R)-5-(2,2-Dimethyl-5-Oxo-4-Phenylpiperazin-1-Yl)Piperidine-3-Carboxamides as Orally Active Renin Inhibitors. Bioorgan. Med. Chem. 2013, 21 (18), 5907–5922. 10.1016/j.bmc.2013.06.057. 23886807

[ref47] Ghosh A. K.; Yadav M. Highly Diastereoselective Intramolecular Asymmetric Oxidopyrylium-olefin [5 + 2] Cycloaddition and Synthesis of 8-Oxabicyclo[3.2.1]Oct-3-Enone Containing Ring Systems. J. Org. Chem. 2021, 86 (12), 8127–8142. 10.1021/acs.joc.1c00600. 34015224 PMC8919378

[ref48] Lizzadro L.; Spieß O.; Schinzer D. Total Synthesis of (−)-disorazole C1. Org. Lett. 2021, 23 (12), 4543–4547. 10.1021/acs.orglett.1c01123. 34037403

[ref49] Kim J.-H.; Yang H.; Park J.; Boons G.-J. A General Strategy for Stereoselective Glycosylations. J. Am. Chem. Soc. 2005, 127 (34), 12090–12097. 10.1021/ja052548h. 16117550

[ref50] Dolomanov O. V.; Bourhis L. J.; Gildea R. J.; Howard J. A. K.; Puschmann H. OLEX2: a Complete Structure Solution, Refinement and Analysis Program. J. Appl. Crystallogr. 2009, 42, 339–341. 10.1107/S0021889808042726.

[ref51] Sheldrick G. M. SHELXT – Integrated space-group and crystalstructure determination. Acta Crystallogr., Sect. A: Found. Adv. 2015, A 71, 3–8. 10.1107/S2053273314026370. PMC4283466 25537383

[ref52] Sheldrick G. M. Crystal structure refinement with SHELXL. Acta Crystallogr. Sect. C Struct. Chem. 2015, C 71, 3–8. 10.1107/S2053229614024218. PMC4294323 25567568

[ref53] Spek A. L. PLATON SQUEEZE: A Tool for the Calculation of the Disordered Solvent Contribution to the Calculated Structure Factors. Acta Crystallogr. Sect. C Struct. Chem. 2015, C 71, 9–18. 10.1107/S2053229614024929. 25567569

[ref54] Hoshino M.; Khutia A.; Xing H.; Inokuma Y.; Fujita M. The Crystalline Sponge Method Updated. IUCrJ. 2016, 3 (2), 139–151. 10.1107/S2052252515024379. 27006777 PMC4775162

[ref55] Ning G.-H.; Matsumura K.; Inokuma Y.; Fujita M. A Saccharide-based Crystalline Sponge for Hydrophilic Guests. Chem. Commun. 2016, 52 (43), 7013–7015. 10.1039/C6CC03026B. 27157794

[ref56] Yang Q.; Lama P.; Sen S.; Lusi M.; Chen K.; Gao W.; Shivanna M.; Pham T.; Hosono N.; Kusaka S.; Perry J. J.; Ma S.; Space B.; Barbour L. J.; Kitagawa S.; Zaworotko M. J. Reversible Switching Between Highly Porous and Nonporous Phases of an Interpenetrated Diamondoid Coordination Network That Exhibits Gate-opening at Methane Storage Pressures. Angew. Chem., Int. Ed. 2018, 57 (20), 5684–5689. 10.1002/anie.201800820. 29575465

[ref57] Peng Y.; Gong T.; Cui Y. A homochiral porous metal–organic framework for enantioselective adsorption of mandelates and photocyclizaton of tropolone ethers. Chem. Commun. 2013, 49 (74), 8253–8255. 10.1039/c3cc43549k. 23926598

[ref58] Müller P. Disorder. In Crystal Structure Refinement A Crystallographer’s Guide to SHELXL; Oxford University Press, 2006; 56–59.

[ref59] Tashiro S.; Shionoya M. Novel Porous Crystals with Macrocycle-Based Well-Defined Molecular Recognition Sites. Acc. Chem. Res. 2020, 53 (3), 632–643. 10.1021/acs.accounts.9b00566. 31970991

[ref60] Tian J.; Chen Q.; Jiang F.; Yuan D.; Hong M. Optimizing Acetylene Sorption Through Induced-fit Transformations in a Chemically Stable Microporous Framework. Angew. Chem., Int. Ed. 2023, 62 (7), e202215253 10.1002/anie.202215253. 36524616

