# Supplementary material for: Effect of Extra-Framework Anion Substitution on the Properties of a Chiral Crystalline Sponge
Source: Cryst Growth Des. 2023 Sep 28;23(11):8139–46. doi: 10.1021/acs.cgd.3c00857 (PMC10626566; doi:10.1021/acs.cgd.3c00857)
Supplement: Supplementary file 1 — cg3c00857_si_001.pdf [file cg3c00857_si_001.pdf]

Effect of Extra-Framework Anion Substitution on the Properties of a Chiral Crystalline Sponge

Chenghua Deng,† Bai-Qiao Song,† Debobroto Sensharma,† Mei-Yan Gao,† Andrey A. Bezrukov,† Varvara I. Nikolayenko,† Matteo Lusi,† Soumya Mukherjee,*,† Michael J. Zaworotko*,†

†Bernal Institute, Department of Chemical Sciences, University of Limerick, Limerick V94 T9PX, Ireland. Corresponding authors' emails: Soumya.Mukherjee@ul.ie; xtal@ul.ie

Table of Content

General experimental procedures........1
CSD data mining........5
Crystallographic analysis methods and tables........9
Structural details........15
Thermogravimetric analysis........32
Powder X-ray diffraction analysis........33
Gas sorption measurements........37
Calculation of the Langmuir surface area........39
NMR spectra........40
HPLC methods for the analysis of samples using chiral resolution experiments........50
References........59

General experimental procedures

4,4'-bipyridine (bipy, > 98%), *R*-methyl-mandelate (*R*-MM, ⩾ 98%), *S*-methyl-mandelate (*S*-MM, ⩾ 98%), *R*-ethyl-mandelate (*R*-EM, ⩾ 98%) and *S*-ethyl-mandelate (*S*-EM, ⩾ 98%) were purchased from TCI. *S*-indoline-2-carboxylic acid (*S*-IDECH, 97.0%), Nickel (II) tetrafluoroborate hexahydrate (98.0%) were purchased from Fluorochem. *R*-1-phenyl-1-butanol (*R*-1P1B, 97%) and *S*-1-phenyl-1-butanol (*S*-1P1B, 97%) were purchased from Sigma-Aldrich. *N*,*N*-Dimethylformamide (DMF, for HPLC, ⩾ 99.9%), ethanol (for HPLC, ⩾ 99.8%), acetonitrile (for HPLC, ⩾ 99.9%), methanol (for HPLC, ⩾ 99.9%), ethyl acetate (for HPLC, ⩾ 99.7%), n-hexane (for HPLC, ⩾ 97.0%) and isopropanol (IPA, for HPLC, ⩾ 99.0%) were purchased from Honeywell. All the procured chemicals were used directly without further purification.

(1) The synthesis of $\{[Ni(S\text{-IDEC})(bipy)(DMF)](BF_4)(DMF)\}_n$ and $\{[Ni(S\text{-IDEC})(bipy)(H_2O)](NO_3)\}_n$ (**CMOM-5[NO_3]**) through solvent thermal method.

Crystals of $\{[Ni(S\text{-IDEC})(bipy)(DMF)](BF_4)(DMF)\}_n$ were obtained by the following procedures: $Ni(BF_4)_2{\cdot}6(H_2O)$ (136 mg, 0.4 mmol) and *S*-indoline-2-carboxylic acid (65 mg, 0.4 mmol) were dissolved in 3 mL methanol, and 4,4'-bipyridine (bipy, 63 mg, 0.4 mmol,) was dissolved in 3 mL DMF. The two solutions were mixed in a vial, enclosed the cap, then move to the 85 °C oven for 24 h. Needle shaped blue crystals were obtained, dried crystals were about 200 mg (0.33 mmol), about 82% yield based on Ni.

Crystals of **CMOM-5[NO_3]** were obtained in the same procedure as reported in the literature.[1]

(2) The synthesis of $\{[Ni(S\text{-IDEC})(bipy)(H_2O)](BF_4)(EtOH)_2\}_n$ (**CMOM-5[BF_4]·EtOH**) through solvent exchange.

Crystals of as-synthesized $\{[Ni(S\text{-IDEC})(bipy)(DMF)](BF_4)(DMF)\}_n$ were soaked in 10 mL ethanol for five days, and the ethanol was exchanged by the fresh every day. Figures S1 and S2 show the photos of the crystals in the vial and under microscope.

(3) The close phase $\{[Ni(S\text{-IDEC})(bipy)(H_2O)](BF_4)\}_n$ (activated **CMOM-5[BF_4]**) obtained by single-crystal-to-single-crystal (SCSC) method.

The crystals of **CMOM-5[BF_4]·EtOH** out of the ethanol onto a filer paper, stand still the crystal in fume hood for five minutes to allow the ethanol on the surface volatilize away. Then put the crystals under vacuum through a Micromeritics SmartVacPrep™ for 12 h at room temperature. The activated **CMOM-5[BF_4]** crystals were used for gas sorption experiments.

(4) Chiral Resolution Experiment

The chiral resolution experiments around **CMOM-5[BF_4]** were done by the following procedures: Dissolving the racemic 1P1B (400 μL), MM (400 mg) or EM (400 mg) in 0.5 mL ethanol to prepare racemic solutions. **CMOM-5[BF_4]·EtOH** crystals (100 mg) was immersed into those racemic solutions for 5 days without stirring or shaking. Subsequently, crystals were filtered and washed with ethyl acetate (3 × 1 mL) then n-hexane (3 × 10 mL) to remove the residual chiral substance on the surface of the crystals. Guest molecules were extracted by soaking the crystals in 10 mL methanol for three days, then crystals were filtered and washed with methanol (3 × 10 mL). After the extraction, the crystals were analyzed to be $\{[Ni(S\text{-IDEC})(bipy)(H_2O)](BF_4)(MeOH)_x\}_n$, **CMOM-5[$BF_4$]·MeOH**, the channels of which were occupied with the disordered methanol molecules. The filtrates were combined, and the solvent was removed using a rotary evaporator. The dried fractions were dissolved in 1 mL of IPA for *ee* (enantiomeric excess) analysis.

The chiral resolution experiment of **CMOM-5[NO_3]** towards the racemic of EM was done by the similar procedures as **CMOM-5[BF_4]** with the displace of ethanol by acetonitrile, and **CMOM-5[BF_4]·EtOH** crystals were replaced by acetonitrile exchanged crystals of **CMOM-5[NO_3]**.

The *ee* values of the binary isomers systems were calculated by the following formula:[2]

$$ee = \frac{F_{(+)} - F_{(-)}}{F_{(+)} + F_{(-)}} \times 100\%$$

Herein, *ee* stands for the *ee* (enantiomeric excess) value, F(+) and F(-) stand for the mole or weight fractions of the isomers relatively more and less respectively.

(5) Crystalline Sponge Experiment

Preparing of {[Ni(*S*-IDEC)(bipy)(H_2O)](BF_4)(*R*-1P1B)}$_n$ (**CMOM-5[BF_4]-*R*-1P1B**): A few single crystals of **CMOM-5[BF_4]·EtOH** were soaked in 0.5 mL ethanol which contains 40 mg *R*-1P1B. The screw cap was loosened to enable slow evaporation of ethanol over 3 days.

Preparing of {[Ni(*S*-IDEC)(bipy)(H_2O)](BF_4)(*S*-1P1B)}$_n$ (**CMOM-5[BF_4]-*S*-1P1B**): the same steps as **CMOM-5[BF_4]-*R*-1P1B** with the replacement of *R*-1P1B by *S*-1P1B.

Preparing of {[Ni(*S*-IDEC)(bipy)(H_2O)](BF_4)(*R*-MM)$_{0.5}$}$_n$ (**CMOM-5[BF_4]-*R*-MM**): the same steps as **CMOM-5[BF_4]-*R*-1P1B** with the replacement of *R*-1P1B by *R*-MM.

Preparing of {[Ni(*S*-IDEC)(bipy)(H_2O)](BF_4)(*S*-MM)}$_n$ (**CMOM-5[BF_4]-*S*-MM**): the same steps as **CMOM-5[BF_4]-*R*-1P1B** with the replacement of *R*-1P1B by *S*-MM.

Preparing of {[Ni(*S*-IDEC)(bipy)(H_2O)](BF_4)(*R*-EM)$_{0.5}$}$_n$ (**CMOM-5[BF_4]-*R*-EM**): the same steps as **CMOM-5[BF_4]-*R*-1P1B** with the replacement of *R*-1P1B by *R*-EM.

Preparing of {[Ni(*S*-IDEC)(bipy)(H_2O)](BF_4)(*S*-EM)}$_n$ (**CMOM-5[BF_4]-*S*-EM**): the same steps as **CMOM-5[BF_4]-*R*-1P1B** with the replacement of *R*-1P1B by *S*-EM.

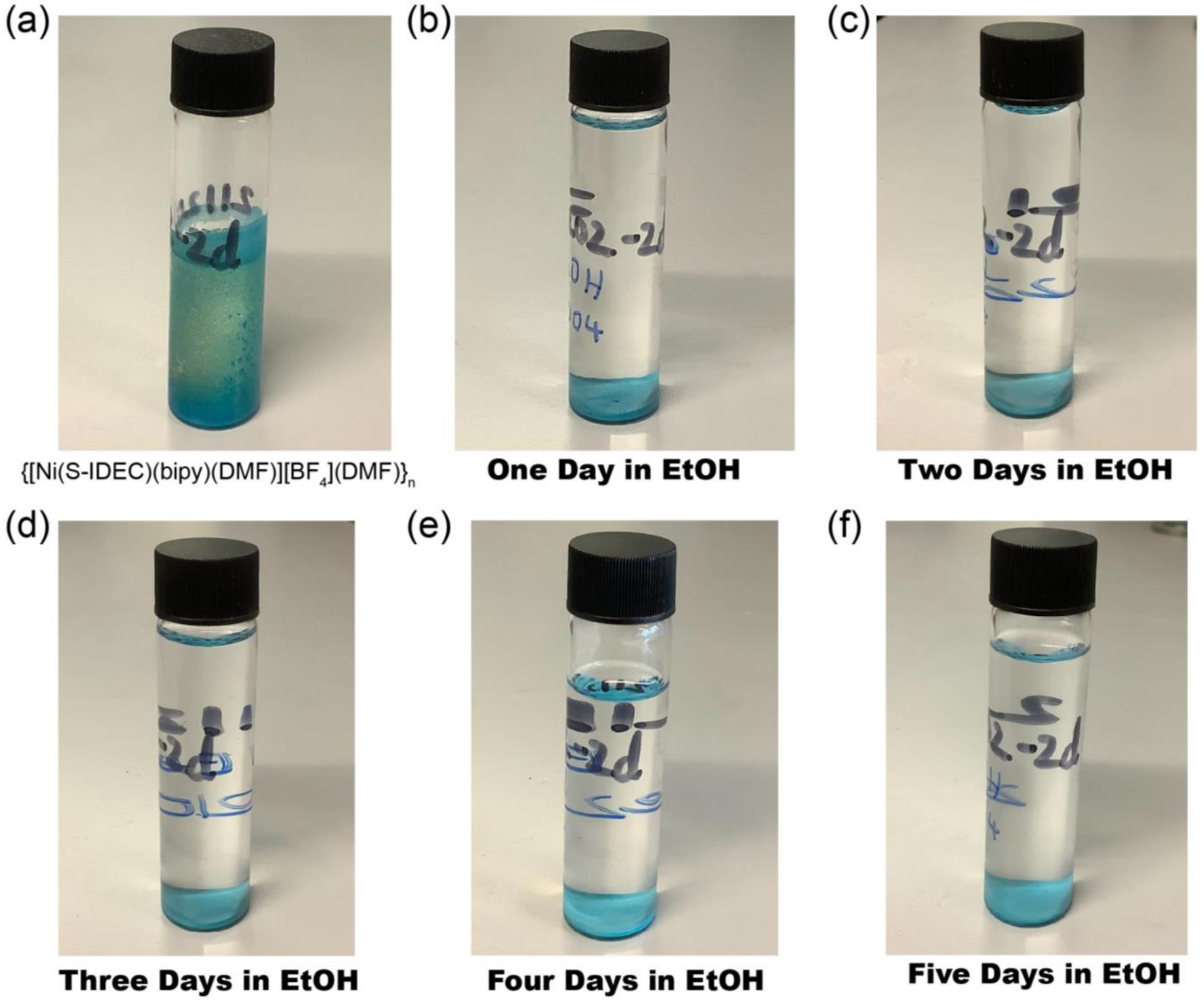

Figure S1. From (a) to (f) are the photos of the crystals in the vial transformed from {[Ni(*S*-IDEC)(bipy)(DMF)](BF_4)(DMF)}$_n$ to **CMOM-5[BF_4]·EtOH** in the 5 days.

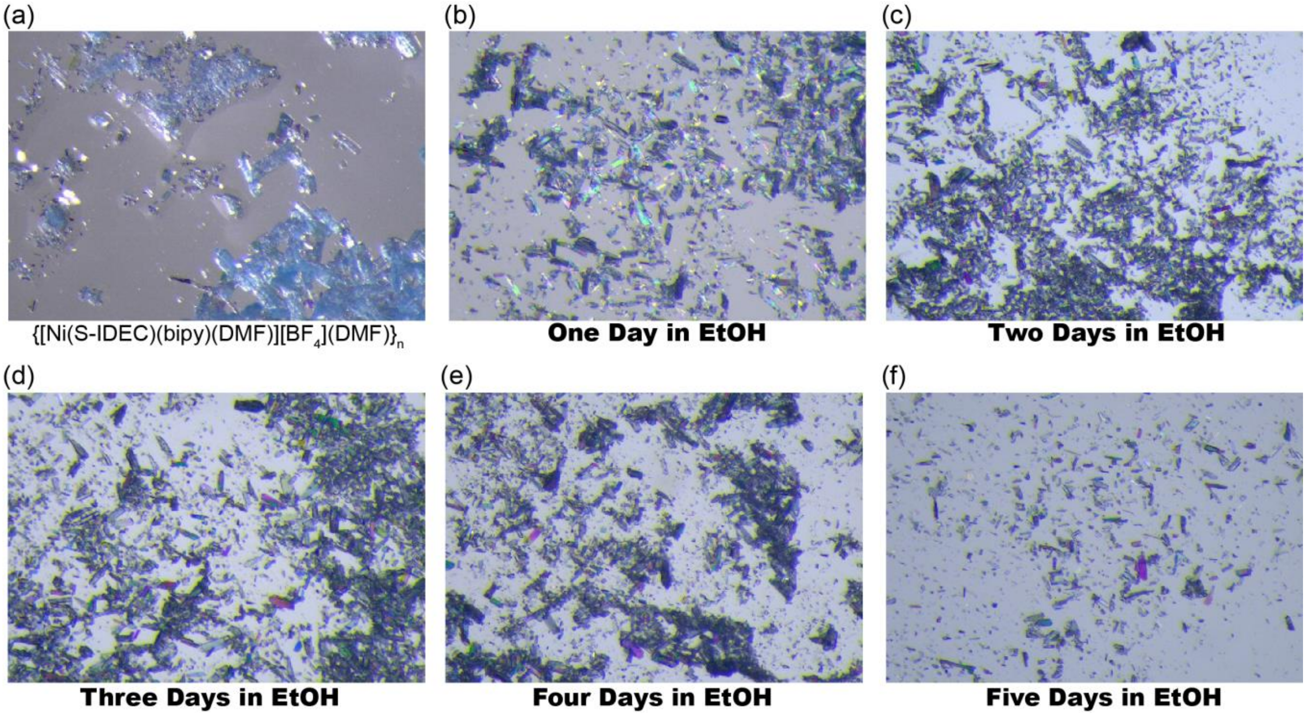

Figure S2. From (a) to (f) are the photos of the crystals of under microscope transformed from {[Ni(*S*-IDEC)(bipy)(DMF)](BF_4)(DMF)}$_n$ to **CMOM-5[BF_4]·EtOH** in the 5 days.

CSD data mining

Cambridge Structural Database (CSD) data mining was conducted through ConQuest software (version 2022.3.0): CSD database version 5.43 (November 2021) and the updates March 2022, June 2022, September 2022 and November 2022.[3]

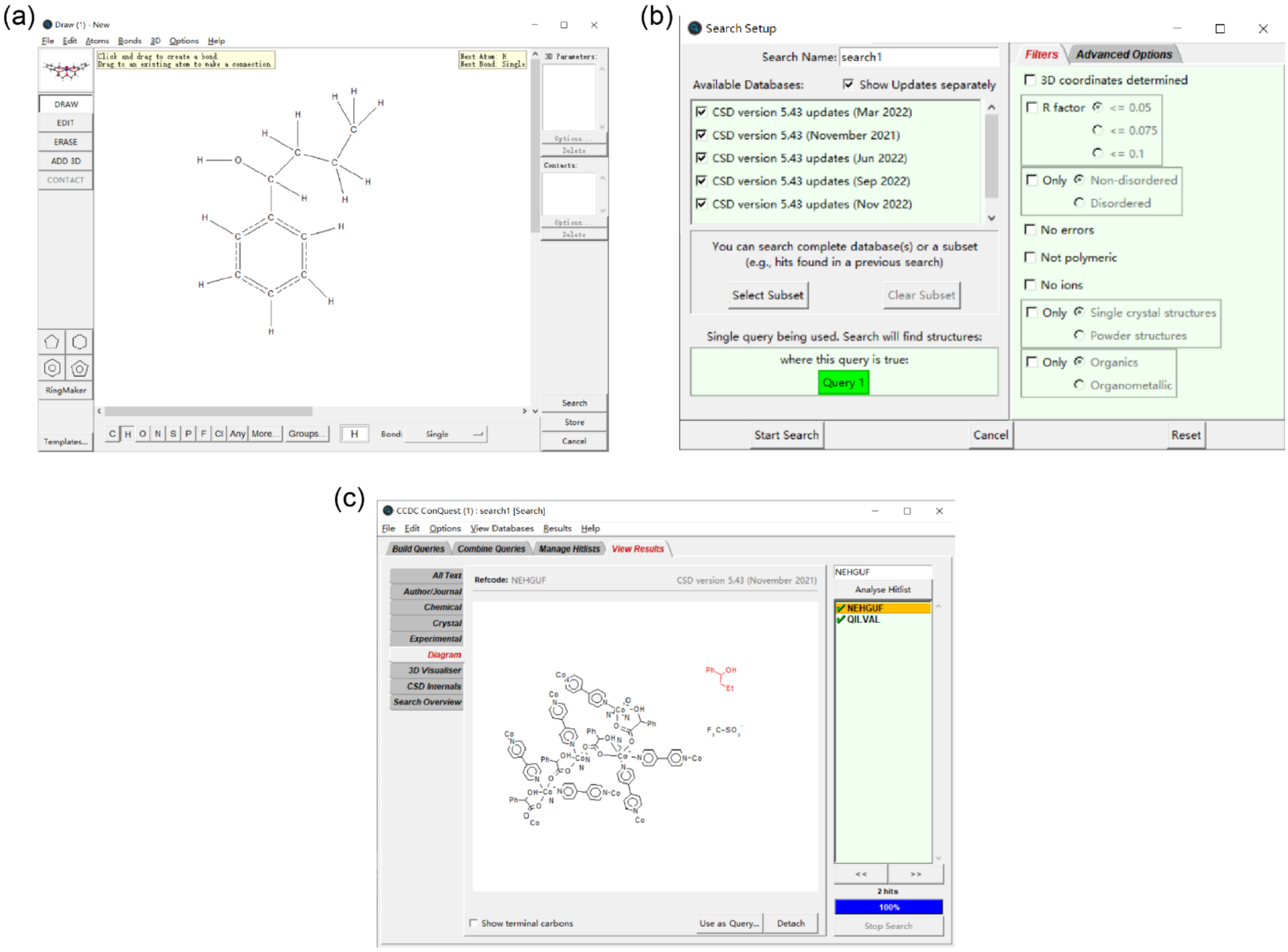

Figure S3. CSD research result on 1P1B contained crystals: (a) the input image; (b) search setup details; (c) search results.

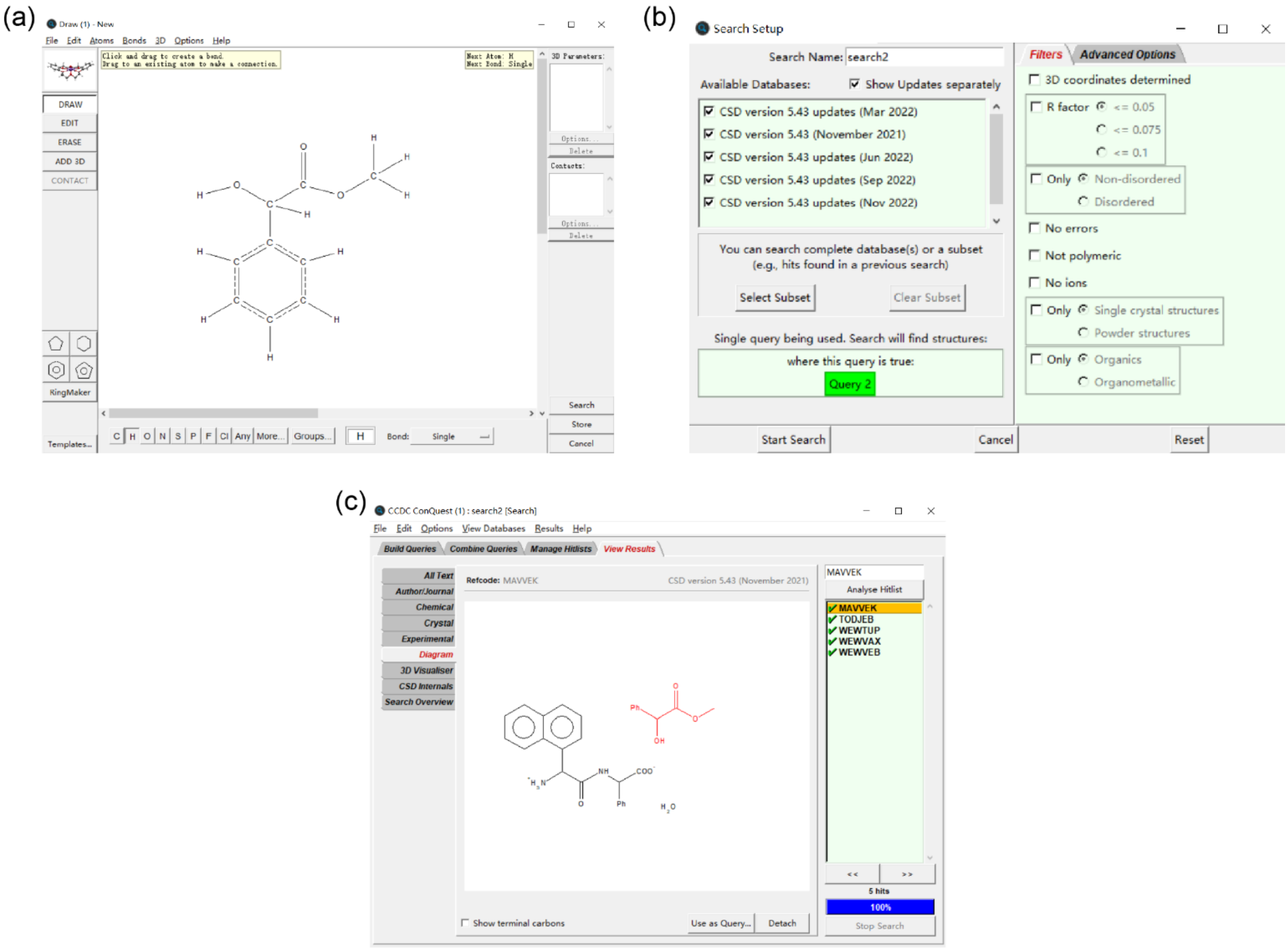

Figure S4. CSD research result on MM contained crystals: (a) the input image; (b) search setup details; (c) search results.

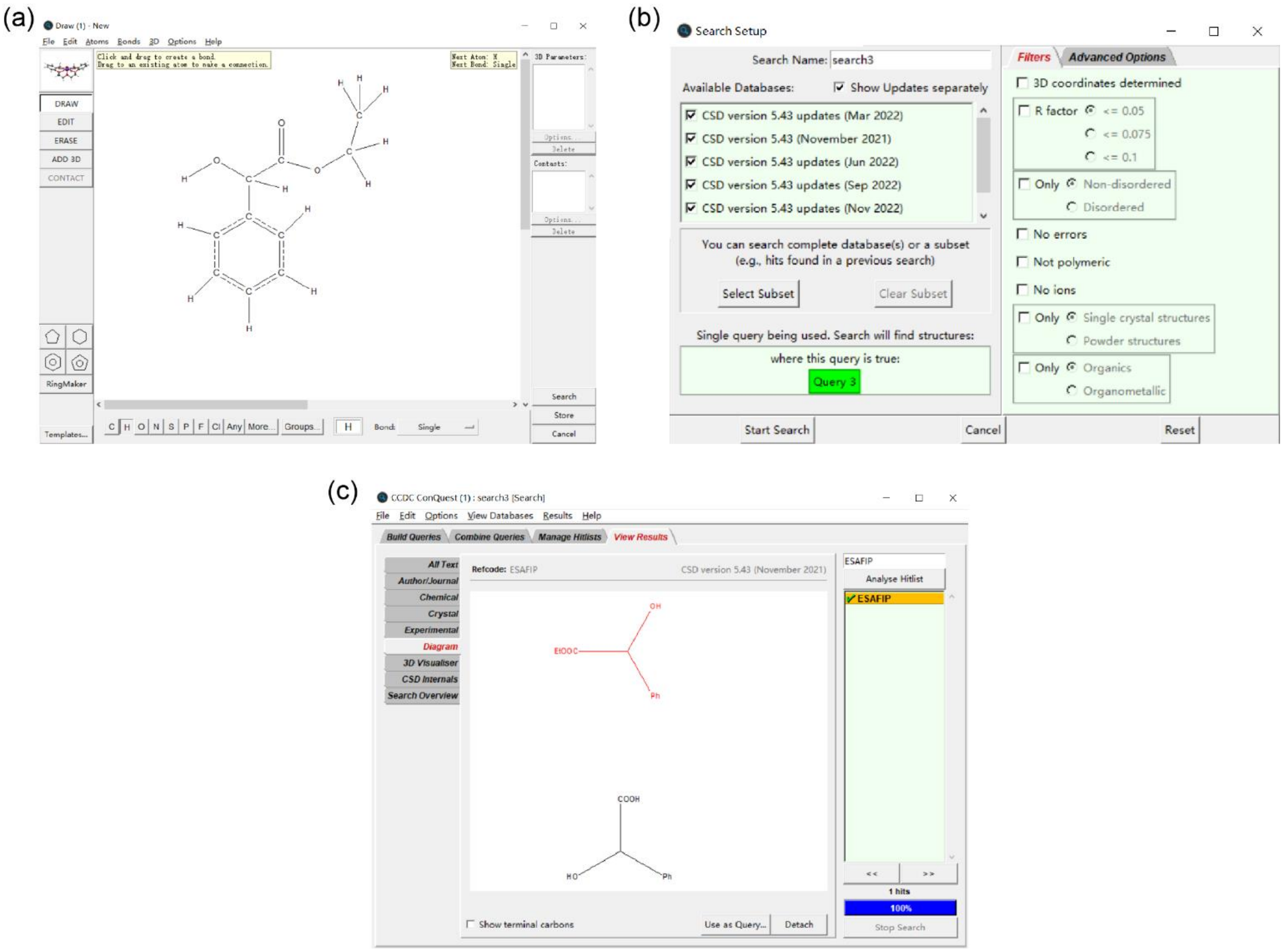

Figure S5. CSD research result on EM contained crystals: (a) the input image; (b) search setup details; (c) search results.

Table S1. Summary of compounds archived in the CSD that containing the chiral guests.

Research Object	Number of Results	Refcode	Diagram	Occupancy	Note	Reference
1P1B	2	NEHGUF	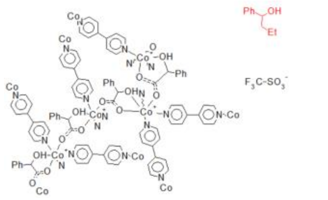	0.88/0.8	*S*-1P1B solved in CMOM-3S	4
		QILVAL	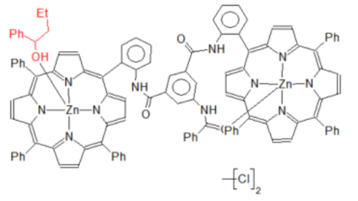	0.8+0.2 (two-fold racemically disordered)	racemate, hydroxy coordinated	5

MM	5	MAVVEK	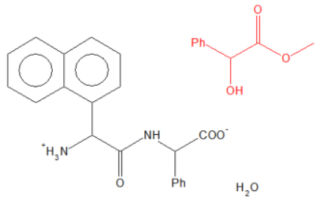	1	*S*-MM, co-crystal	6
		TODJEB	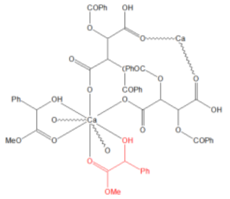	1	*R*-MM, carbonyl and hydroxy coordinated	7
		WEWTUP	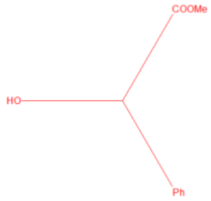	1	R-MM	8
		WEWVAX	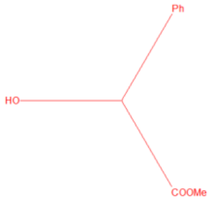	1	*S*-MM	8
		WEWVEB	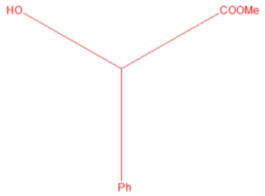	1	Racemic MM	8
EM	1	ESAFIP	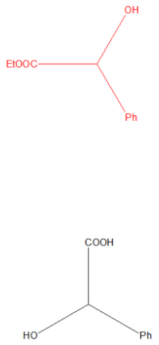	1	Racemic EM, co-crystal	9

Crystallographic analysis methods and tables

Suitable single crystals of all compounds were chosen for single crystal X-ray diffraction measurements. All single crystal data were collected on a Bruker D8 Quest diffractometer equipped with a IµS micro-focus Cu anode CuKα ($\lambda = 1.54178$ Å) and Photon II detector. For low temperature measurements, an open-flow nitrogen attachment from an Oxford Cryosystem was used. The data was indexed, integrated and scaled in APEX4.[10] Absorption correction was performed by multi-scan method using SADABS.[11] Space group determinations were performed with the assistance of XPREP as implemented in APEX4.[12]

Structures were solved using the intrinsic phasing method (SHELXT) and refined on F2 using SHELXL least squares method as run in OLEX2 v1.3 programs packages.[13-15] All non-hydrogen atoms on the frameworks were refined anisotropically. Hydrogen atoms were added geometry at idealized positions and refined using the riding model. The thermal parameters and geometry of the guest molecules were restricted to reasonable limits using the ISOR, SIMU, DFIX, and FLAT instructions. The occupancies of the chiral isomers were determined by taking into consideration of the MASK-calculated electrons and the NMR spectra reflected ratio among the guests and the ligands. Crystallographic details for all compounds are summarized in Crystallographic Tables section. The SQUEEZE subroutine of the PLATON software suite was used to remove the scattering from the highly disordered guest molecules.[16]

Table S2. Crystallographic data of {[Ni(*S*-IDEC)(bipy)(DMF)](BF_4)(DMF)}$_n$ and **CMOM-5[BF_4]·EtOH**.

	{[Ni(*S*-IDEC)(bipy)(DMF)](BF_4)(DMF)}$_n$	**CMOM-5[BF_4]·EtOH**
Formula	$C_{25}H_{30}BF_4N_5NiO_4$	$C_{21}H_{24}BF_4N_3NiO_4$
Formula weight	610.06	527.95
Temperature/K	150.0	150.0
Crystal system	orthorhombic	orthorhombic
Space group	$P2_12_12_1$	$P2_12_12_1$
a/Å	9.9439(2)	10.0422(8)
b/Å	13.8662(3)	15.3344(13)
c/Å	20.4008(4)	19.0147(14)
α/°	90	90
β/°	90	90
γ/°	90	90
Volume/Å^3	2812.95(10)	2928.1(4)
Z	4	4
ρ_{calc}g/cm^3	1.441	1.198
μ/mm^{-1}	1.584	1.425
F(000)	1264.0	1088.0
Crystal size/mm^3	0.168 × 0.142 × 0.124	0.142 × 0.039 × 0.033
Radiation	CuKα (λ = 1.54178)	CuKα (λ = 1.54178)
2θ range for data collection/°	7.708 to 136.698	7.406 to 133.48
Index ranges	$-11 \leq h \leq 11$, $-16 \leq k \leq 16$, $-24 \leq l \leq 24$	$-9 \leq h \leq 11$, $-18 \leq k \leq 12$, $-22 \leq l \leq 19$
Reflections Collected	38694	15705
Independent reflections	5152 [R_{int} = 0.1154, R_{sigma} = 0.0583]	5124 [R_{int} = 0.1238, R_{sigma} = 0.1080]
Data / restraints / parameters	5152/18/408	5124/225/356
Goodness-of-fit on F^2	1.071	1.016
Final R indexes [I>=2σ(I)]	R_1 = 0.0619, wR_2 = 0.1536	R_1 = 0.0791, wR_2 = 0.2025
Final R indexes [all data]	R_1 = 0.0897, wR_2 = 0.1802	R_1 = 0.1026, wR_2 = 0.2241
Largest diff. peak/hole / e Å^{-3}	0.61/-0.63	0.56/-0.72
Flack parameter	0.12(3)	0.14(5)
CCDC number	2266468	2266469

Table S3. Crystallographic data of activated **CMOM-5[BF_4]** and **CMOM-5[BF_4]·MeOH**.

	activated **CMOM-5[BF_4]**	**CMOM-5[BF_4]·MeOH**
Formula	$C_{19}H_{18}BF_4N_3NiO_3$	$C_{19}H_{18}BF_4N_3NiO_3$
Formula weight	481.88	481.88
Temperature/K	150.0	150.0
Crystal system	orthorhombic	orthorhombic
Space group	$P2_12_12_1$	$P2_12_12_1$
a/Å	10.0214(3)	9.9534(5)
b/Å	12.7653(4)	15.4052(8)
c/Å	20.3636(6)	18.7334(9)
α/°	90	90
β/°	90	90
γ/°	90	90
Volume/Å^3	2605.04(14)	2872.5(2)
Z	4	4
ρ_{calc}g/cm^3	1.229	1.114
μ/mm^{-1}	1.527	1.385
F(000)	984.0	984.0
Crystal size/mm^3	0.196 × 0.085 × 0.079	0.168 × 0.055 × 0.049
Radiation	CuKα (λ = 1.54178)	CuKα (λ = 1.54178)
2θ range for data collection/°	8.174 to 136.544	9.442 to 137.286
Index ranges	$-10 \le h \le 12$, $-12 \le k \le 15$, $-24 \le l \le 19$	$-10 \le h \le 11$, $-17 \le k \le 18$, $-22 \le l \le 21$
Reflections Collected	16711	25318
Independent reflections	4739 [R_{int} = 0.0894, R_{sigma} = 0.0731]	5259 [R_{int} = 0.1119, R_{sigma} = 0.0757]
Data / restraints / parameters	4739/72/273	5259/80/281
Goodness-of-fit on F^2	1.052	1.023
Final R indexes [I>=2σ(I)]	R_1 = 0.0610, wR_2 = 0.1527	R_1 = 0.0807, wR_2 = 0.2159
Final R indexes [all data]	R_1 = 0.0904, wR_2 = 0.1744	R_1 = 0.0964, wR_2 = 0.2361
Largest diff. peak/hole / e Å^{-3}	0.62/-0.62	0.92/-0.76
Flack parameter	0.05(3)	0.21(4)
CCDC number	2266470	2266471

Table S4. Crystallographic data of **CMOM-5[BF$_4$]-*R*-1P1B** and **CMOM-5[BF$_4$]-*S*-1P1B**.

	**CMOM-5[BF$_4$]-*R*-1P1B**	**CMOM-5[BF$_4$]-*S*-1P1B**
Formula	$C_{29}H_{32}BF_4N_3NiO_4$	$C_{29}H_{32}BF_4N_3NiO_4$
Formula weight	632.09	632.09
Temperature/K	150.0	150.0
Crystal system	orthorhombic	orthorhombic
Space group	$P2_12_12_1$	$P2_12_12_1$
a/Å	10.0473(9)	10.0986(2)
b/Å	15.9078(14)	15.9258(4)
c/Å	17.9127(17)	18.9824(4)
α/°	90	90
β/°	90	90
γ/°	90	90
Volume/Å^3	2863.0(5)	3052.91(12)
Z	4	4
ρ_{calc}g/cm^3	1.466	1.375
μ/mm^{-1}	1.559	1.462
F(000)	1312.0	1312.0
Crystal size/mm^3	0.165 × 0.083 × 0.051	0.248 × 0.065 × 0.053
Radiation	CuKα (λ = 1.54178)	CuKα (λ = 1.54178)
2θ range for data collection/°	9.876 to 133.198	7.246 to 133.336
Index ranges	$-11 \le h \le 11, -18 \le k \le 18, -21 \le l \le 21$	$-11 \le h \le 10, -18 \le k \le 14, -21 \le l \le 22$
Reflections Collected	27314	22951
Independent reflections	5047 [R_{int} = 0.1602, R_{sigma} = 0.1059]	5379 [R_{int} = 0.0821, R_{sigma} = 0.0629]
Data / restraints / parameters	5047/0/382	5379/178/374
Goodness-of-fit on F^2	1.055	1.042
Final R indexes [I>=2σ(I)]	R_1 = 0.0962, wR_2 = 0.2490	R_1 = 0.0913, wR_2 = 0.2406
Final R indexes [all data]	R_1 = 0.1142, wR_2 = 0.2714	R_1 = 0.1130, wR_2 = 0.2753
Largest diff. peak/hole / e Å^{-3}	1.07/-0.70	1.26/-0.85
Flack parameter	0.06(6)	0.13(2)
CCDC number	2266472	2266473

Table S5. Crystallographic data of **CMOM-5[BF4]-*R*-MM** and **CMOM-5[BF4]-*S*-MM**.

	**CMOM-5[BF4]-*R*-MM**	**CMOM-5[BF4]-*S*-MM**
Formula	$C_{47}H_{46}B_2F_8N_6Ni_2O_9$	$C_{28}H_{28}BF_4N_3NiO_6$
Formula weight	1129.94	648.05
Temperature/K	150.0	150.0
Crystal system	orthorhombic	orthorhombic
Space group	$P2_12_12_1$	$P2_12_12_1$
a/Å	10.0414(11)	10.0105(4)
b/Å	15.4063(15)	15.8257(7)
c/Å	18.8882(16)	18.2834(8)
α/°	90	90
β/°	90	90
γ/°	90	90
Volume/Å^3	2922.0(5)	2896.5(2)
Z	2	4
ρ_{calc}g/cm^3	1.284	1.486
μ/mm^{-1}	1.480	1.612
F(000)	1160.0	1336.0
Crystal size/mm^3	0.138 × 0.055 × 0.037	0.146 × 0.143 × 0.059
Radiation	CuKα (λ = 1.54178)	CuKα (λ = 1.54178)
2θ range for data collection/°	7.404 to 133.188	7.388 to 136.718
Index ranges	-11 ≤ h ≤ 11, -18 ≤ k ≤ 15, -22 ≤ l ≤ 20	-11 ≤ h ≤ 12, -19 ≤ k ≤ 18, -22 ≤ l ≤ 21
Reflections Collected	20601	28789
Independent reflections	5061 [R_{int} = 0.1539, R_{sigma} = 0.1309]	5287 [R_{int} = 0.1377, R_{sigma} = 0.0879]
Data / restraints / parameters	5061/146/379	5287/13/391
Goodness-of-fit on F^2	0.992	1.047
Final R indexes [I>=2σ(I)]	R_1 = 0.0959, wR_2 = 0.2392	R_1 = 0.0656, wR_2 = 0.1634
Final R indexes [all data]	R_1 = 0.1468, wR_2 = 0.2847	R_1 = 0.1110, wR_2 = 0.2048
Largest diff. peak/hole / e Å^{-3}	0.98/-0.61	0.59/-0.63
Flack parameter	0.11(6)	0.00(3)
CCDC number	2266474	2266475

Table S6. Crystallographic data of **CMOM-5[BF$_4$]-*R*-EM** and **CMOM-5[BF$_4$]-*S*-EM**.

	**CMOM-5[BF$_4$]-*R*-EM**	**CMOM-5[BF$_4$]-*S*-EM**
Formula	$C_{48}H_{48}B_2F_8N_6Ni_2O_9$	$C_{29}H_{30}BF_4N_3NiO_6$
Formula weight	1143.96	662.08
Temperature/K	150.0	150.0
Crystal system	orthorhombic	orthorhombic
Space group	$P2_12_12_1$	$P2_12_12_1$
a/Å	10.0451(3)	9.9958(4)
b/Å	15.6225(5)	16.1541(7)
c/Å	18.8492(7)	18.2732(7)
α/°	90	90
β/°	90	90
γ/°	90	90
Volume/Å^3	2958.00(17)	2950.6(2)
Z	4	4
ρ_{calc}g/cm^3	1.284	1.490
μ/mm^{-1}	1.468	1.595
F(000)	1176.0	1368.0
Crystal size/mm^3	0.136 × 0.041 × 0.033	0.155 × 0.068 × 0.062
Radiation	CuKα (λ = 1.54178)	CuKα (λ = 1.54178)
2θ range for data collection/°	7.35 to 136.594	7.304 to 136.806
Index ranges	$-10 \le h \le 11$, $-18 \le k \le 16$, $-21 \le l \le 22$	$-11 \le h \le 10$, $-19 \le k \le 19$, $-21 \le l \le 22$
Reflections Collected	17380	21860
Independent reflections	5352 [R_{int} = 0.1020, R_{sigma} = 0.0873]	5359 [R_{int} = 0.0979, R_{sigma} = 0.0732]
Data / restraints / parameters	5352/95/376	5359/15/376
Goodness-of-fit on F^2	1.113	1.042
Final R indexes [I>=2σ(I)]	R_1 = 0.0892, wR_2 = 0.2446	R_1 = 0.0698, wR_2 = 0.1747
Final R indexes [all data]	R_1 = 0.1068, wR_2 = 0.2589	R_1 = 0.0859, wR_2 = 0.1909
Largest diff. peak/hole / e Å^{-3}	0.79/-0.95	0.74/-0.69
Flack parameter	0.08(4)	0.12(3)
CCDC number	2266476	2266477

Structural details

Table S7. Comparison of **CMOM-5[BF$_4$]·EtOH** and **CMOM-5[NO$_3$]**.

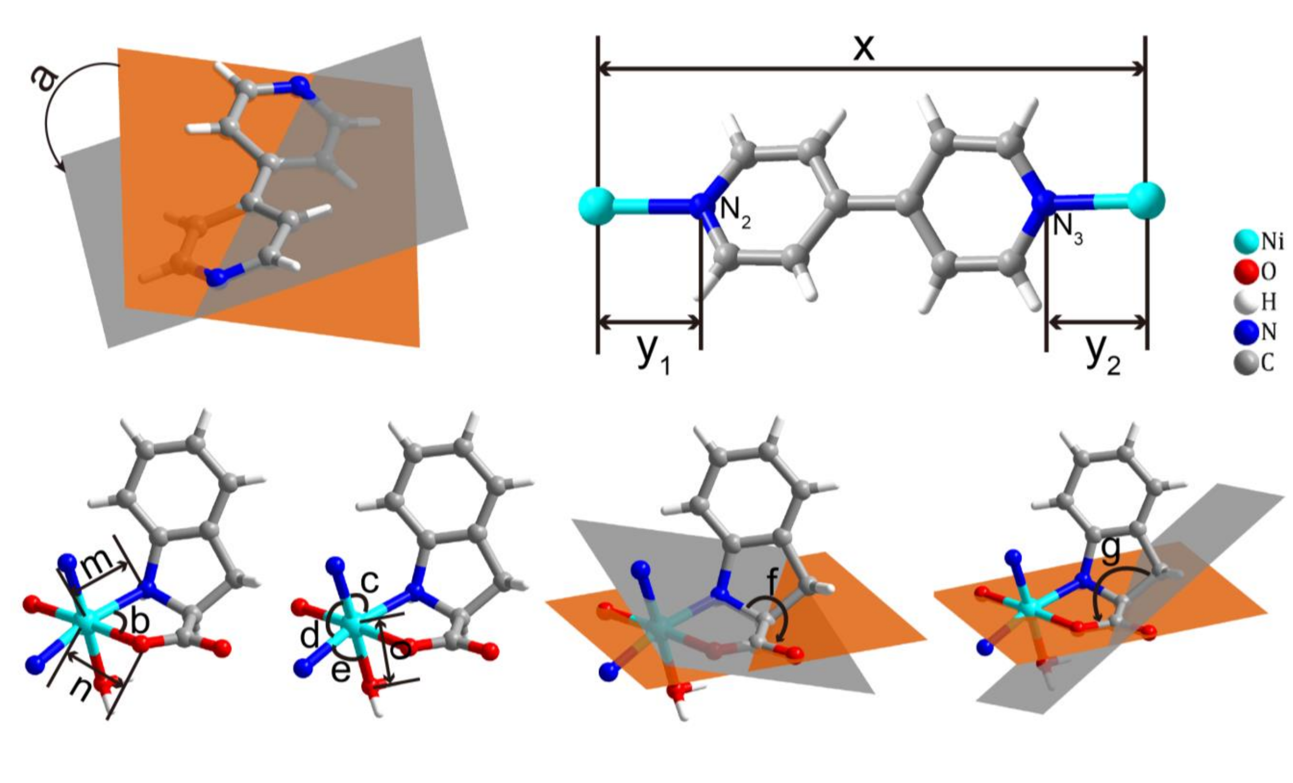

**Compound**		**CMOM-5[BF$_4$]·EtOH**	**CMOM-5[NO$_3$]**
**Formula**		{[Ni(*S*-IDEC)(bipy)(H_2O)](BF_4)($EtOH$)$_2$}$_n$	{[Ni(*S*-IDEC)(bipy)(H_2O)](NO_3)(EtOH)(DMF)$_{1.25}$}$_n$
**Dihedral Angle**	**a (°)**	30.52	31.72
**Bond Angles**	**b (°)**	79.88	80.47
	**c (°)**	97.52	97.10
	**d (°)**	89.46	89.86
	**e (°)**	88.47	88.79
**Torsion Angles**	**f (°, N-C-C-O)**	161.37	163.21
	**g (°, C-C-C-O)**	142.10	137.72
**Distances Between Two Atoms**	**x (Å)**	11.24	11.23
	**y_1 (Å)**	2.07	2.10
	**y_2 (Å)**	2.10	2.08
	**m (Å)**	2.11	2.12
	**n (Å)**	2.05	2.04
	**o (Å)**	2.13	2.12

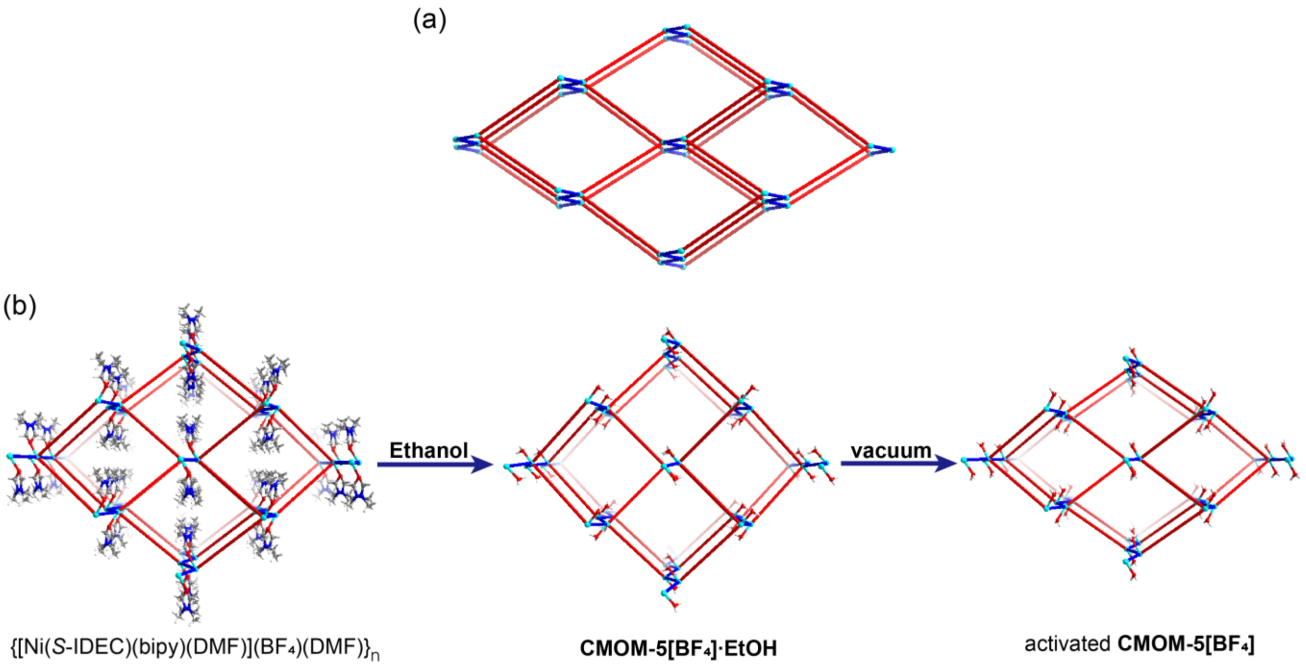

Figure S6. (a) The **dia** topology plot of {[Ni(*S*-IDEC)(bipy)(DMF)](BF_4)(DMF)}$_n$, vertex symbol is {6(2).6(2).6(2).6(2).6(2).6(2)}, point symbol is {6^6}. (b) The structural transformation from {[Ni(*S*-IDEC)(bipy)(DMF)][BF_4](DMF)}$_n$ to **CMOM-5[BF_4]·EtOH** to activated **CMOM-5[BF_4]** illustrated by the topologically simplified nets. The *S*-IDEC was simplified as the blue lines, and the bipy was simplified as the red lines. The counter anions and solvent molecules were omitted for clarity.

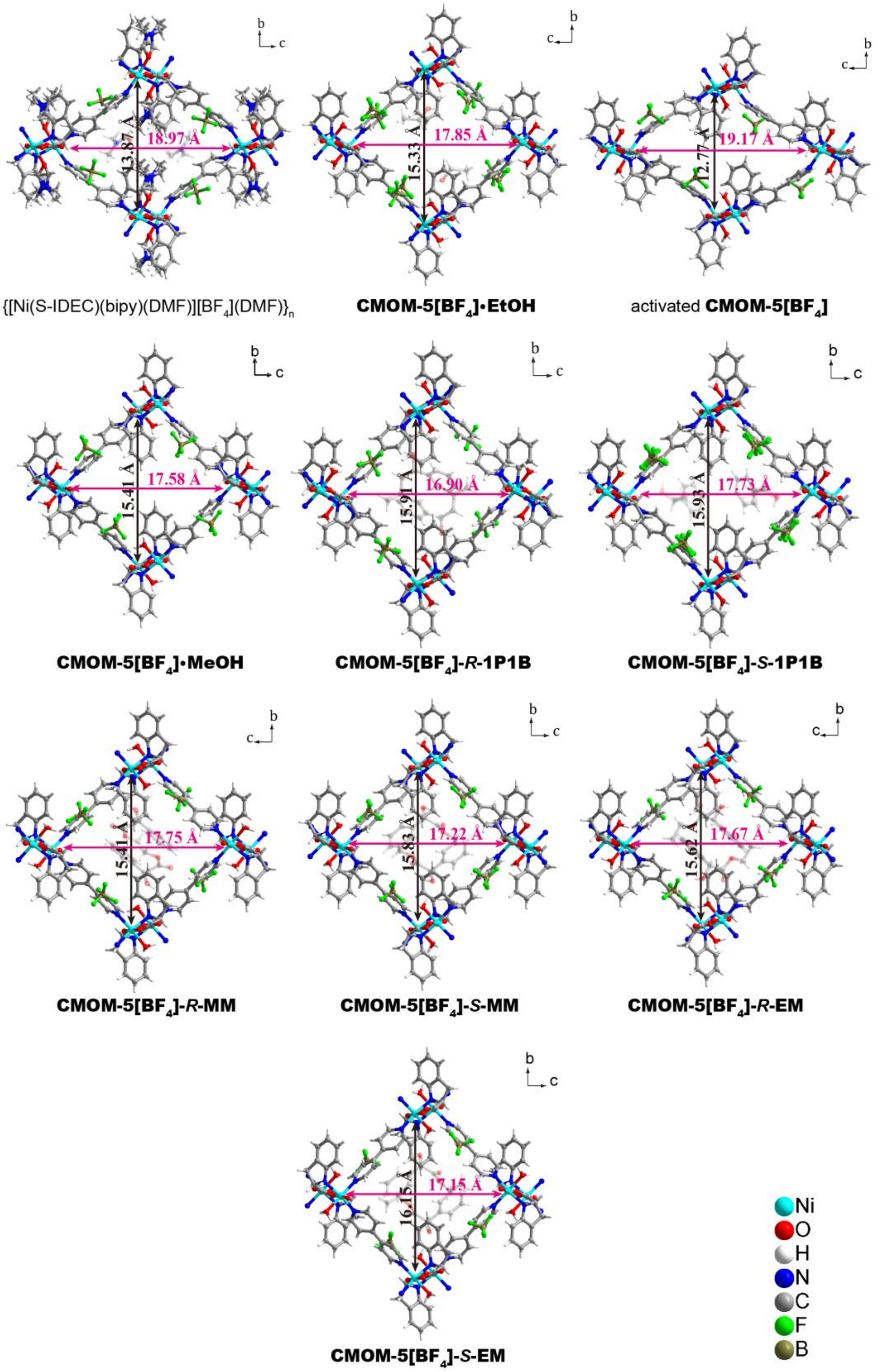

Figure S7. Phases of {[Ni(*S*-IDEC)(bipy)(DMF)](BF_4)(DMF)}$_n$ and **CMOM-5[BF_4]·EtOH** with different guest molecules loaded. Structures of each phase were viewed in the direction of the infinite channels. The distance of the nickel anions on the opposite side of the quadrangular channel was labelled.

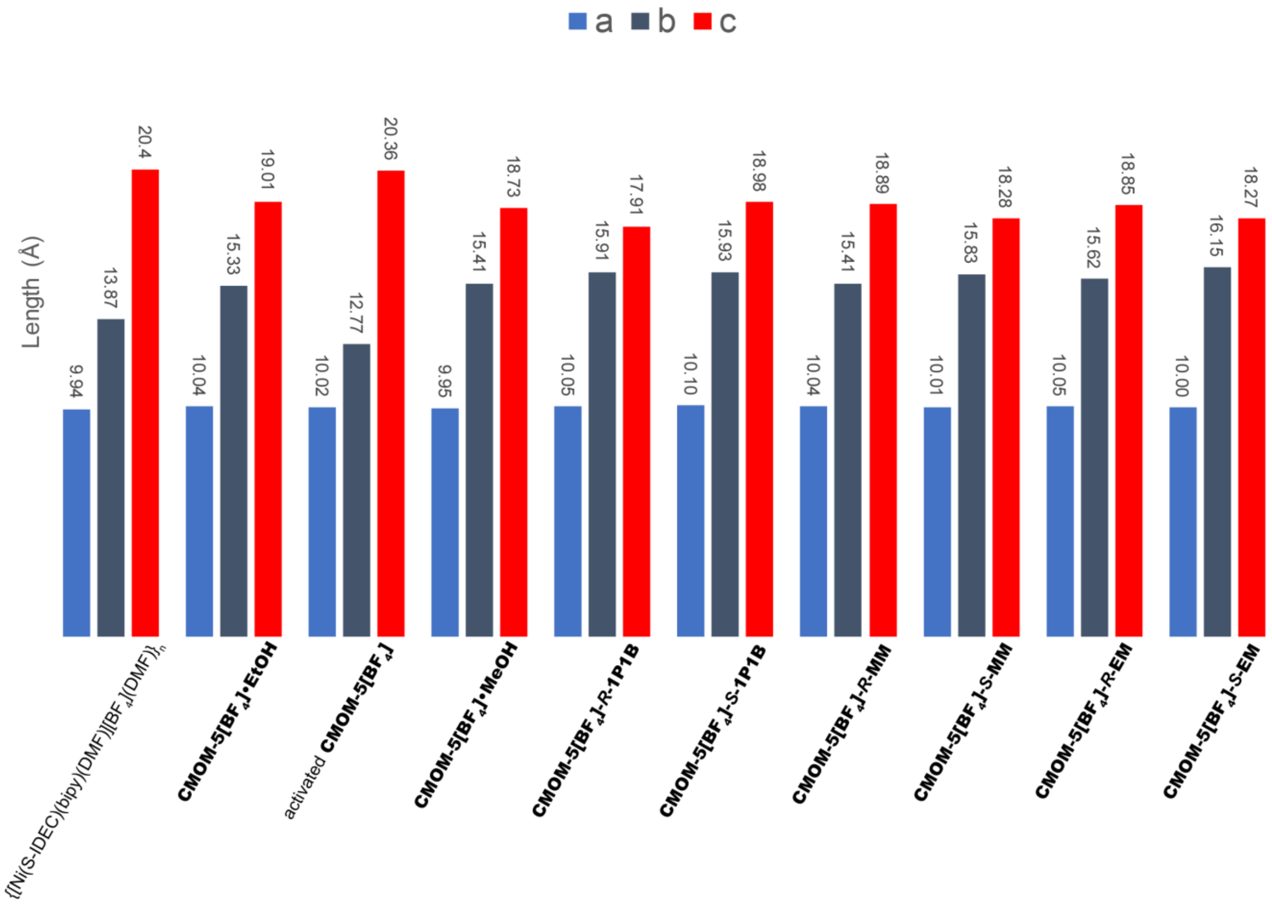

Figure S8. The crystallographic *a*, *b*, *c* parameters of {[Ni(*S*-IDEC)(bipy)(DMF)](BF_4)(DMF)}$_n$ and **CMOM-5[BF_4]** with different guests loaded.

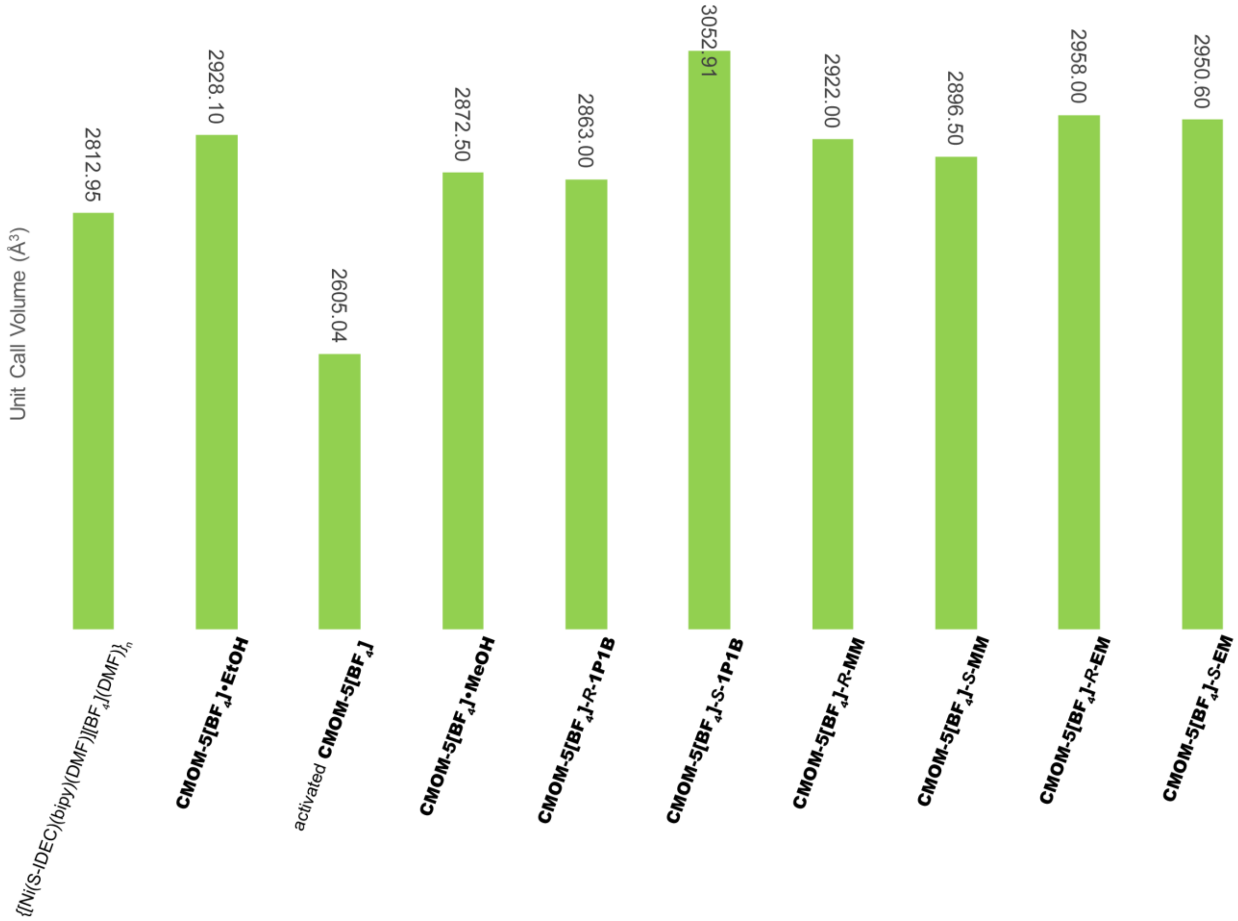

Figure S9. The unit cell volumes of {[Ni(*S*-IDEC)(bipy)(DMF)](BF_4)(DMF)}$_n$ and **CMOM-5[BF_4]** with different guests loaded.

Table S8. Structural information of the coordinated bipy in {[Ni(*S*-IDEC)(bipy)(DMF)](BF$_4$)(DMF)}$_n$ and **CMOM-5[BF$_4$]·EtOH** with different guests loaded.

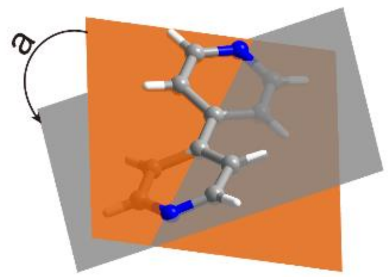

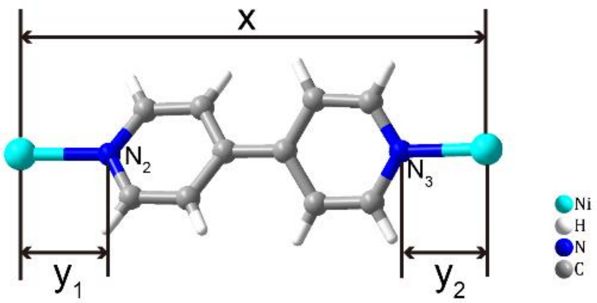

Compound	Dihedral Angle	Distances Between Two Atoms		
	**a (°)**	**x (Å)**	**y$_1$ (Å)**	**y$_2$ (Å)**
{[Ni(*S*-IDEC)(bipy)(DMF)](BF$_4$)(DMF)}$_n$	29.82	11.19	2.10	2.08
**CMOM-5[BF$_4$]·EtOH**	30.52	11.24	2.07	2.10
activated **CMOM-5[BF$_4$]**	35.95	11.10	2.08	2.11
**CMOM-5[BF$_4$]·MeOH**	31.78	11.23	2.06	2.10
**CMOM-5[BF$_4$]-*R*-1P1B**	25.92	11.21	2.09	2.06
**CMOM-5[BF$_4$]-*S*-1P1B**	21.34	11.27	2.09	2.08
**CMOM-5[BF$_4$]-*R*-MM**	29.75	11.24	2.07	2.08
**CMOM-5[BF$_4$]-*S*-MM**	27.50	11.21	2.06	2.07
**CMOM-5[BF$_4$]-*R*-EM**	27.55	11.25	2.09	2.08
**CMOM-5[BF$_4$]-*S*-EM**	25.21	11.23	2.08	2.10

Table S9. Structural information of the coordinated *S*-IDEC in {[Ni(*S*-IDEC)(bipy)(DMF)](BF_4)(DMF)}$_n$ and **CMOM-5[BF$_4$]·EtOH** with different guests loaded.

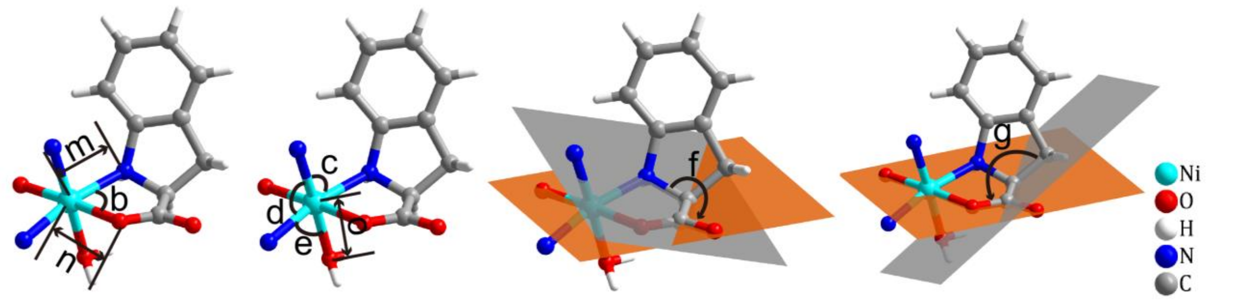

Compound	Bond Angle				Distances Between Two Atoms			Torsion Angles	
	b (°)	c (°)	d (°)	e (°)	m (Å)	n (Å)	o (Å)	f (°, N-C-C-O)	g (°, C-C-C-O)
{[Ni(*S*-IDEC)(bipy)(DMF)](BF_4)(DMF)}$_n$*	79.93	99.51	86.87	88.19*	2.10	2.06	2.11*	165.01	134.10
**CMOM-5[BF$_4$]·EtOH**	79.88	97.52	89.46	88.47	2.11	2.05	2.13	161.37	142.10
activated **CMOM-5[BF$_4$]**	80.03	98.93	86.55	89.54	2.13	2.04	2.14	155.29	141.79
**CMOM-5[BF$_4$]·MeOH**	92.57	88.64	89.10	85.52	2.13	2.05	2.16	157.42	141.39
**CMOM-5[BF$_4$]-*R*-1P1B**	79.63	97.49	89.08	85.89	2.12	2.07	2.16	155.14	144.47
**CMOM-5[BF$_4$]-*S*-1P1B**	80.40	97.59	89.49	88.48	2.12	2.05	2.17	164.96	137.07
**CMOM-5[BF$_4$]-*R*-MM**	80.66	96.76	89.46	88.05	2.11	2.06	2.14	162.20	139.82
**CMOM-5[BF$_4$]-*S*-MM**	80.48	96.55	89.70	88.60	2.14	2.05	2.13	158.15	141.85
**CMOM-5[BF$_4$]-*R*-EM**	80.77	96.54	89.47	88.68	2.13	2.06	2.12	164.37	138.57
**CMOM-5[BF$_4$]-*S*-EM**	80.08	95.22	90.67	88.90	2.14	2.05	2.15	159.59	139.29

*****The oxygen atom in the bond length or bond angle is corresponding to the atom in coordinated DMF.

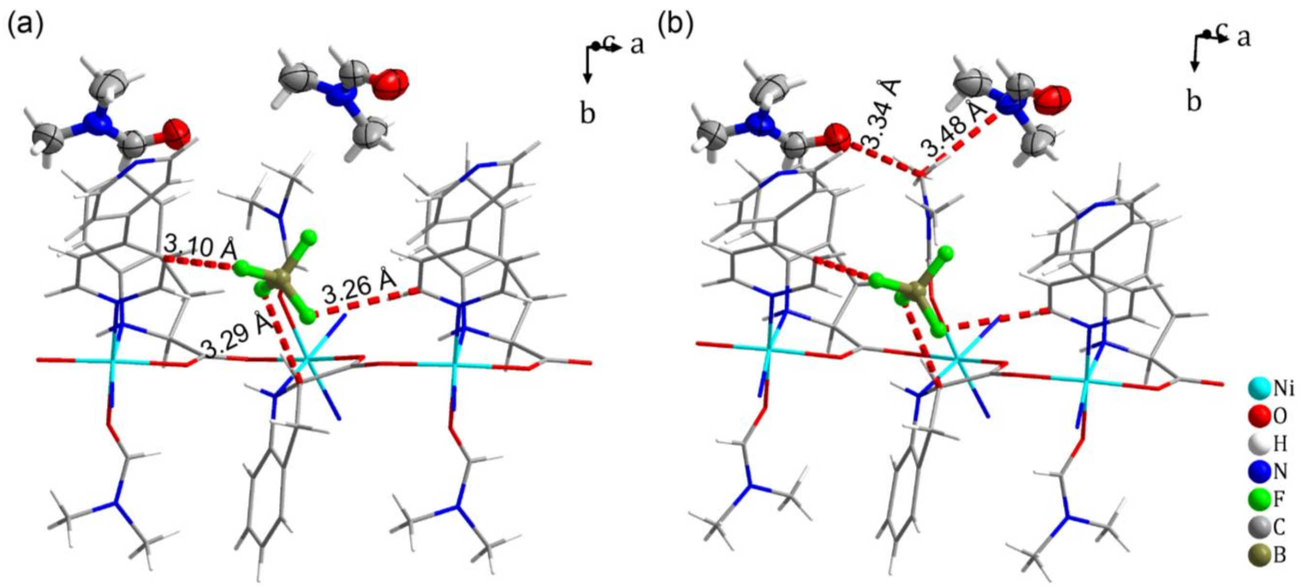

Figure S10. Hydrogen bond interactions among the free DMF, BF_4^- and the cationic framework in $\{[Ni(S\text{-IDEC})(bipy)(DMF)](BF_4)(DMF)\}_n$. (a) and (b) are corresponding to the 2-fold disordered coordination DMF part 1 and part 2. The non-hydrogen atoms of the free DMF were drawn in thermal ellipsoids at 50% possibility. The hydrogen bond interactions were labelled by red dash lines.

Table S10. Hydrogen bond parameters in $\{[Ni(S\text{-IDEC})(bipy)(DMF)](BF_4)(DMF)\}_n$.

Hydrogen bond	Donor-H [Å]	Acceptor-H [Å]	Donor-Acceptor [Å]	Angle [°]	Symmetry operator
N1-H1···O2*	0.86(10)	2.17(10)	2.922(7)	147(7)	1/2+x,3/2-y,1-z
C2-H2···F3	1.00	2.32	3.289(13)	162	1+x,y,z
C3-H3B···O1*	0.99	2.46	2.878(10)	105	
C10-H10···F1	0.95	2.53	3.263(9)	134	
C10-H10···O1*	0.95	2.44	3.042(8)	121	
C13-H13···F3	0.95	2.36	3.102(12)	135	x,1+y,z
C14-H14···O2*	0.95	2.40	3.001(10)	121	-1/2+x,3/2-y,1-z
C17-H17···O2*	0.95	2.44	3.006(9)	118	3/2-x,1-y,1/2+z
C21B-H21B···O3*	0.98	2.44	2.84(2)	104	
C22B-H22A···O4	0.98	2.44	3.34(2)	153	1/2+x,3/2-y,1-z
C22B-H22C···N5	0.98	2.60	3.49(2)	151	x,1+y,z
C24-H24A···O4*	0.98	2.39	2.78(2)	103	

*Intramolecular hydrogen bond.

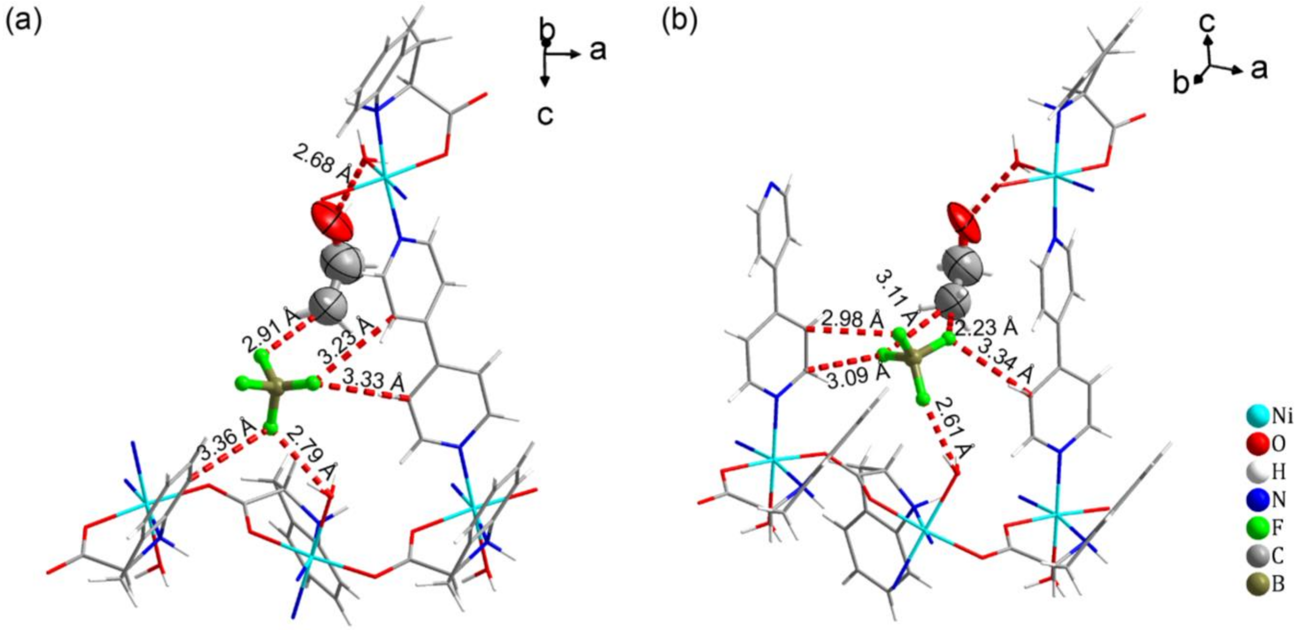

Figure S11. Hydrogen bond interactions among the ethanol, BF_4^- and the cationic framework in **CMOM-5[BF₄]·EtOH**. (a) and (b) are corresponding to the 2-fold disordered BF_4^- part 1 and part 2. The non-hydrogen atoms of the ethanol were drawn in thermal ellipsoids at 50% possibility. The hydrogen bond interactions were labelled by red dash lines.

Table S11. Hydrogen bond parameters in **CMOM-5[BF_4]·EtOH.**

Hydrogen bond	Donor-H [Å]	Acceptor-H [Å]	Donor-Acceptor [Å]	Angle [°]	Symmetry operator
N1-H1···O2*	1.00	2.26	2.987(9)	128	1/2+x,3/2-y,1-z
O3-H3A···F2	0.90	2.14	2.794(19)	128	1/2-x,1-y,1/2+z
O3-H3A···F7	0.90	2.02	2.61(4)	122	1/2-x,1-y,1/2+z
O4-H4···O3	0.84	2.37	2.680(16)	102	1/2-x,1-y,1/2+z
C3-H3C···O1*	0.99	2.45	2.865(13)	105	
C6-H6···F2	0.95	2.49	3.37(2)	154	3/2-x,1-y,1/2+z
C10-H10···O2*	0.95	2.58	3.064(13)	112	-1/2+x,3/2-y,1-z
C10-H10···O1*	0.95	2.60	3.042(12)	109	
C13-H13···F1	0.95	2.49	3.235(19)	135	
C16-H16···F5	0.95	2.46	2.98(3)	115	-1+x,y,z
C17-H17···F8	0.95	2.32	3.09(3)	137	-1+x,y,z
C17-H17···O1*	0.95	2.45	3.043(12)	121	-x,-1/2+y,1/2-z
C18-H18···F1	0.95	2.38	3.328(17)	173	
C18-H18···F6	0.95	2.50	3.34(3)	147	
C19-H19···O2*	0.95	2.36	2.999(13)	124	1/2-x,1-y,-1/2+z
C19-H19···O3*	0.95	2.57	3.432(12)	151	1/2-x,1-y,-1/2+z
C21-H21A···F4	0.98	2.45	2.91(4)	108	
C21-H21A···F8	0.98	2.39	3.11(4)	130	
C21-H21B···F6	0.98	1.81	2.23(4)	103	

*Intramolecular hydrogen bond.

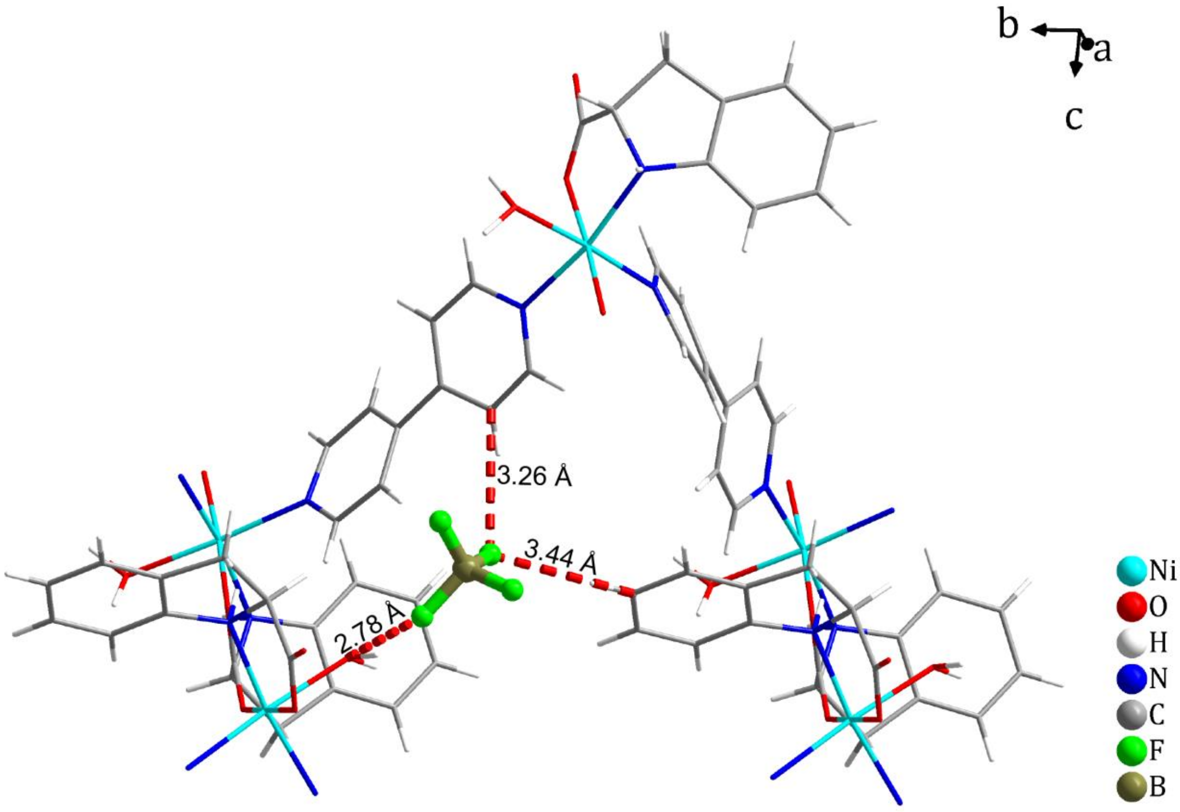

Figure S12. Hydrogen bond interactions among the ethanol, BF_4^- and the cationic framework in activated **CMOM-5[BF4]**. The hydrogen bond interactions were labelled by red dash lines.

Table S12. Hydrogen bond parameters in activated **CMOM-5[BF4].**

Hydrogen bond	Donor-H [Å]	Acceptor-H [Å]	Donor-Acceptor [Å]	Angle [°]	Symmetry operator
N1-H1···O2*	1.00	2.28	3.009(7)	129	1/2+x,3/2-y,1-z
O3-H3A···F2	0.91	1.92	2.782(9)	157	1/2-x,1-y,1/2+z
C3-H3C···O1*	0.99	2.40	2.833(10)	106	
C8-H8···F1	0.95	2.49	3.443(9)	177	1-x,-1/2+y,1/2-z
C10-H10···O1*	0.95	2.55	3.032(9)	111	
C11-H11···F1	0.95	2.39	3.258(11)	151	
C12-H12···O2*	0.95	2.49	2.981(10)	112	-1/2+x,3/2-y,1-z
C17-H17···O1*	0.95	2.48	3.024(9)	116	-x,-1/2+y,1/2-z
C19-H19···O2*	0.95	2.36	2.952(10)	120	1/2-x,1-y,-1/2+z
C19-H19···O3*	0.95	2.51	3.383(9)	152	1/2-x,1-y,-1/2+z

*Intramolecular hydrogen bond.

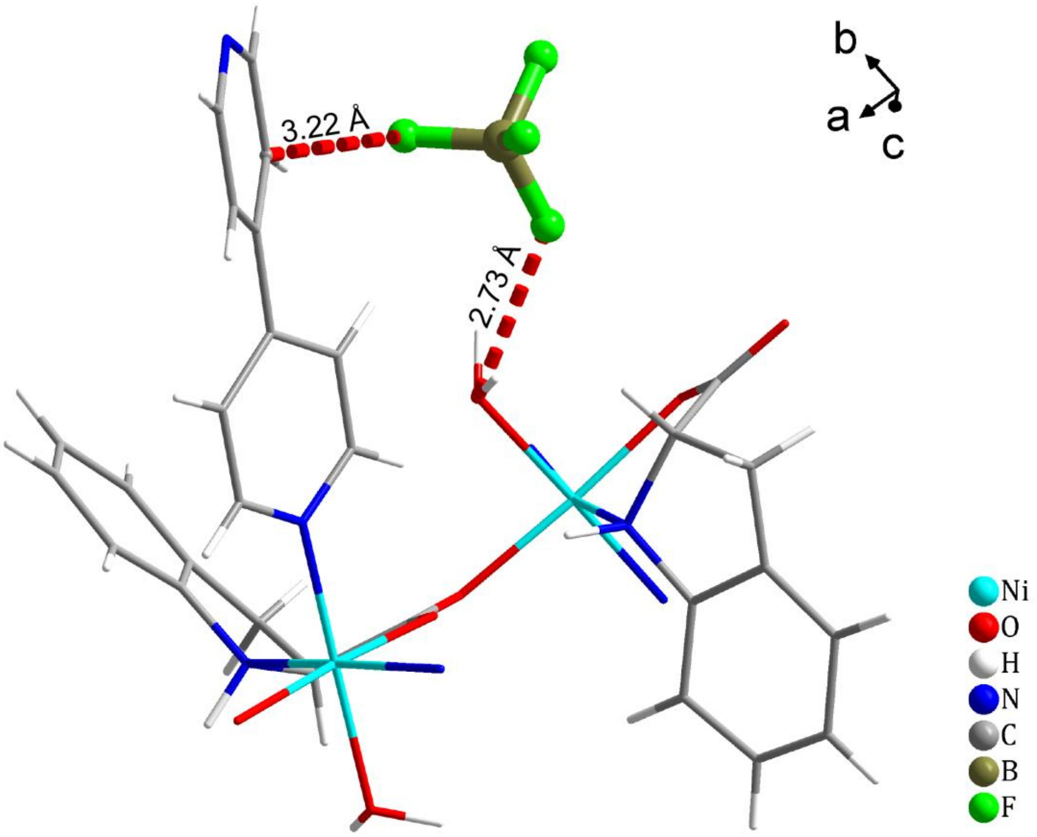

Figure S13. Intermolecular interactions between BF_4^- and the cationic framework in **CMOM-5[BF$_4$]·MeOH**. The hydrogen bond interactions were labelled by red dash lines.

Table S13. Hydrogen bond parameters in **CMOM-5[BF$_4$]·MeOH**.

Hydrogen bond	Donor-H [Å]	Acceptor-H [Å]	Donor-Acceptor [Å]	Angle [°]	Symmetry operator
N1-H1···O2*	1.00	2.20	2.948(8)	130	-1/2+x,3/2-y,1-z
O3-H3A···F1	0.97	2.22	2.725(12)	112	
O3-H3B···F1	0.97	2.25	2.725(12)	109	
C3-H3C···O1*	0.99	2.43	2.858(12)	105	
C10-H10···O1*	0.95	2.59	3.051(11)	110	
C11-H11···F3	0.95	2.40	3.22(3)	144	3/2-x,1-y,-1/2+z
C12-H12···O2*	0.95	2.49	2.997(10)	113	1/2+x,1/2-y,1-z
C17-H17···O1*	0.95	2.44	3.029(11)	120	2-x,1/2+y,1/2-z
C19-H19···O2*	0.95	2.32	2.974(11)	126	3/2-x,1-y,-1/2+z
C19-H19···O3*	0.95	2.52	3.316(11)	141	3/2-x,1-y,-1/2+z

*Intramolecular hydrogen bond.

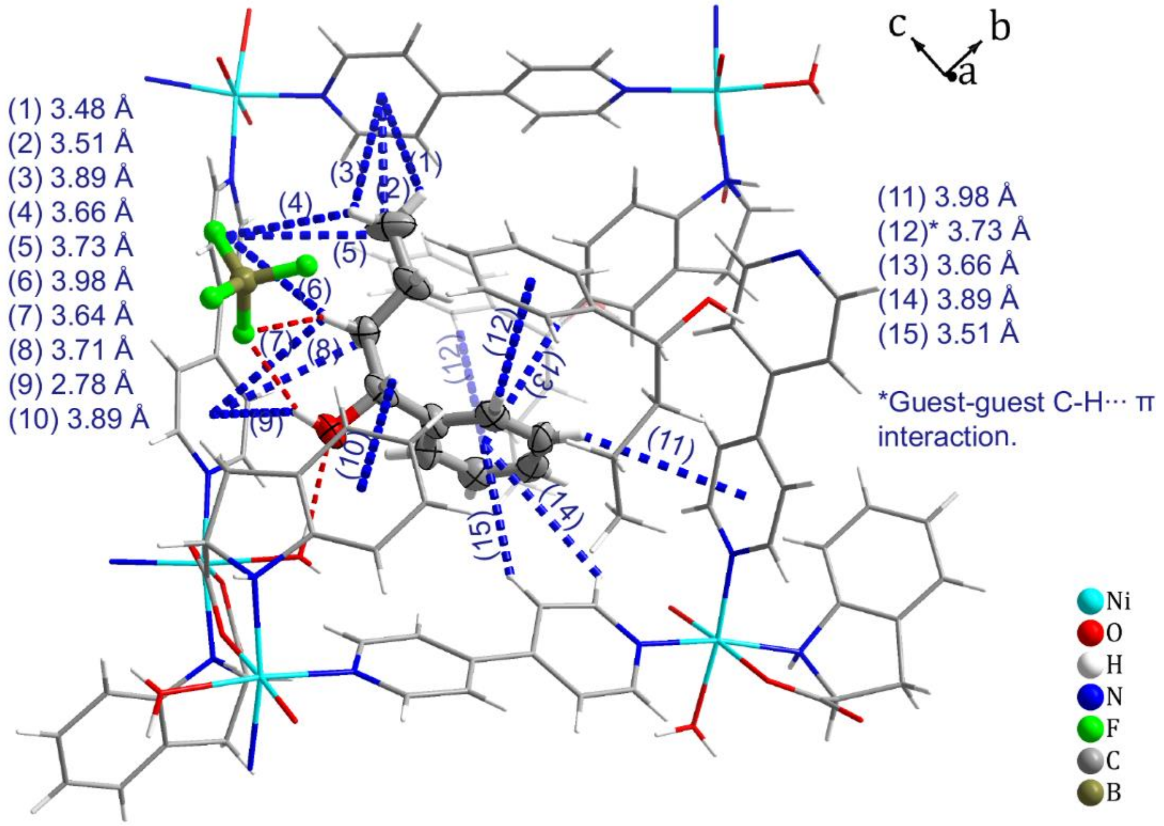

Figure S14. The C-H···π interactions (labelled by blue dash lines) between *R*-1P1B and the host framework in **CMOM-5[BF$_4$]-*R*-1P1B**.

Table S14. Hydrogen bond parameters in **CMOM-5[BF$_4$]-*R*-1P1B.**

Hydrogen bond	Donor-H [Å]	Acceptor-H [Å]	Donor-Acceptor [Å]	Angle [°]	Symmetry operator
N1-H1···O2*	1.00	2.26	2.956(11)	126	-1/2+x,3/2-y,1-z
O3-H3A···F1	0.87	1.89	2.718(14)	157	
O3-H3B···O4	0.87	2.08	2.923(12)	161	
O4-H4···F4	0.84	2.38	3.130(14)	149	3/2-x,1-y,1/2+z
C3-H3C···O1*	0.99	2.42	2.854(13)	106	
C10-H10···O1*	0.95	2.46	3.059(11)	121	
C10-H10···F2	0.95	2.53	3.275(13)	136	-1/2+x,3/2-y,1-z
C12-H12···O2*	0.95	2.32	2.963(12)	124	1/2+x,3/2-y,1-z
C12-H12···O3*	0.95	2.43	3.218(13)	141	1/2+x,3/2-y,1-z
C17-H17···O2*	0.95	2.45	2.981(13)	115	3/2-x,2-y,1/2+z
C22-H22B···F4	0.99	2.54	3.369(16)	142	3/2-x,1-y,1/2+z

*Intramolecular hydrogen bond.

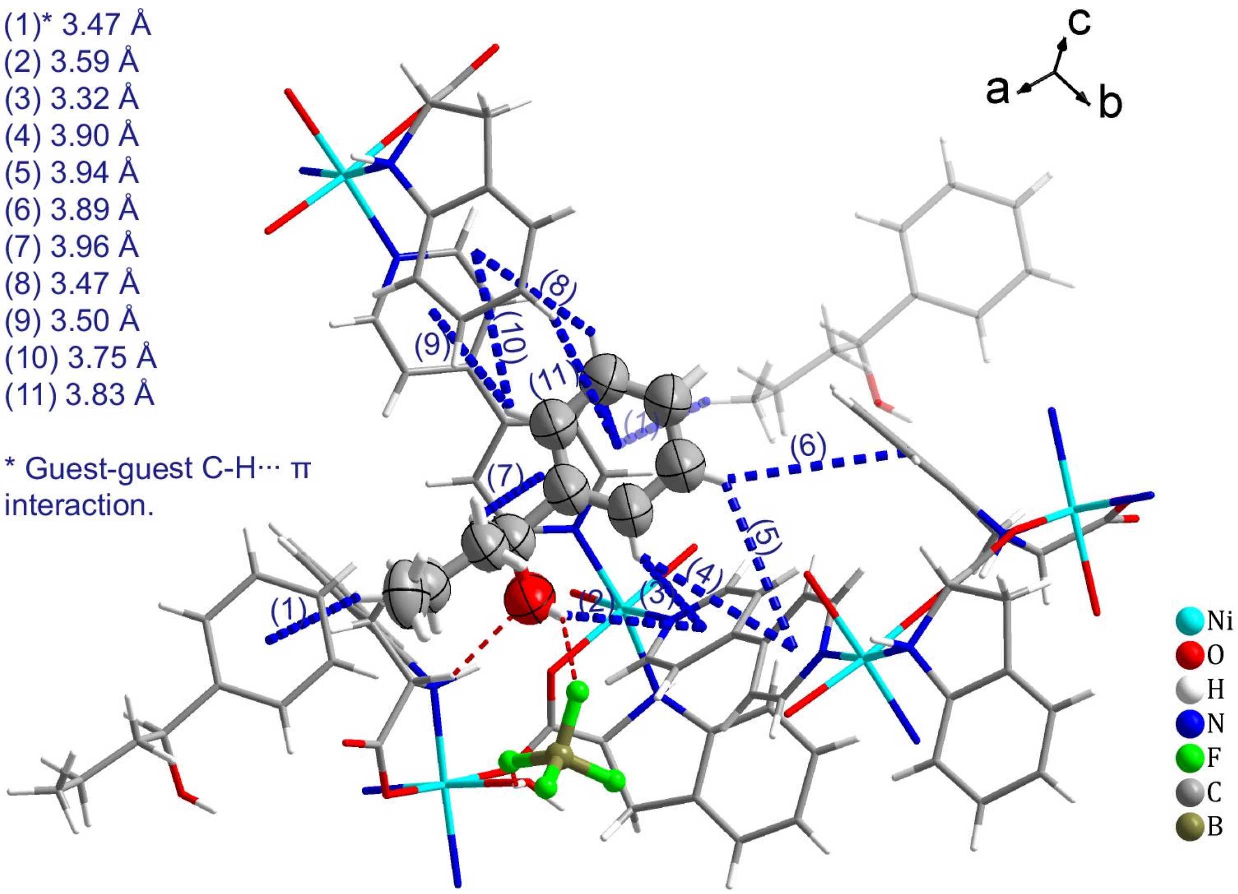

Figure S15. The C-H···π interactions (labelled by blue dash lines) between *S*-1P1B and the host framework in **CMOM-5[BF$_4$]-*S*-1P1B**.

Table S15. Hydrogen bond parameters in **CMOM-5[BF$_4$]-*S*-1P1B.**

Hydrogen bond	Donor-H [Å]	Acceptor-H [Å]	Donor-Acceptor [Å]	Angle [°]	Symmetry operator
N1-H1···O1*	1.00	2.22	2.963(9)	130	-1/2+x,3/2-y,1-z
O3-H3A···F1	0.91	1.93	2.795(10)	158	
O4-H4···F3	0.84	2.33	3.00(3)	136	
C2-H2···O4	1.00	2.32	3.16(2)	140	
C3-H3C··O2*	0.99	2.47	2.881(14)	105	
C10-H10··O2*	0.95	2.46	3.064(12)	121	-1/2+x,3/2-y,1-z
C12-H12···O1*	0.95	2.38	3.011(13)	124	
C13-H13···F2	0.95	2.45	3.399(14)	174	1/2+x,3/2-y,1-z
C17-H17···O1*	0.95	2.48	3.056(14)	119	1-x,-1/2+y,1/2-z
C18-H18···F2	0.95	2.51	3.379(16)	152	1/2+x,3/2-y,1-z
C19-H19···O2*	0.95	2.50	3.008(12)	114	3/2-x,1-y,-1/2+z

*Intramolecular hydrogen bond.

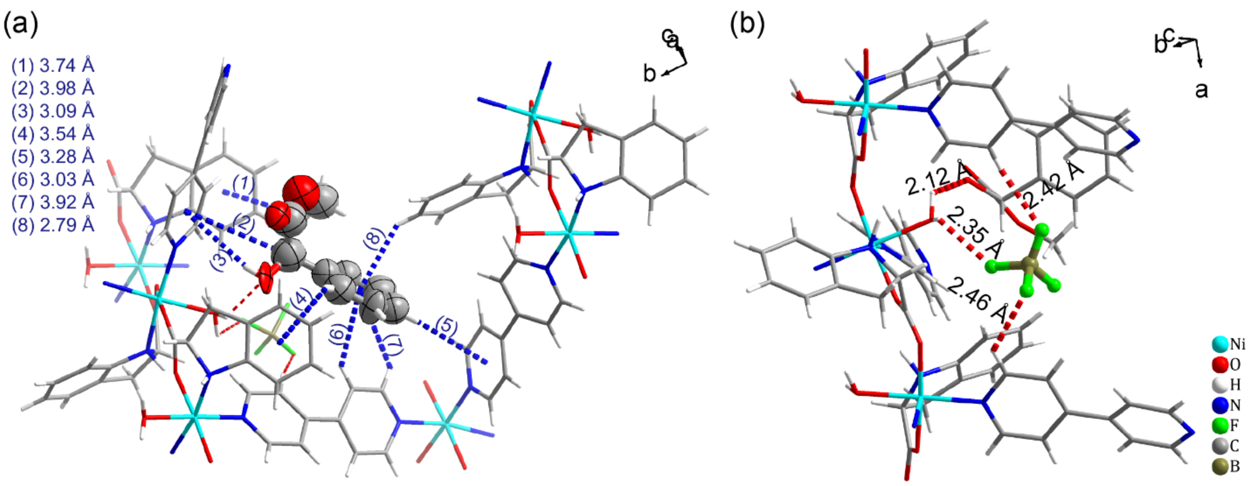

Figure S16. (a) The C-H···π interactions (labelled by blue dash lines) between *R*-MM and the host framework in **CMOM-5[BF$_4$]-*R*-MM**. (b) Intermolecular hydrogen bonds (labelled by red dash lines) around BF_4^- in **CMOM-5[BF$_4$]-*R*-MM**.

Table S16. Hydrogen bond parameters in **CMOM-5[BF$_4$]-*R*-MM.**

Hydrogen bond	Donor-H [Å]	Acceptor-H [Å]	Donor-Acceptor [Å]	Angle [°]	Symmetry operator
N1-H1···O2*	1.00	2.24	2.968(11)	129	1/2+x,3/2-y,1-z
O3-H3A···F5	0.92	2.35	2.734(15)	105	
O3-H3B···O4	0.91	2.12	2.63(2)	114	
C3-H3C···O1*	0.99	2.42	2.859(17)	106	
C12-H12···O2*	0.95	2.55	3.048(15)	113	-1/2+x,3/2-y,1-z
C16-H16··F4	0.95	2.42	3.30(2)	153	1/2-x,1-y,-1/2+z
C17-H17···O2*	0.95	2.36	2.985(14)	123	1/2-x,1-y,-1/2+z
C17-H17··O3*	0.95	2.54	3.399(15)	150	1/2-x,1-y,-1/2+z
C19-H19···F2	0.95	2.46	3.13(2)	127	-1/2-x,1-y,-1/2+z
C19-H19···O1*	0.95	2.46	3.037(14)	119	-x,-1/2+y,1/2-z

*Intramolecular hydrogen bond.

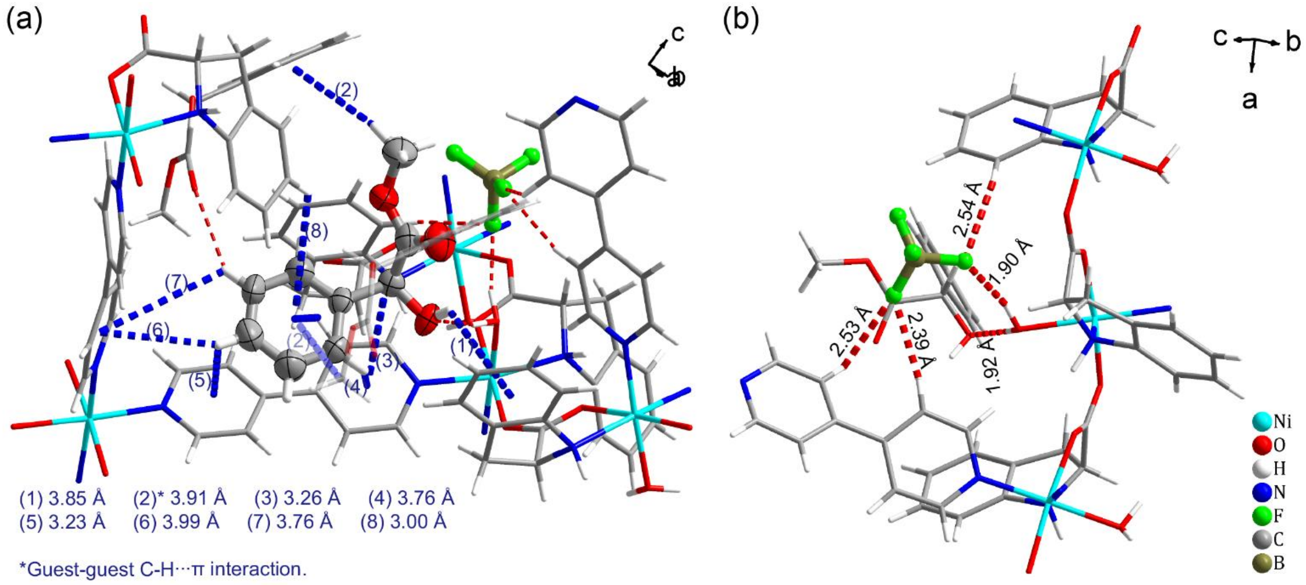

Figure S17. (a) The C-H···π interactions (labelled by blue dash lines) between *S*-MM and the host framework in **CMOM-5[BF$_4$]-*S*-MM**. (b) Intermolecular hydrogen bonds (labelled by red dash lines) around BF_4^- in **CMOM-5[BF$_4$]-*S*-MM**.

Table S17. Hydrogen bond parameters in **CMOM-5[BF$_4$]-*S*-MM.**

Hydrogen bond	Donor-H [Å]	Acceptor-H [Å]	Donor-Acceptor [Å]	Angle [°]	Symmetry operator
N1-H1···O1*	1.00	2.25	2.977(8)	128	-1/2+x,1/2-y,1-z
O3-H3A···F1	0.87	1.90	2.750(10)	164	
O3-H3B···O4	0.87	1.92	2.772(10)	167	
O4-H4···O5*	0.84	2.14	2.637(14)	117	
C3-H3C···O2*	0.99	2.42	2.857(10)	106	
C6-H6··F1	0.95	2.54	3.409(12)	153	-1+x,y,z
C10-H10···O2*	0.95	2.55	3.012(10)	110	
C11-H11··F4	0.95	2.53	3.113(14)	120	3/2-x,1-y,-1/2+z
C12-H12···O1*	0.95	2.51	3.012(10)	113	1/2+x,1/2-y,1-z
C16-H16··F4	0.95	2.39	3.328(12)	169	3/2-x,1-y,-1/2+z
C17-H17···O1*	0.95	2.36	3.002(11)	124	3/2-x,1-y,-1/2+z
C17-H17···O3*	0.95	2.49	3.336(11)	149	3/2-x,1-y,-1/2+z
C19-H19···O2*	0.95	2.44	3.028(10)	120	2-x,1/2+y,1/2-z
C24-H24···O6*	0.95	2.56	3.054(19)	112	
C25-H25···O5	0.95	2.41	3.24(2)	145	1/2+x,3/2-y,1-z
C28-H28···O4*	0.95	2.42	2.776(16)	102	

*Intramolecular hydrogen bond.

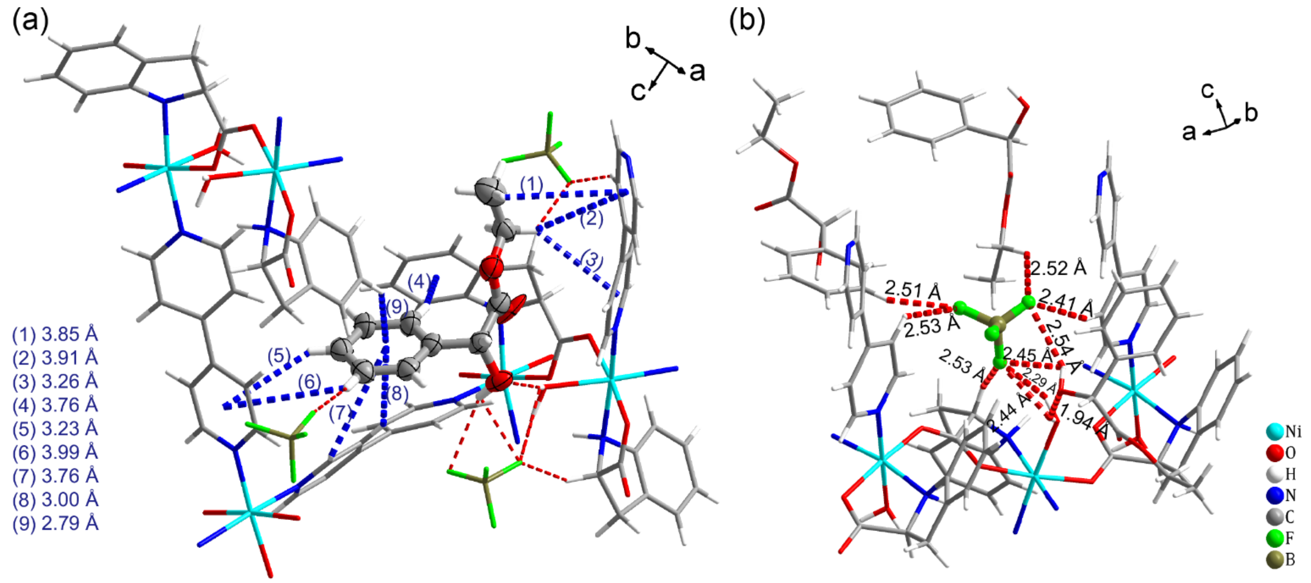

Figure S18. (a) The C-H···π interactions (labelled by blue dash lines) between *R*-EM and the host framework in **CMOM-5[BF₄]-*R*-EM**. (b) Intermolecular hydrogen bonds (labelled by red dash lines) around BF_4^- in **CMOM-5[BF₄]-*R*-EM**.

Table S18. Hydrogen bond parameters in **CMOM-5[BF₄]-*R*-EM.**

Hydrogen bond	Donor-H [Å]	Acceptor-H [Å]	Donor-Acceptor [Å]	Angle [°]	Symmetry operator
N1-H1···O1*	1.00	2.24	2.965(10)	128	1/2+x,3/2-y,1-z
O3-H3A···F1	0.94	2.44	2.844(17)	106	
O3-H3A···O4	0.94	1.94	2.78(2)	147	1/2+x,3/2-y,1-z
O3-H3B···F1	0.94	2.29	2.844(17)	117	
O4-H4···F1	0.84	2.45	2.74(2)	102	-1/2+x,3/2-y,1-z
O4-H4···F3	0.84	2.54	3.29(3)	149	-1/2+x,3/2-y,1-z
C2-H2···F1	1.00	2.53	3.263(17)	130	
C3-H3C···O2*	0.99	2.45	2.881(14)	106	
C10-H10···O2*	0.95	2.43	3.025(12)	121	1/2+x,3/2-y,1-z
C11-H11···F2	0.95	2.53	3.155(15)	124	1/2+x,3/2-y,1-z
C12-H12···O1*	0.95	2.39	3.008(13)	123	
C12-H12···O3*	0.95	2.52	3.379(13)	151	-1/2+x,3/2-y,1-z
C13-H13···F3	0.95	2.41	3.280(18)	152	-1/2+x,3/2-y,1-z
C17-H17···O1*	0.95	2.53	3.047(13)	114	1-x,1/2+y,1/2-z
C19-H19···O2*	0.95	2.56	3.035(11)	111	1/2-x,2-y,-1/2+z
C23-H23···F2	0.95	2.51	3.43(2)	163	1/2-x,2-y,-1/2+z
C28-H28B···F3	0.99	2.52	3.16(4)	122	1-x,1/2+y,3/2-z

*Intramolecular hydrogen bond.

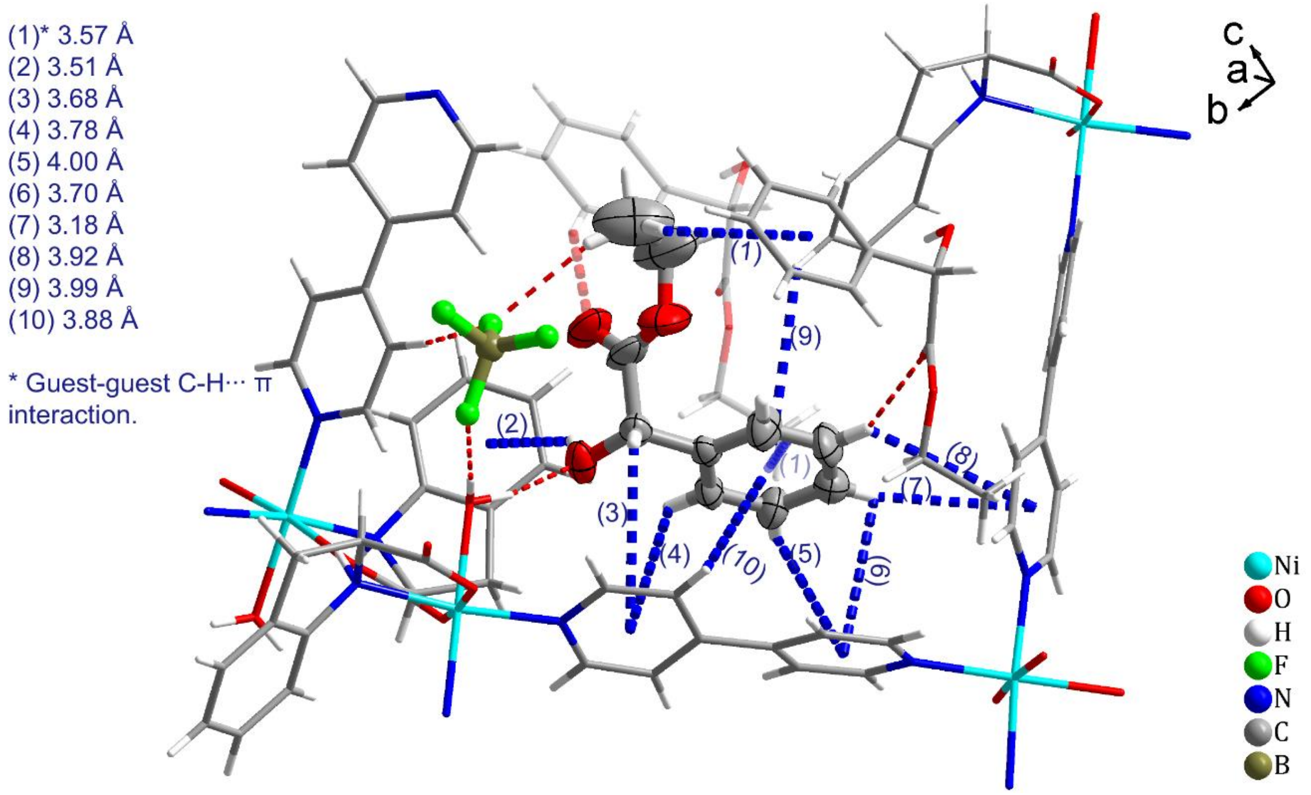

Figure S19. The C-H···π and O-H···π interactions (labelled by blue dash lines) between *S*-EM and the host framework in **CMOM-5[BF₄]-*S*-EM**.

Table S19. Hydrogen bond parameters in **CMOM-5[BF_4]-*S*-EM.**

**Hydrogen bond**	**Donor-H [Å]**	**Acceptor-H [Å]**	**Donor-Acceptor [Å]**	**Angle [°]**	**Symmetry operator**
N1-H1···O2*	1.00	2.25	2.983(7)	129	-1/2+x,3/2-y,1-z
O3-H3A···O6	0.87	1.92	2.763(9)	162	
O3-H3B···F2	0.87	1.94	2.796(10)	167	
O6-H6A···O4*	0.84	2.30	2.655(12)	106	
C3-H3C···O1*	0.99	2.43	2.861(10)	106	
C10-H10···O1*	0.95	2.56	3.028(9)	110	
C12-H12···O2*	0.95	2.51	3.028(9)	114	1/2+x,3/2-y,1-z
C16-H16··F4	0.95	2.31	3.243(12)	167	3/2-x,1-y,1/2+z
C17-H17···O2*	0.95	2.37	3.008(9)	124	3/2-x,1-y,1/2+z
C17-H17···O3*	0.95	2.51	3.372(9)	150	3/2-x,1-y,1/2+z
C19-H19···O1*	0.95	2.46	3.060(9)	121	2-x,-1/2+y,3/2-z
C20-H20···O6*	0.95	2.42	2.790(11)	103	
C23-H23···O4	0.95	2.46	3.315(13)	150	1/2+x,1/2-y,1-z
C24-H24···O5*	0.95	2.49	2.990(11)	113	
C28-H28A···O4	0.99	2.26	2.684(16)	104	
C29-H29A···F4	0.98	2.54	3.48(2)	162	

*Intramolecular hydrogen bond.

Thermogravimetric analysis

Thermogravimetric analysis (TGA) traces were recorded under N_2 using a TA Instruments Q50 system. Samples were loaded into aluminium sample pans and heated at 10 K min^{-1} from room temperature to 550 °C.

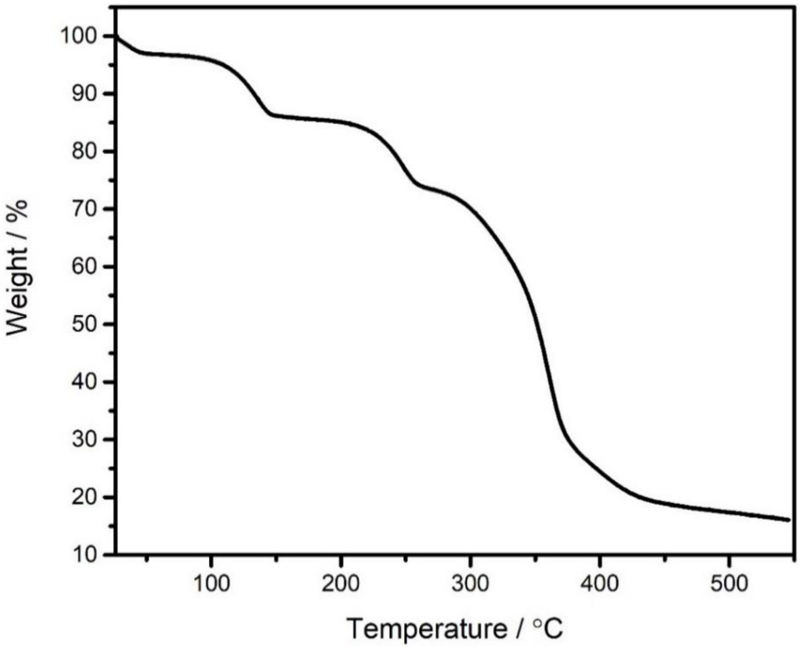

Figure S20. The TGA curves of {[Ni(*S*-IDEC)(bipy)(DMF)][BF_4](DMF)}$_n$.

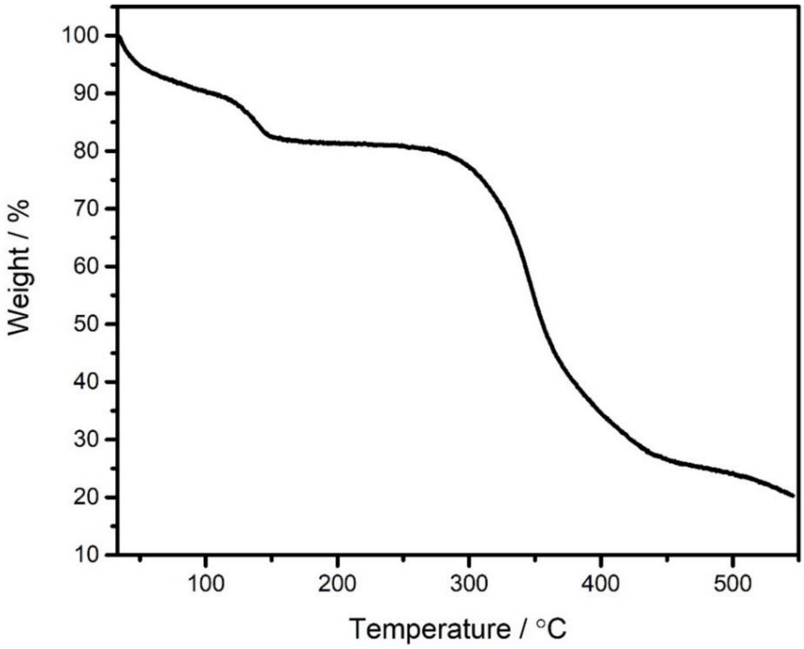

Figure S21. The TGA curves of **CMOM-5[BF_4]·EtOH**.

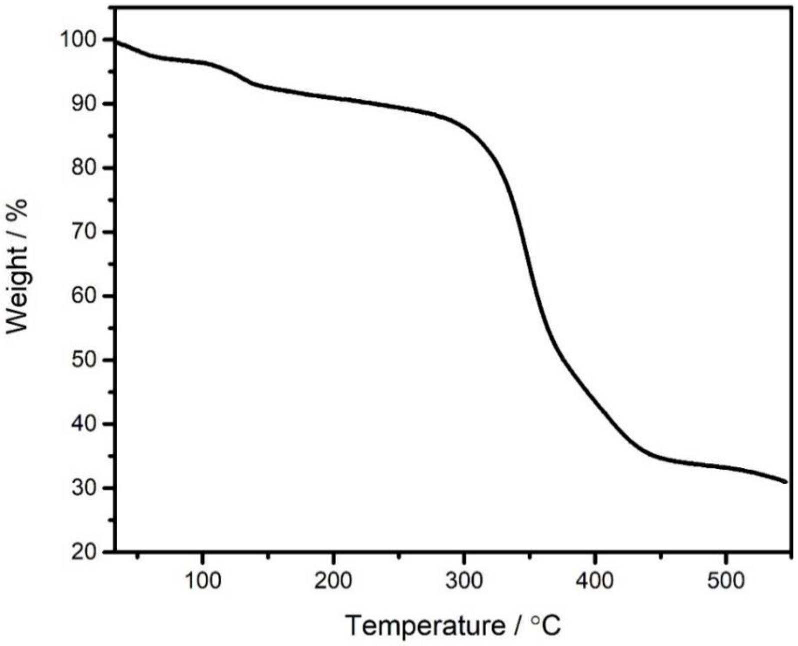

Figure S22. The TGA curves of activated **CMOM-5[BF_4]**.

Powder X-ray diffraction analysis

Powder X-ray diffraction (PXRD) data was collected at the room temperature on a PANalytical Empyream (Cu Kα, λ = 1.5418 Å) with a 1D PIXcel strip detector. Experiments were conducted in continuous scanning mode with the goniometer in the theta-theta orientation. Incident beam optics included the Fixed Divergences slit with anti-scatter slit PreFIX module, with a 1/8° divergence slit and a 1/4° anti-scatter slit, as well as a 10 mm fixed incident beam mask and a Soller slit (0.04 rad). Divergent beam optics included a P7.5 anti-scatter slit, a Soller slit (0.04 rad), and a Ni *β* filter. The data were collected in the range of 2θ = 3-50°. Raw data was then evaluated using the X'Pert HighScore Plus™ software V4.1 (PANalytical, The Netherlands).

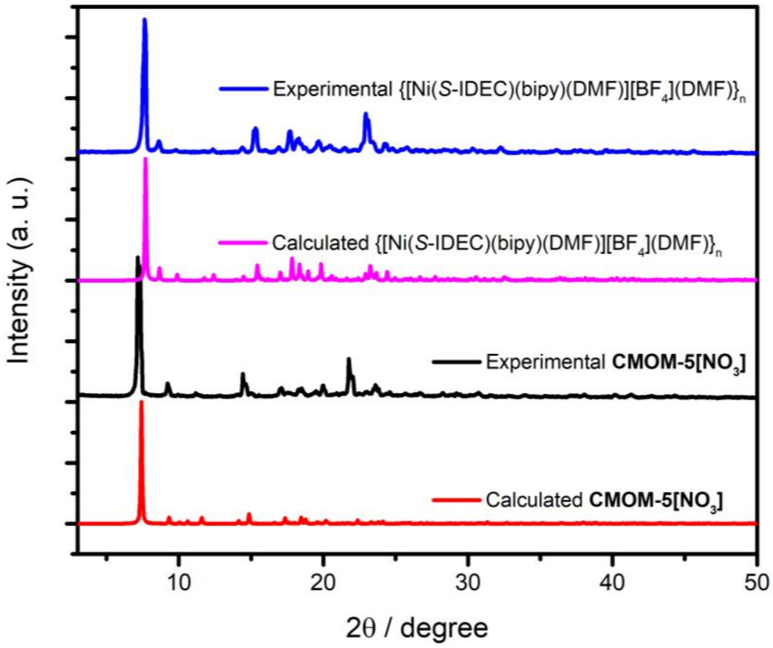

Figure S23. PXRD patterns of {[Ni(*S*-IDEC)(bipy)(DMF)][BF_4](DMF)}$_n$ and **CMOM-5[NO_3]**.

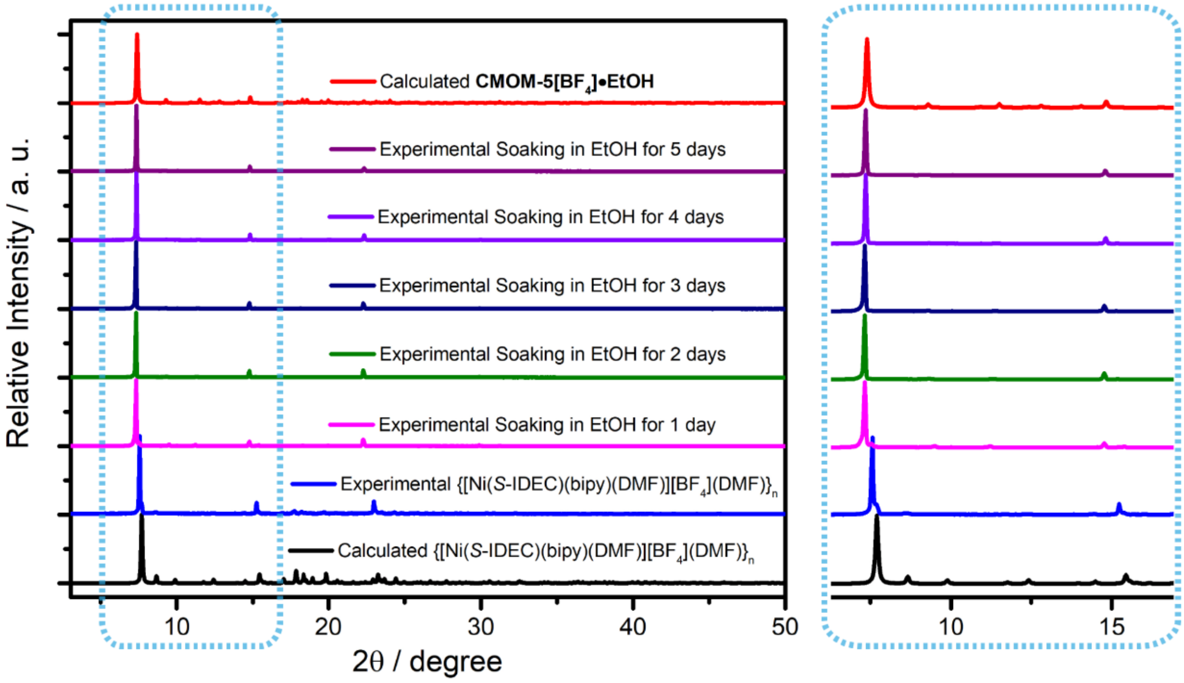

Figure S24. PXRD patterns during the transformation from {[Ni(*S*-IDEC)(bipy)(DMF)][BF_4](DMF)}$_n$ to **CMOM-5[BF_4]·EtOH** in ethanol.

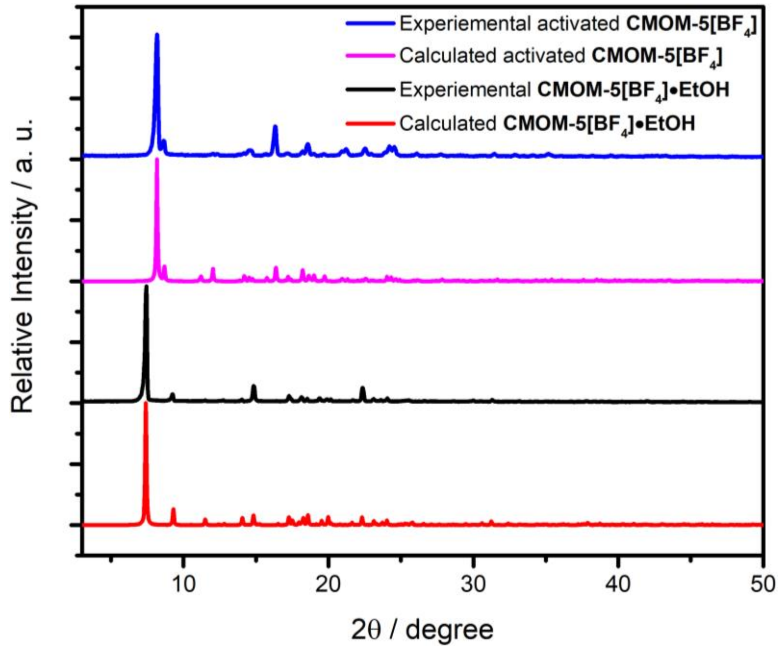

Figure S25. PXRD patterns of **CMOM-5[BF$_4$]·EtOH** and activated **CMOM-5[BF$_4$]**.

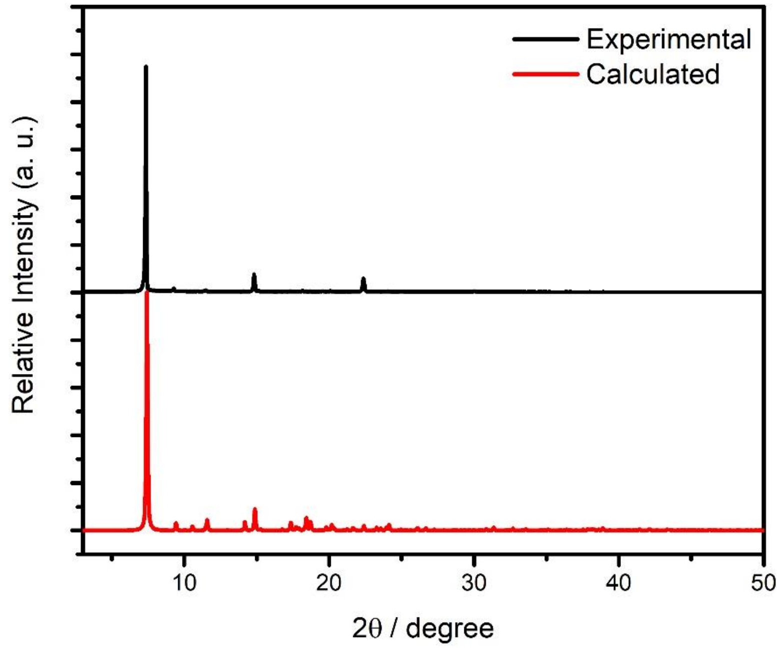

Figure S26. PXRD patterns of **CMOM-5[BF$_4$]·MeOH**.

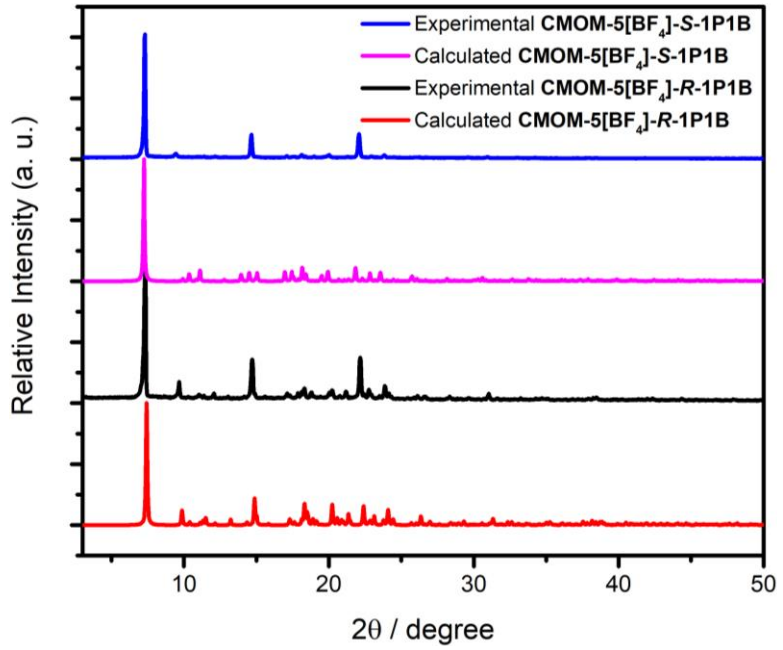

Figure S27. PXRD patterns of **CMOM-5[BF4]-*R*-1P1B** and **CMOM-5[BF4]-*S*-1P1B**.

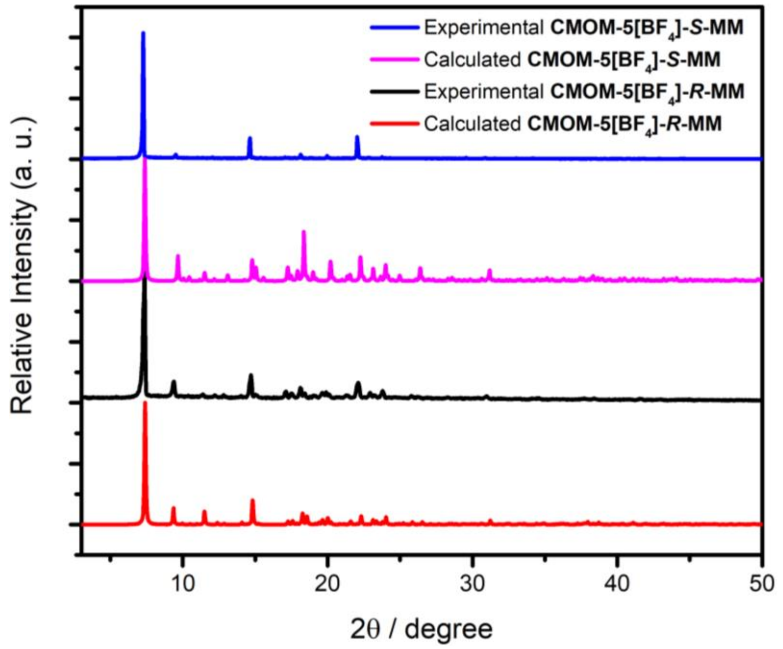

Figure S28. PXRD patterns of **CMOM-5[BF4]-*R*-MM** and **CMOM-5[BF4]-*S*-MM**.

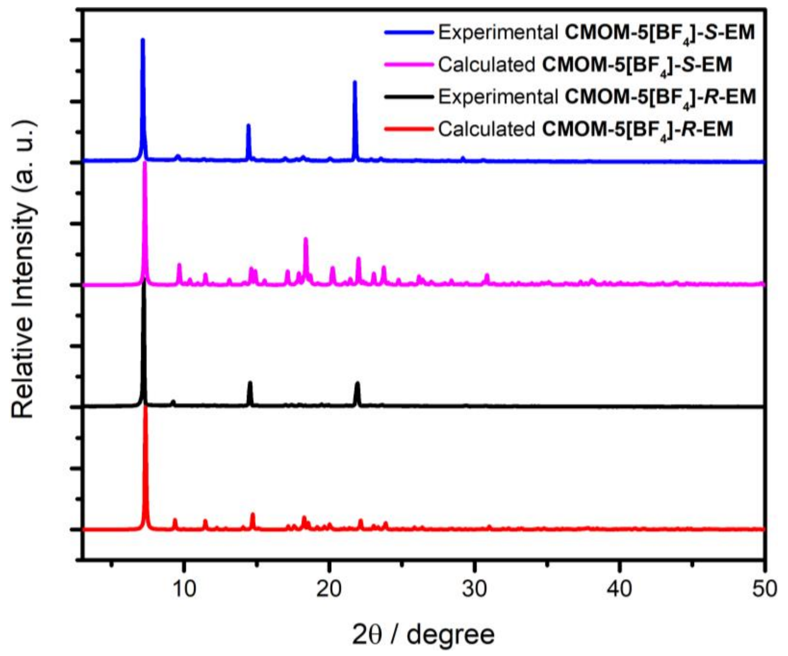

Figure S29. PXRD patterns of **CMOM-5[BF$_4$]-*R*-EM** and **CMOM-5[BF$_4$]-*S*-EM**.

Gas sorption measurements

N_2 at 77 K and CO_2 at 195 K sorption isotherms were measured using a Micromeritics Tristar II instrument. 77 K environment was controlled by a bath of liquid nitrogen. Gases were used as received from BOC Gases Ireland: N_2 (99.9995%), CO_2 (99.995%). The 195 K measurement condition was controlled by a bath of dry ice and acetone. High pressure CO_2 isotherm at 298 K was measured by a Hiden Isochema XEMIS instrument. Samples were activated under high vacuum by a Micromeritics SmartVacPrep™ system at room temperature for 12 h to remove the remnant solvent molecules prior to measurements.

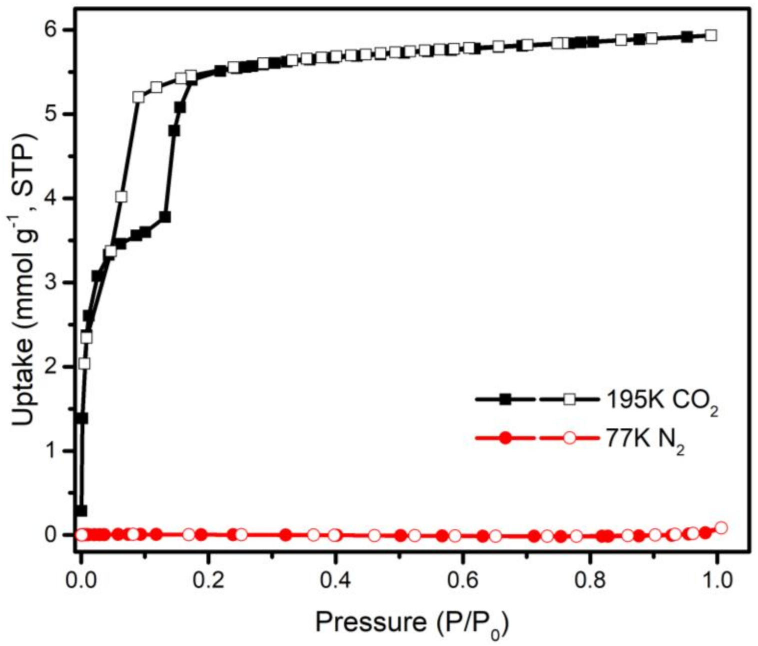

Figure S30. 195 K CO_2 and 77 K N_2 sorption isotherms of **CMOM-5[BF$_4$]**.

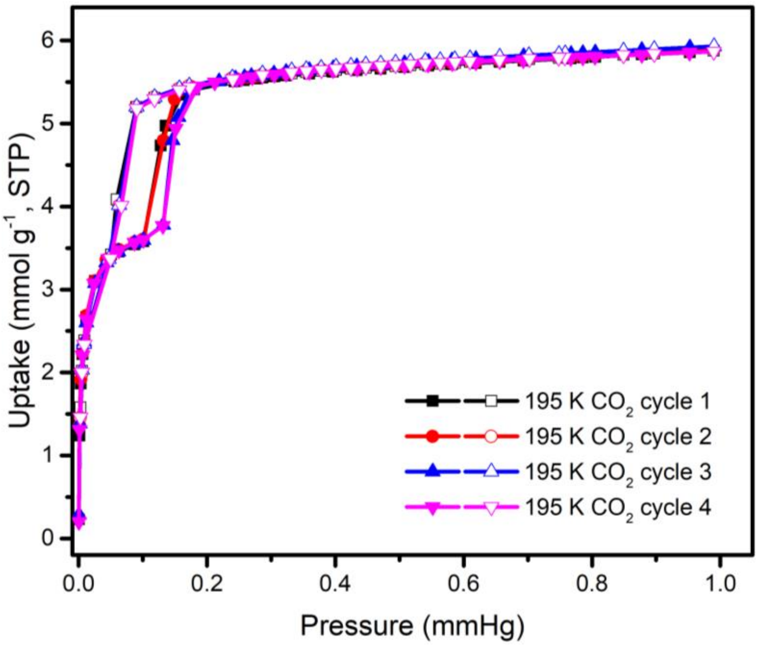

Figure S31. Four cycles of 195 K CO_2 sorption isotherms of **CMOM-5[BF$_4$]**.

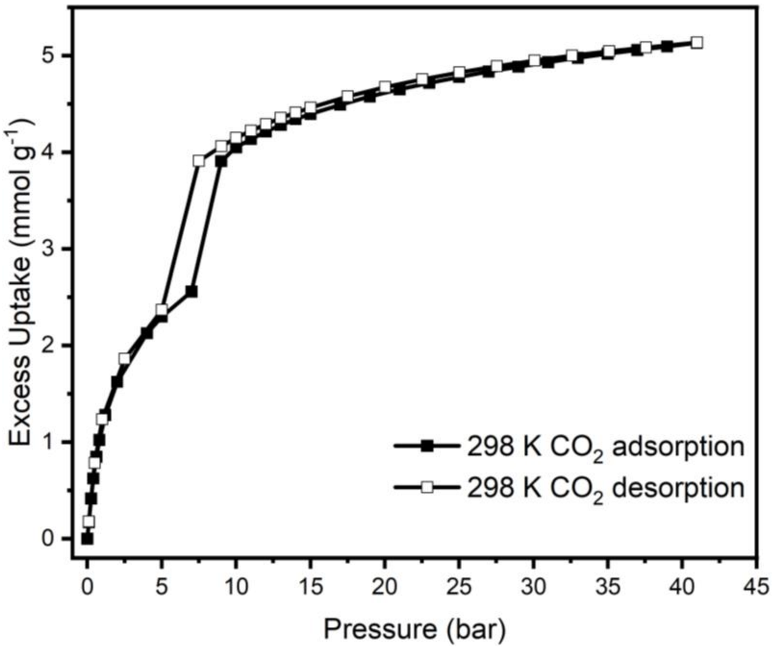

Figure S32. High-pressure 298 K CO_2 sorption isotherm of **CMOM-5[BF$_4$]**.

Calculation of the Langmuir surface area

The Langmuir isotherm model can be expressed by the following equation:[17-19]

$$\frac{Q}{Q_0} = \frac{bp}{1+bp}$$

Where Q (cm^{-3} g^{-1}) is the amount adsorbed; Q_0 (cm^{-3} g^{-1}) is the saturated amount adsorbed; *p* (mmHg) is the equilibrium pressure; and *b* ($mmHg^{-1}$) is the adsorption affinity.

A line equation for the Langmuir equation can be written as following:

$$\frac{p}{Q} = \frac{1}{bQ_0} + \frac{1}{Q_0} p$$

A least-squares fitting is performed on the ($\frac{p}{Q}, p$) designated pairs where $\frac{p}{Q}$ is the independent variable and *p* is the dependent variable (Figure. S29). The following are calculated: a) Slope ($\frac{1}{Q_0}$, g cm^{-3}, STP); b) Y-intercept ($\frac{1}{bQ_0}$, g mmHg cm^{-3}, STP); c) Error of the slope (g cm^{-3}, STP); d) Error of the y-intercept (g mmHg cm^{-3}, STP).

Using the results of the above calculations, the Langmuir surface area can be calculated as following:

$$S_{Lan} = A_m \times N_A \times \frac{Q_0}{V_{mol}}$$

Where S_{Lan} is the Langmuir surface area (m^2 g^{-1}); A_m is the molecular cross-sectional area (nm^2, 1 nm^2 = 1 × 10^{-18} m^2) of adsorbate (i.e. 0.1700 nm^2 for CO_2); V_{mol} represents the molar volume of a gas at STP (22414 cm^3 mol^{-1}), and N_A = 6.02 × 10^{23}.

Note: The Brunauer-Emmett-Teller (BET) surface area cannot be accurately determined for **CMOM-5[BF_4]** because of the switching 195 K CO_2 adsorption isotherm.

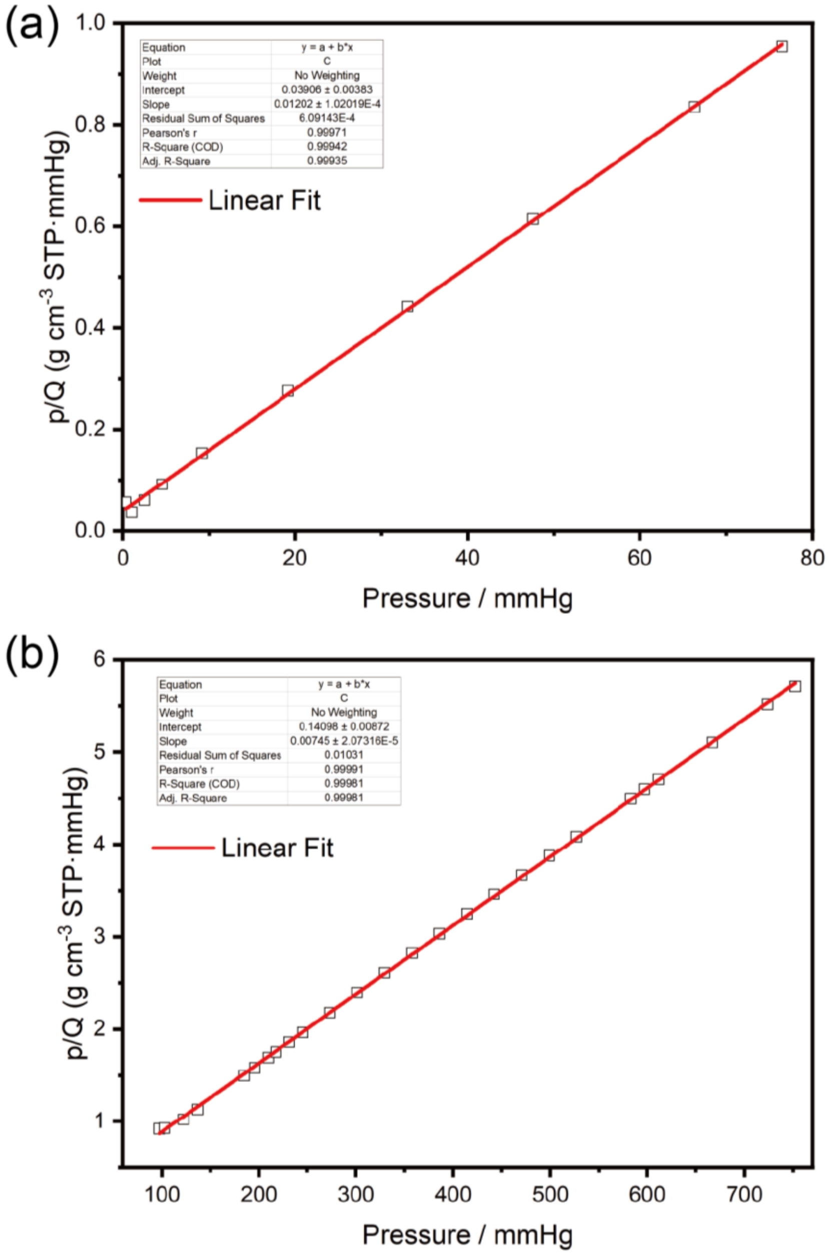

Figure S33. Langmuir fits from the 195 K CO_2 data. Squares- experimental data and line- fit for **CMOM-5[BF_4]**.

NMR spectra

The NMR spectra were recorded on a JEOL ECX400 NMR spectrometer. Crystal sample (about 4 mg) with chiral molecules loaded were fileted from the solution. When the toluene was volatilized away, dissolving the sample in a mixture of 3 mL deuterium chloride (1M solution in D_2O) and 3 mL D_6-dimethyl sulfoxide (d_6-DMSO) in an NMR tube. The chemical shift of d_6-DMSO was labelled at 2.50 ppm for all data. The multiple peaks around 3.86 ppm was attributed to deuterium chloride.[20]

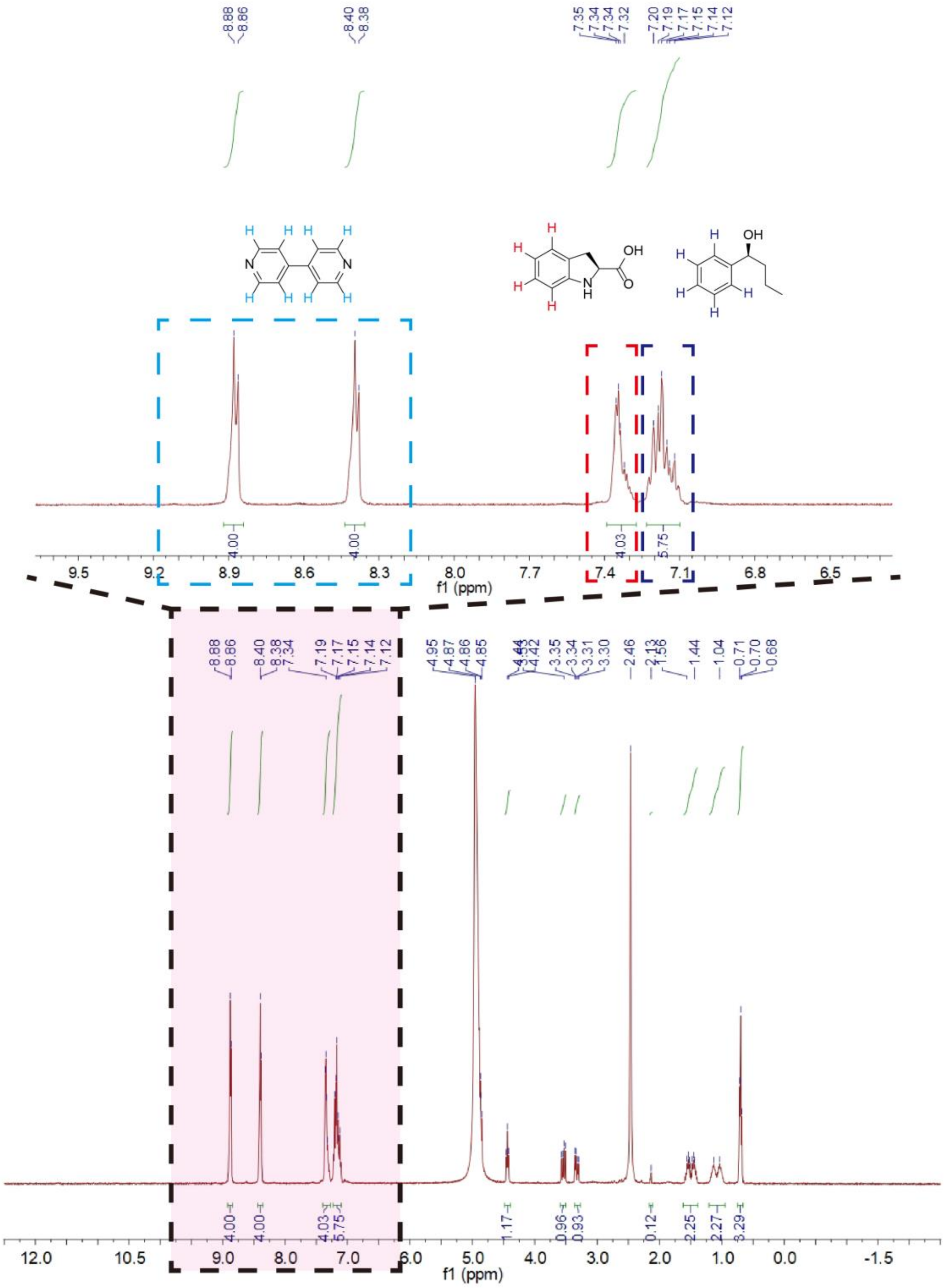

Figure S34. ^{1}H NMR spectrum of digested **CMOM-5[BF$_4$]-*R*-1P1B**. The ratio among bipy: *S*-IDEC: *R*-1P1B was solved to be 1:1:1.15.

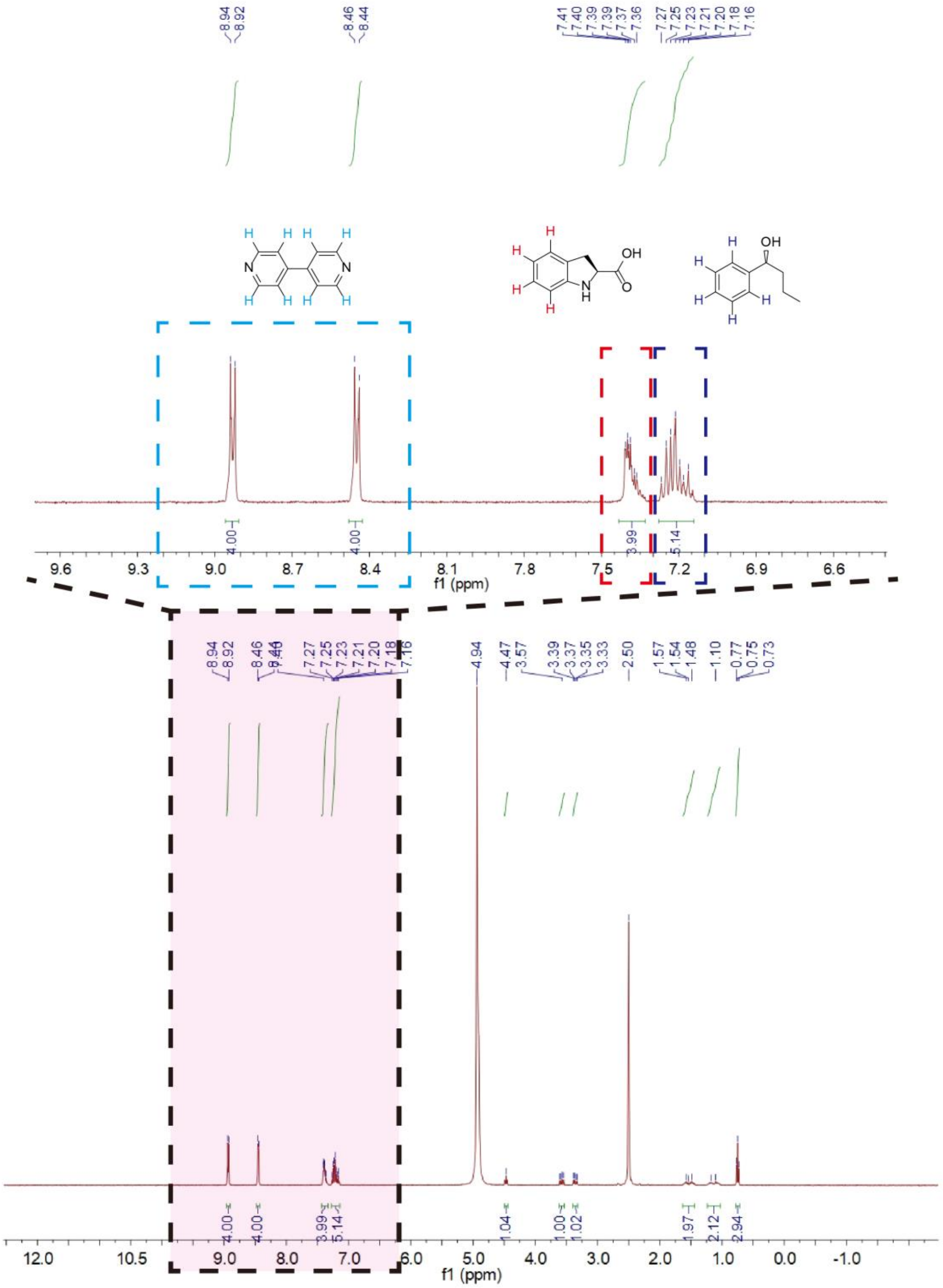

Figure S35. ^{1}H NMR spectrum of digested **CMOM-5[BF$_4$]-*S*-1P1B**. The ratio among bipy: *S*-IDEC: *S*-1P1B was solved to be 1:1:1.03.

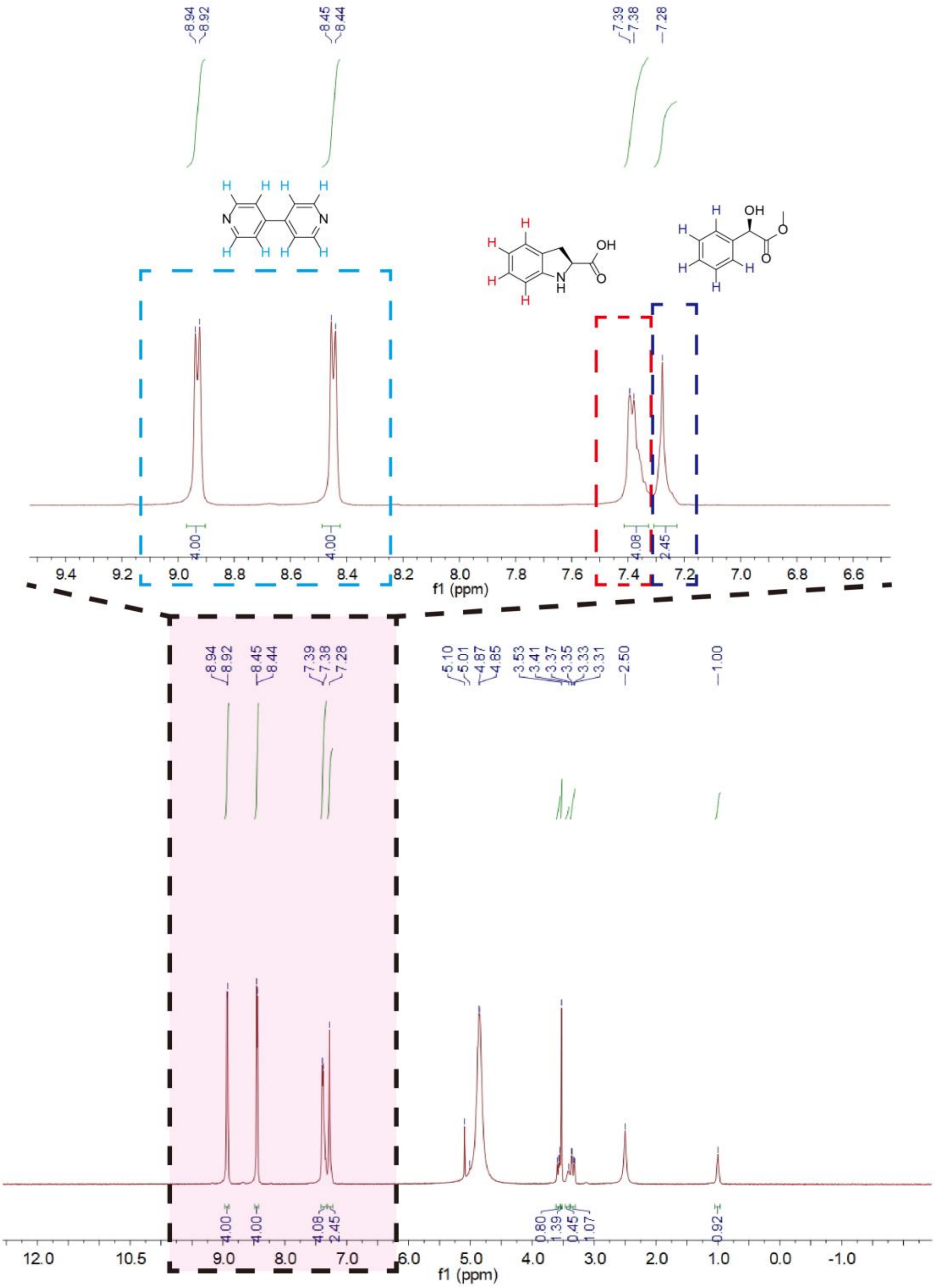

Figure S36. ^{1}H NMR spectrum of digested **CMOM-5[BF$_4$]-*R*-MM**. The ratio among bipy: *S*-IDEC: *R*-MM was solved to be 1:1:0.55.

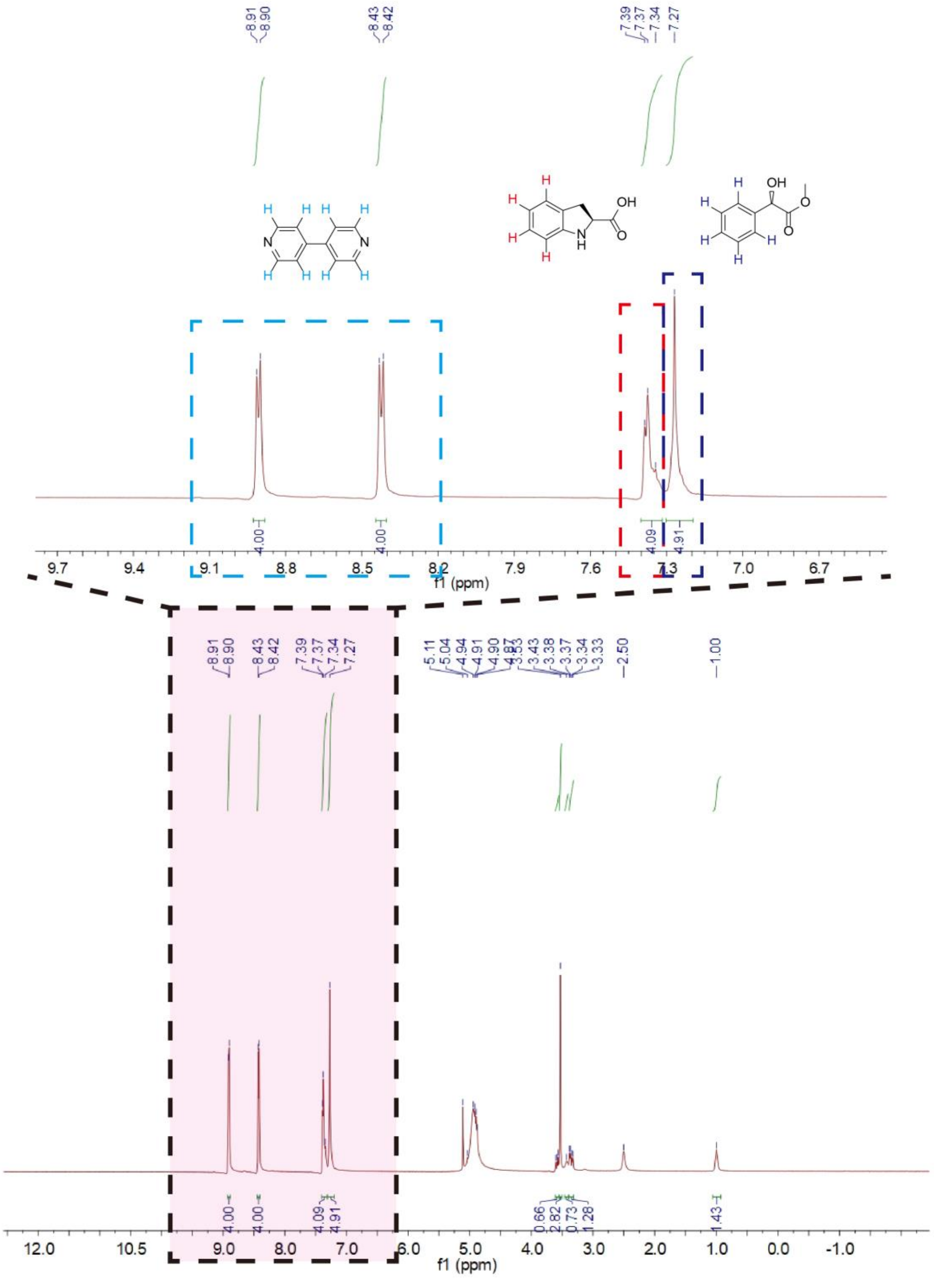

Figure S37. ^{1}H NMR spectrum of digested **CMOM-5[BF$_4$]-*S*-MM**. The ratio among bipy: *S*-IDEC: *S*-MM was solved to be 1:1.02:0.98.

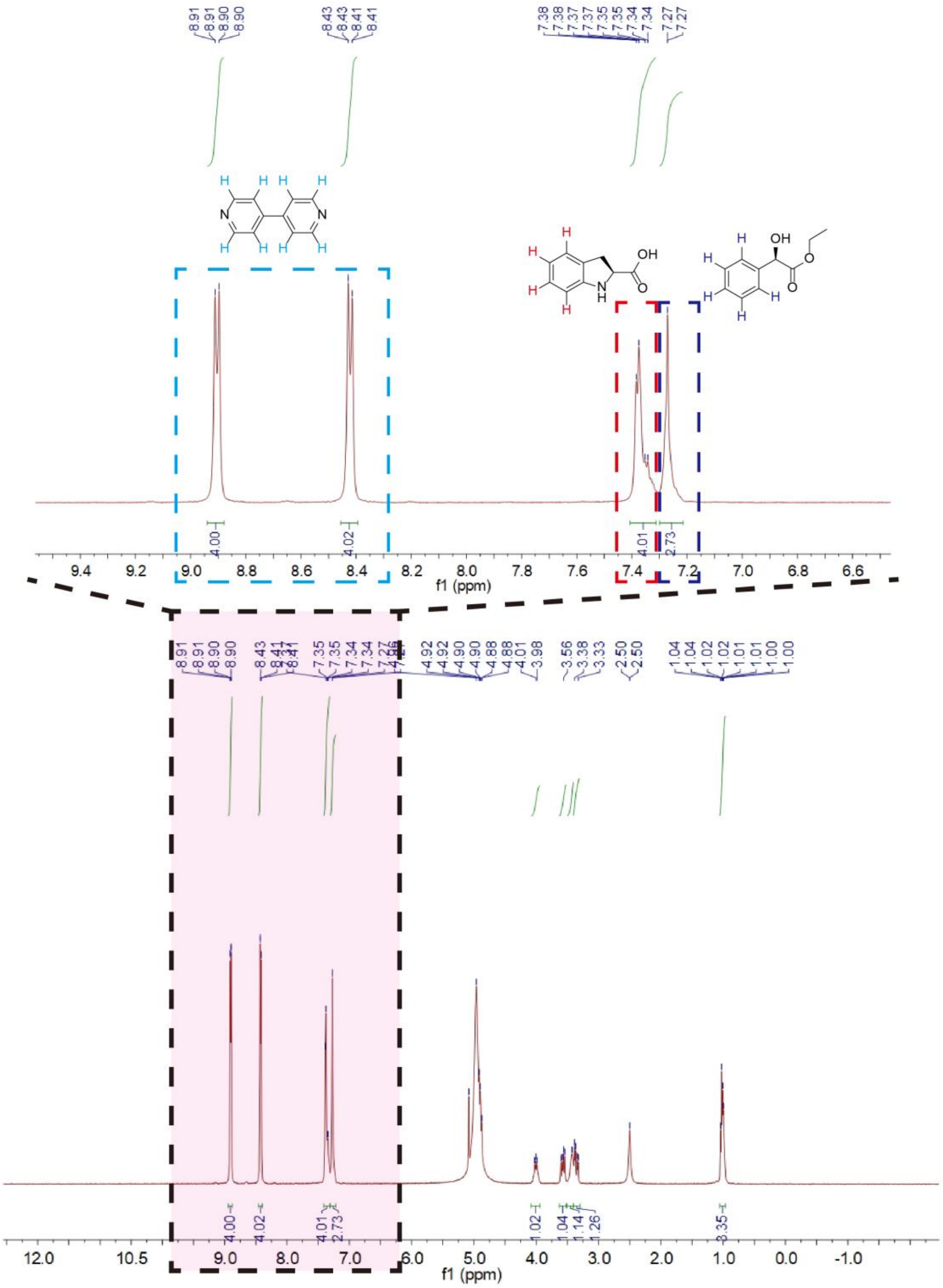

Figure S38. ^{1}H NMR spectrum of digested **CMOM-5[BF$_4$]-*R*-EM**. The ratio among bipy: *S*-IDEC: *R*-EM was solved to be 1:1:0.55.

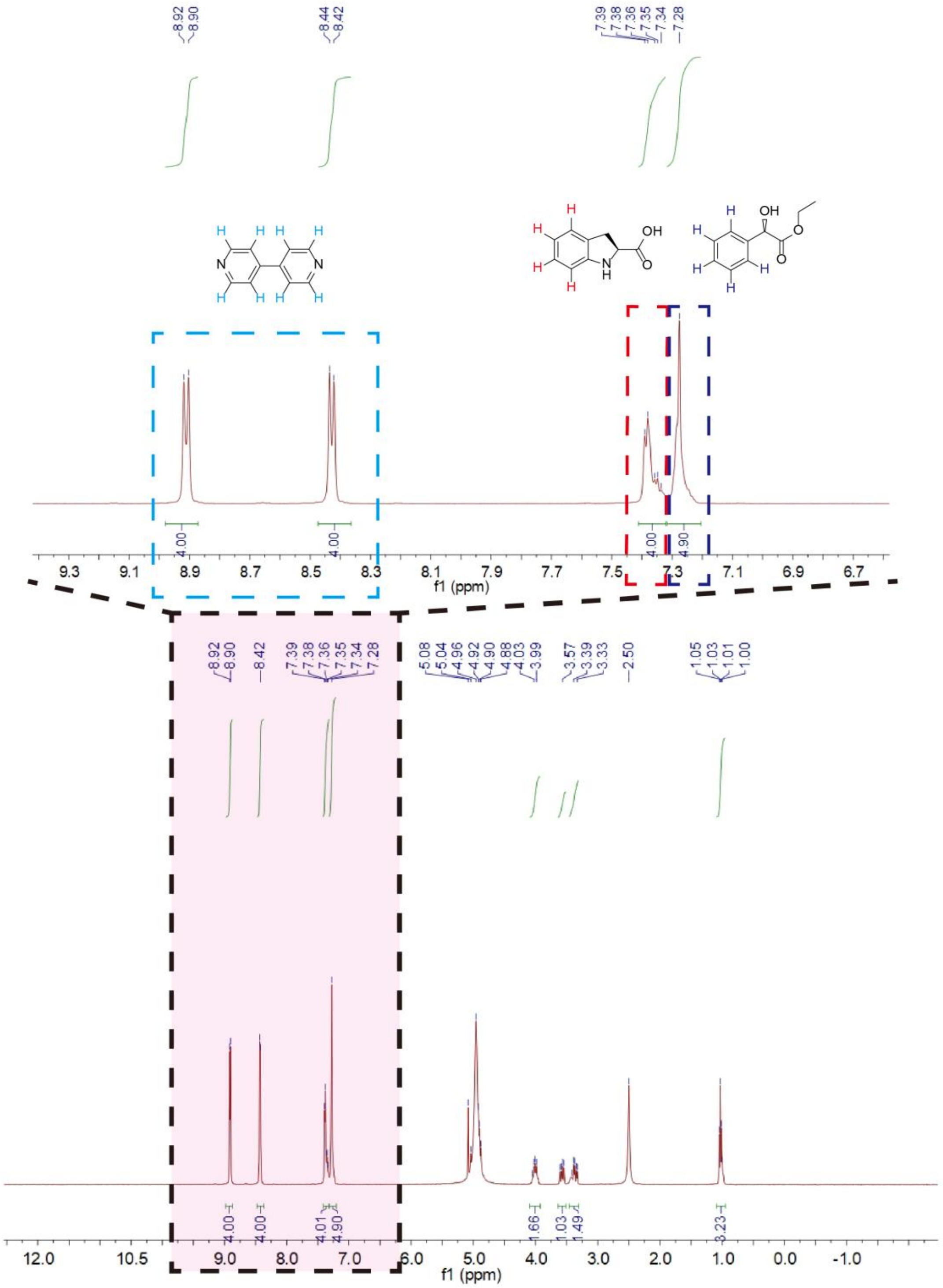

Figure S39. ^{1}H NMR spectrum of digested **CMOM-5[BF$_4$]-*S*-EM**. The ratio among bipy: *S*-IDEC: *S*-EM was solved to be 1:1:0.98.

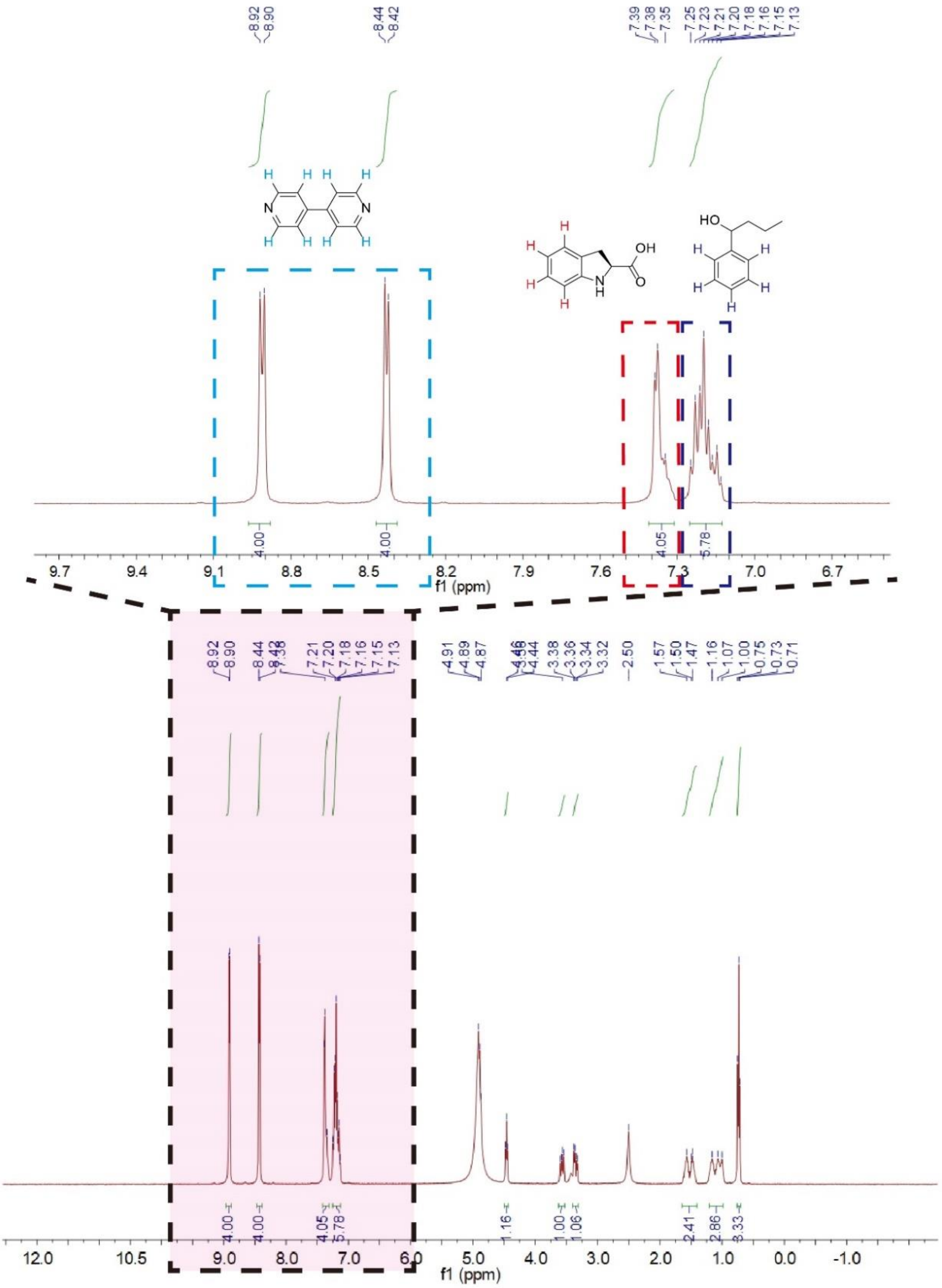

Figure S40. ^{1}H NMR spectrum of the digested **CMOM-5[BF$_4$]** collected from 1P1B chiral resolution experiment. The ratio among bipy: *S*-IDEC: 1P1B was solved to be 1:1.01:1.16.

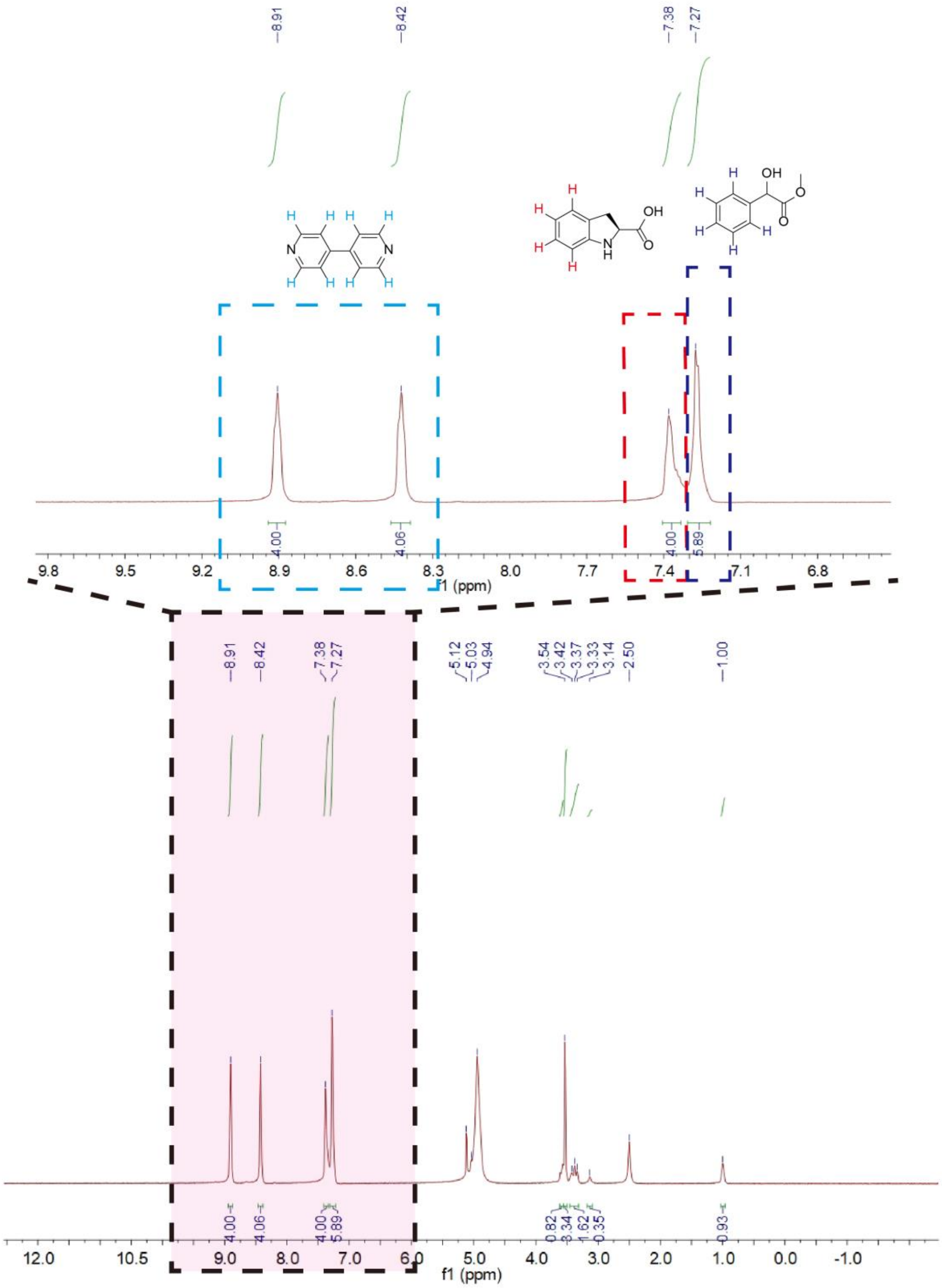

Figure S41. ^{1}H NMR spectrum of the digested **CMOM-5[BF4]** collected from MM chiral resolution experiment. The ratio among bipy: *S*-IDEC: MM was solved to be 1:1:1.18.

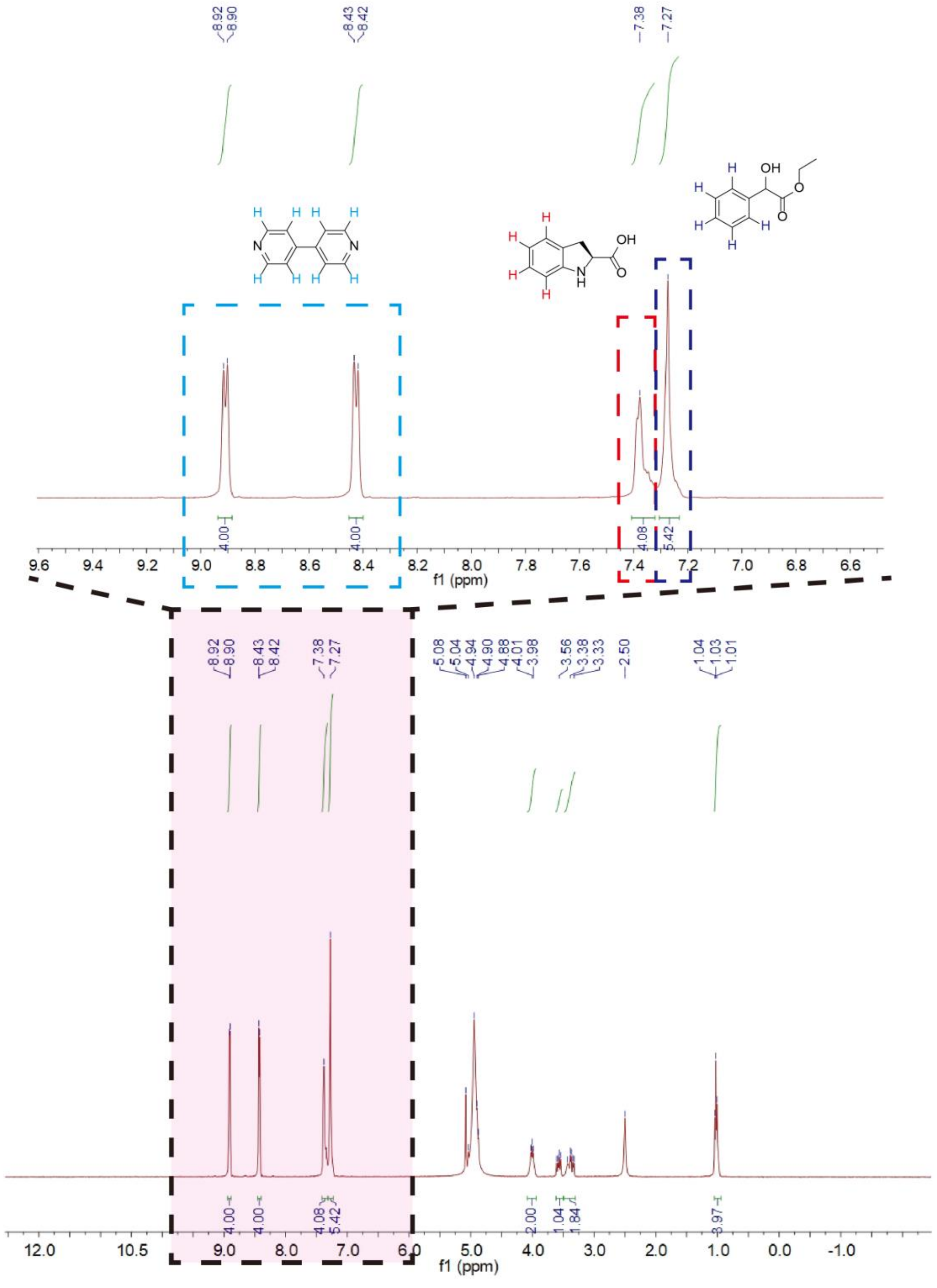

Figure S42. ^{1}H NMR spectrum of the digested **CMOM-5[BF$_4$]** collected from EM chiral resolution experiment. The ratio among bipy: *S*-IDEC: EM was solved to be 1:1.02:1.08.

HPLC methods for the analysis of samples using chiral resolution experiments

High performance liquid chromatography (HPLC) characterization for the chiral resolution experiments was done by the use of a Shimadzu HPLC system. The HPLC system was consist of LC-20AT prominence liquid chromatograph, DGU-20A5R degassing unit, SIL-20A HT prominence auto sampler, SPD-20A prominence UV/Vis detector, and CTO-20AC prominence column oven. Specific methods for the analysis of each type of chiral molecules were list as the follows.

Samples of 1P1B were analysed by the Daicel CHIRALPAK IC column (4.6 mm × 250 mm ID) at 25 °C. The eluent was n-hexane and IPA (n-hexane/IPA = 98/2). The flow rate was set at 1.0 mL/min. The wavelength of UV detector was set at 215 nm.

Samples of MM were analysed by the Daicel CHIRALPAK IB column (4.6 mm × 250 mm ID) at 25 °C. The eluent was n-hexane and IPA (n-hexane/IPA = 9/1). The flow rate was set at 0.8 mL/min constantly. The wavelength of UV detector was set at 250 nm.

Samples of EM were analysed by the Daicel CHIRALPAK IB column (4.6 mm × 250 mm ID) at 25 °C. The eluent was n-hexane and IPA (n-hexane/IPA = 9/1). The flow rate was set at 0.8 mL/min constantly. The wavelength of UV detector was set at 215 nm.

<Chromatogram>

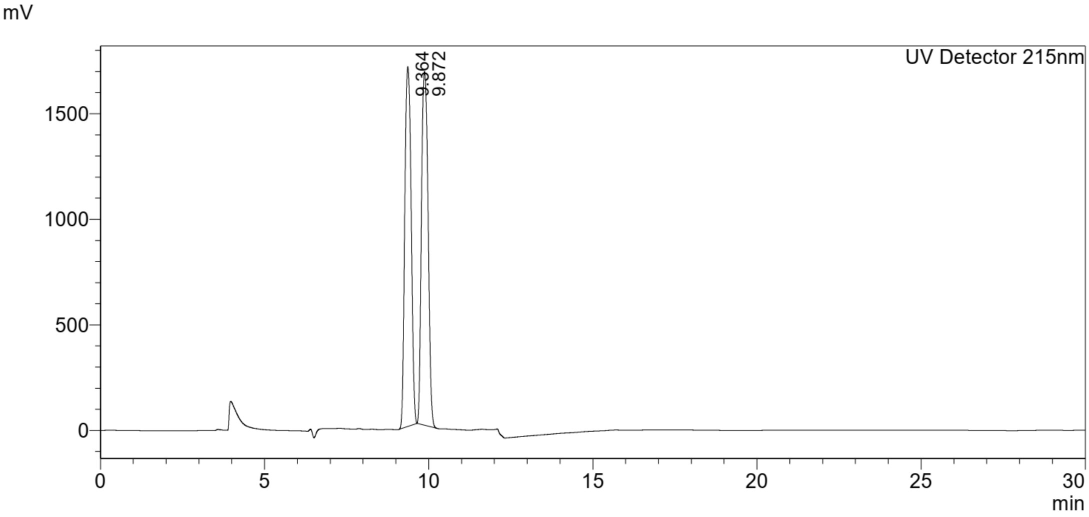

<Peak Table>

UV Detector 215nm

Peak#	Ret. Time	Area	Height	Conc.	Unit	Mark	Name
1	9.364	23089271	1703621	49.639		M	
2	9.872	23424745	1674593	50.361		M	
Total		46514016	3378214				

Figure S43. The HPLC chromatogram of racemic 1P1B.

<Chromatogram>

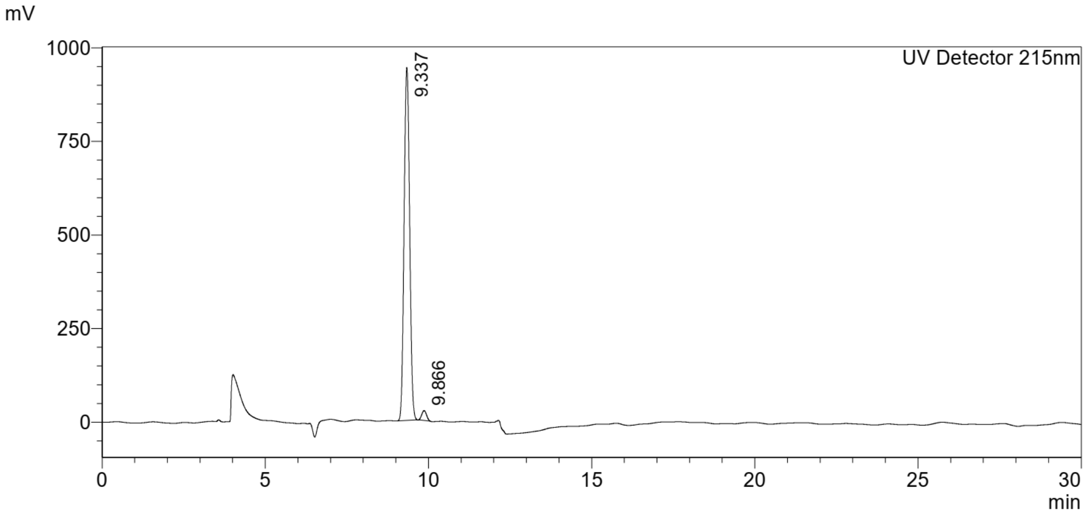

<Peak Table>

UV Detector 215nm

Peak#	Ret. Time	Area	Height	Conc.	Unit	Mark	Name
1	9.337	11133336	942018	97.640		M	
2	9.866	269081	26393	2.360		M	
Total		11402417	968411				

Figure S44. The HPLC chromatogram of *R*-1P1B.

<Chromatogram>

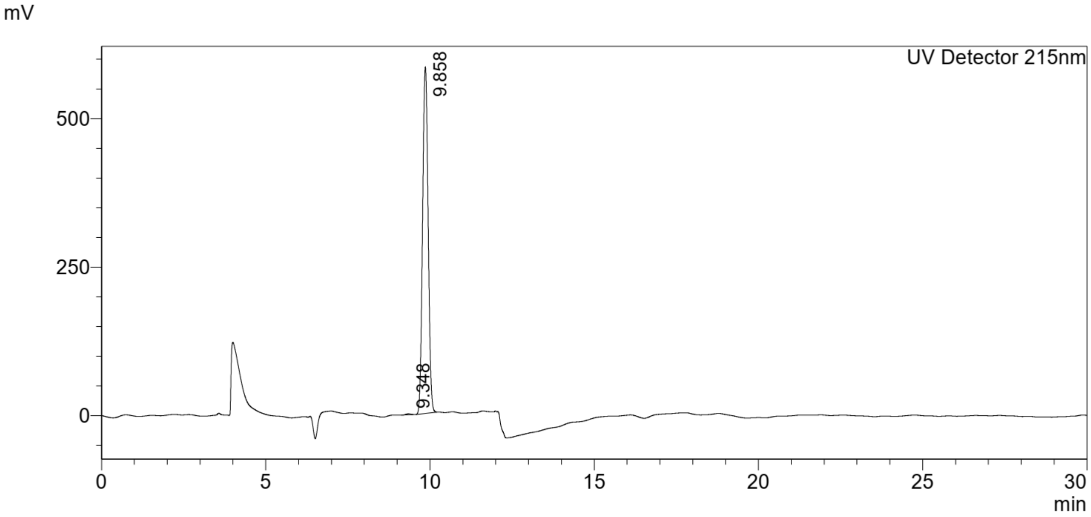

<Peak Table>

UV Detector 215nm

Peak#	Ret. Time	Area	Height	Conc.	Unit	Mark	Name
1	9.348	15594	1659	0.228		M	
2	9.858	6837558	582367	99.772		M	
Total		6853152	584027				

Figure S45. The HPLC chromatogram of *S*-1P1B.

<Chromatogram>

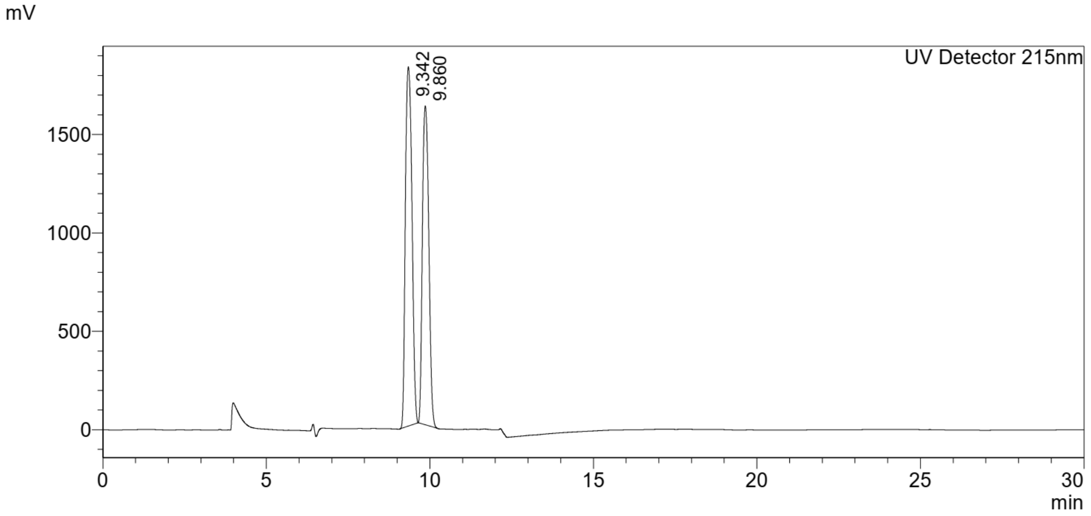

<Peak Table>

UV Detector 215nm

Peak#	Ret. Time	Area	Height	Conc.	Unit	Mark	Name
1	9.342	25544607	1823624	53.720		M	
2	9.860	22007153	1618396	46.280		M	
Total		47551759	3442020				

Figure S46. The HPLC chromatogram of the solution extracted from 1P1B encapsulated **CMOM-5[BF_4]**.

<Chromatogram>

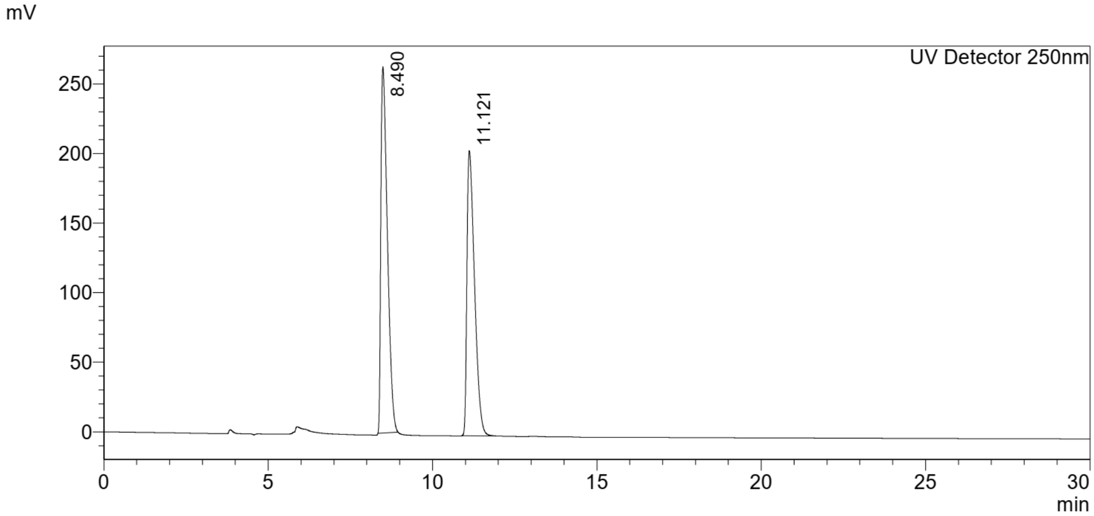

<Peak Table>

UV Detector 250nm

Peak#	Ret. Time	Area	Height	Conc.	Unit	Mark	Name
1	8.490	3634948	263064	52.490		M	
2	11.121	3290115	204886	47.510		M	
Total		6925064	467950				

Figure S47. The HPLC chromatogram of racemic MM.

<Chromatogram>

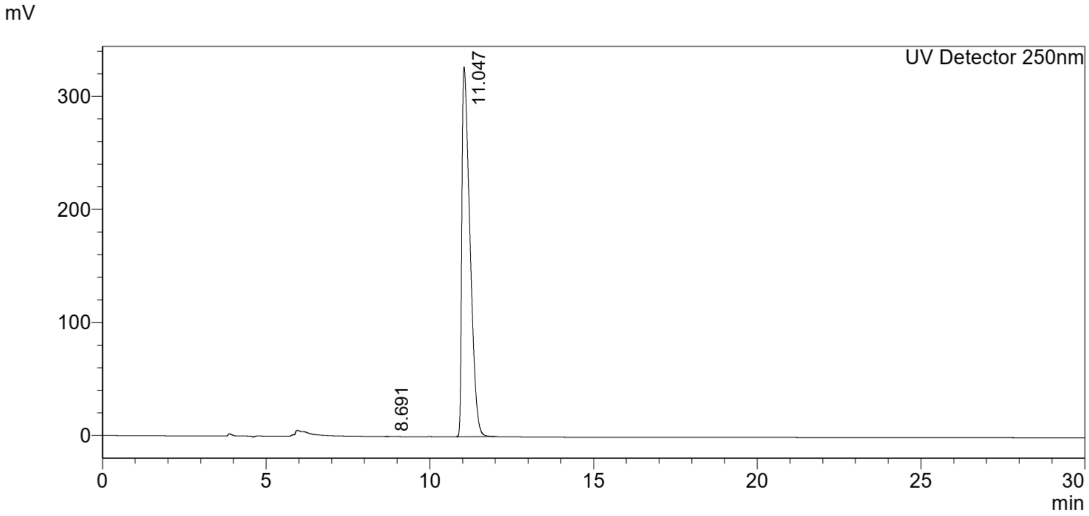

<Peak Table>

UV Detector 250nm

Peak#	Ret. Time	Area	Height	Conc.	Unit	Mark	Name
1	8.691	50	10	0.001		M	
2	11.047	5636258	326951	99.999		M	
Total		5636308	326961				

Figure S48. The HPLC chromatogram of *R*-MM.

<Chromatogram>

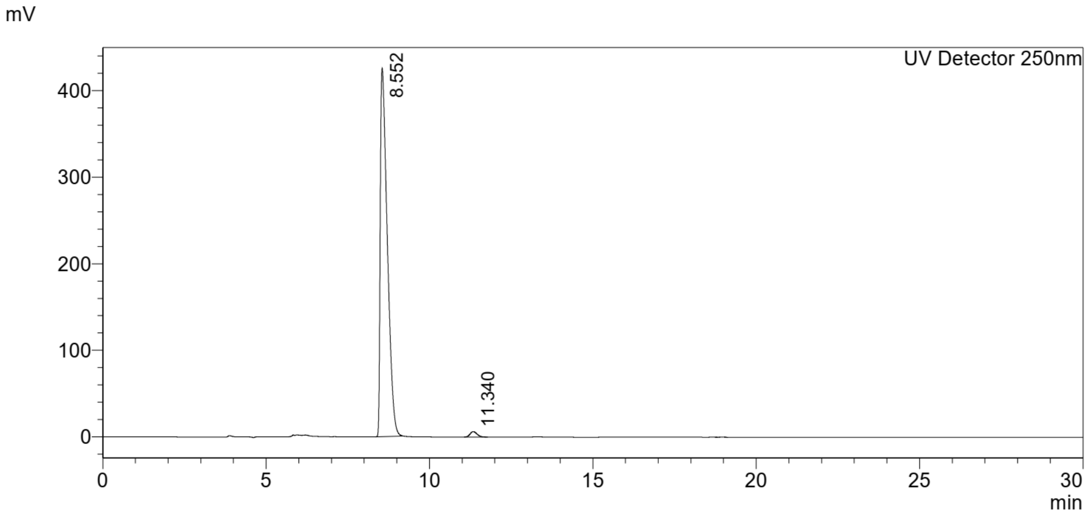

<Peak Table>

UV Detector 250nm

Peak#	Ret. Time	Area	Height	Conc.	Unit	Mark	Name
1	8.552	6390498	425073	98.578		M	
2	11.340	92167	6282	1.422		M	
Total		6482664	431356				

Figure S49. The HPLC chromatogram of *S*-MM.

<Chromatogram>

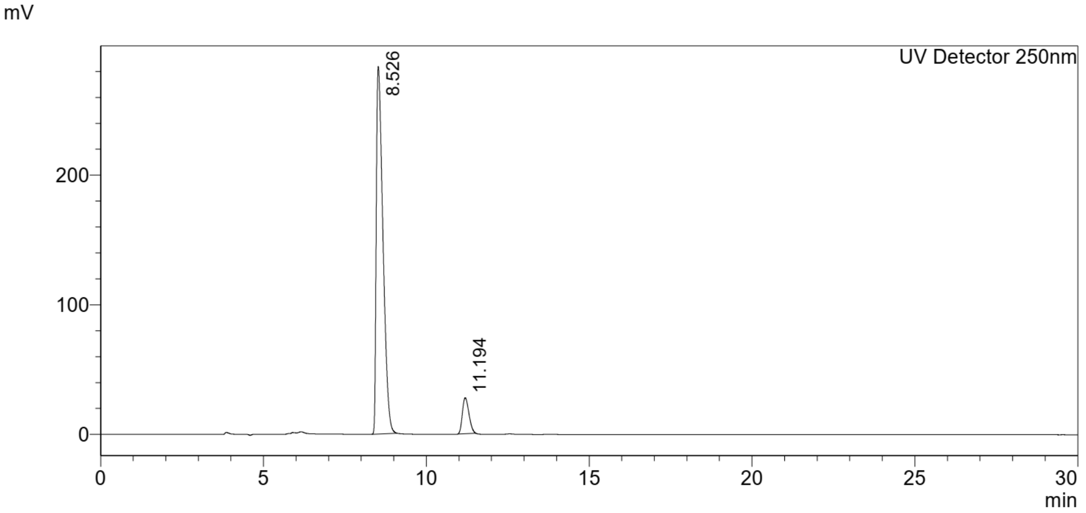

<Peak Table>

UV Detector 250nm

Peak#	Ret. Time	Area	Height	Conc.	Unit	Mark	Name
1	8.526	4046709	283130	91.275		M	
2	11.194	386820	27715	8.725		M	
Total		4433529	310845				

Figure S50. The HPLC chromatogram of the solution extracted from MM encapsulated **CMOM-5[BF$_4$]**.

<Chromatogram>

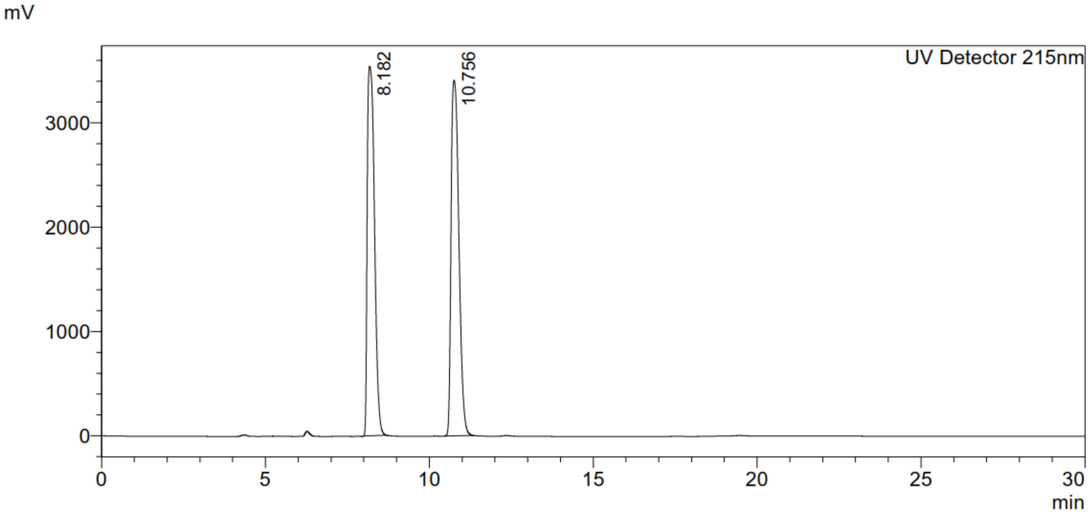

<Peak Table>

UV Detector 215nm

Peak#	Ret. Time	Area	Height	Conc.	Unit	Mark	Name
1	8.182	54129721	3540495	48.963		M	
2	10.756	56421971	3408869	51.037		M	
Total		110551693	6949364				

Figure S51. The HPLC chromatogram of racemic EM.

<Chromatogram>

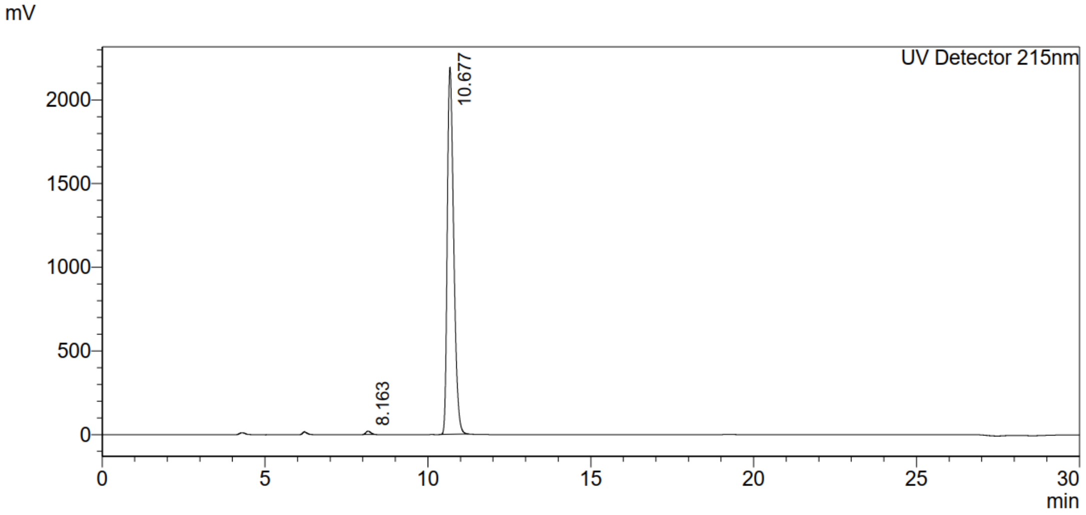

<Peak Table>

UV Detector 215nm

Peak#	Ret. Time	Area	Height	Conc.	Unit	Mark	Name
1	8.163	205452	20180	0.680		M	
2	10.677	29993781	2191301	99.320		M	
Total		30199233	2211481				

Figure S52. The HPLC chromatogram of *R*-EM.

<Chromatogram>

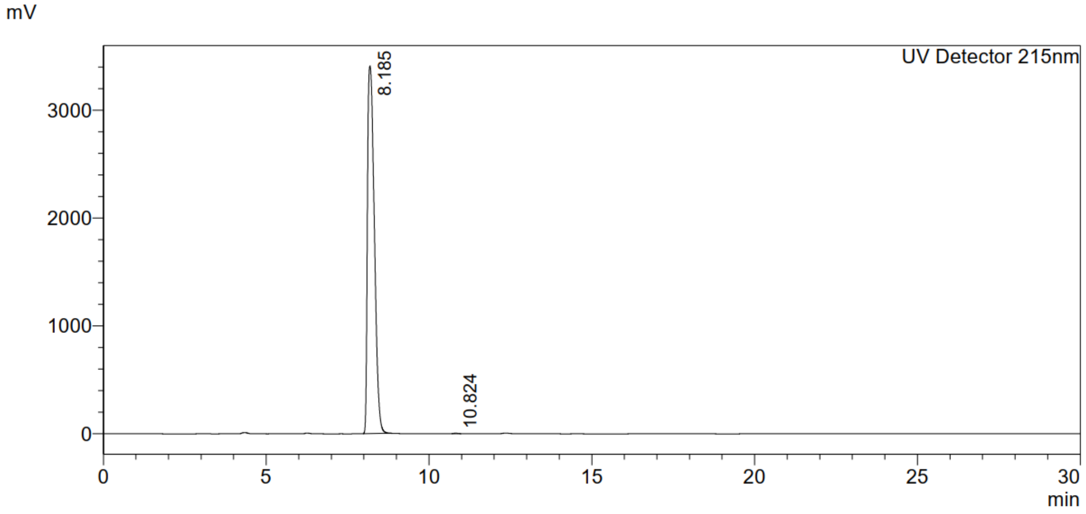

<Peak Table>

UV Detector 215nm

Peak#	Ret. Time	Area	Height	Conc.	Unit	Mark	Name
1	8.185	49830563	3405671	99.934		M	
2	10.824	33015	3475	0.066		M	
Total		49863578	3409146				

Figure S53. The HPLC chromatogram of *S*-EM.

<Chromatogram>

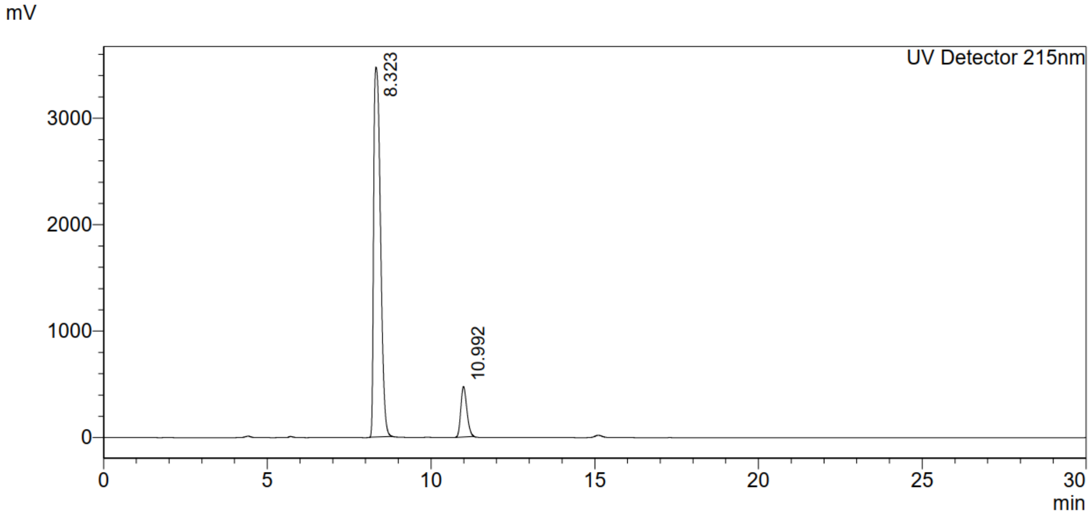

<Peak Table>

UV Detector 215nm

Peak#	Ret. Time	Area	Height	Conc.	Unit	Mark	Name
1	8.323	50257812	3473735	89.175		M	
2	10.992	6100614	474819	10.825		M	
Total		56358427	3948554				

Figure S54. The HPLC chromatogram of the solution extracted from EM encapsulated **CMOM-5[BF_4].**

<Chromatogram>

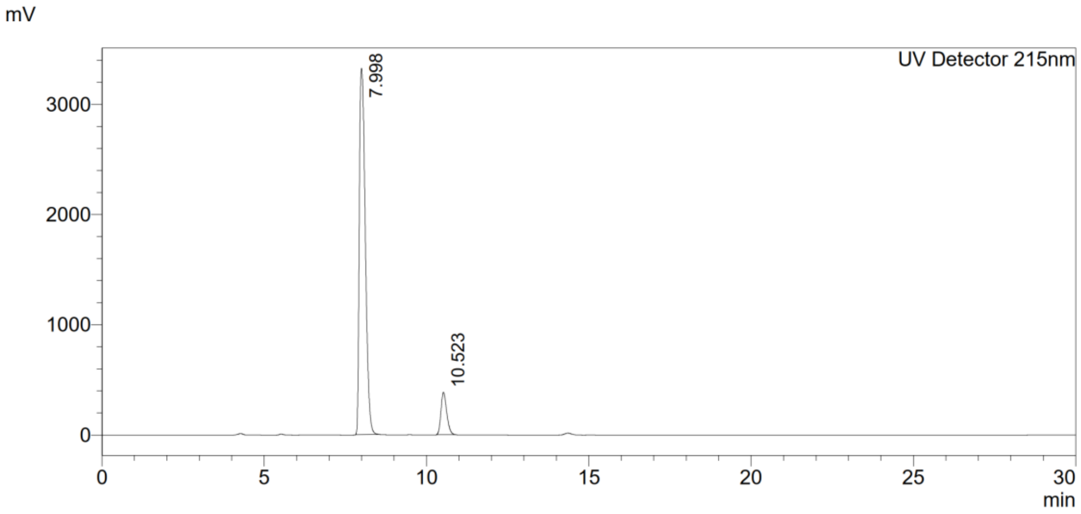

<Peak Table>

UV Detector 215nm

Peak#	Ret. Time	Area	Height	Conc.	Unit	Mark	Name
1	7.998	42487693	3321485	89.929		M	
2	10.523	4758155	384832	10.071		M	
Total		47245848	3706317				

Figure S55. The HPLC chromatogram of the solution extracted from EM encapsulated **CMOM-5[BF_4]** in the second cycle.

<Chromatogram>

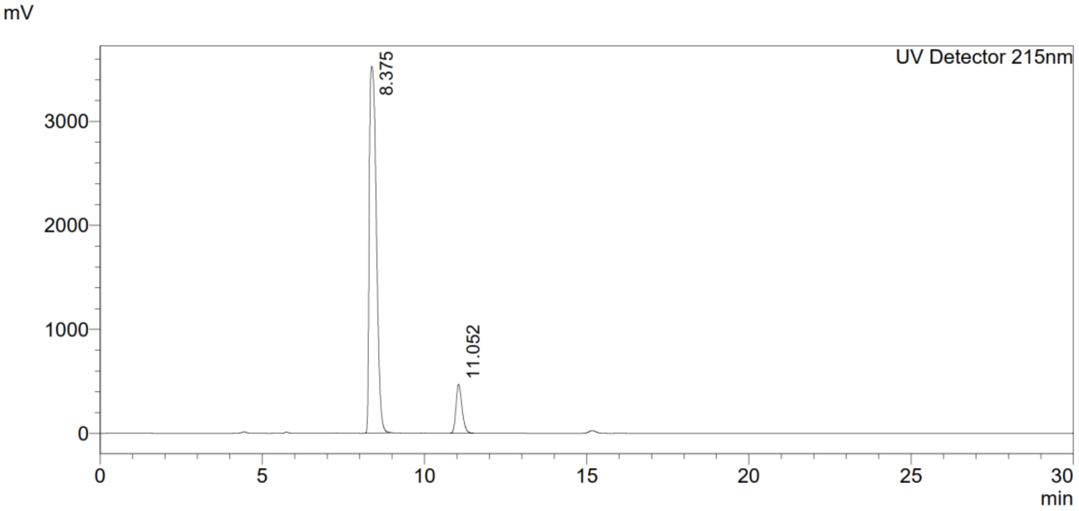

<Peak Table>

UV Detector 215nm

Peak#	Ret. Time	Area	Height	Conc.	Unit	Mark	Name
1	8.375	53072048	3525034	89.717		M	
2	11.052	6082950	469269	10.283		M	
Total		59154999	3994303				

Figure S56. The HPLC chromatogram of the solution extracted from EM encapsulated **CMOM-5[BF_4]** in the third cycle.

<Chromatogram>

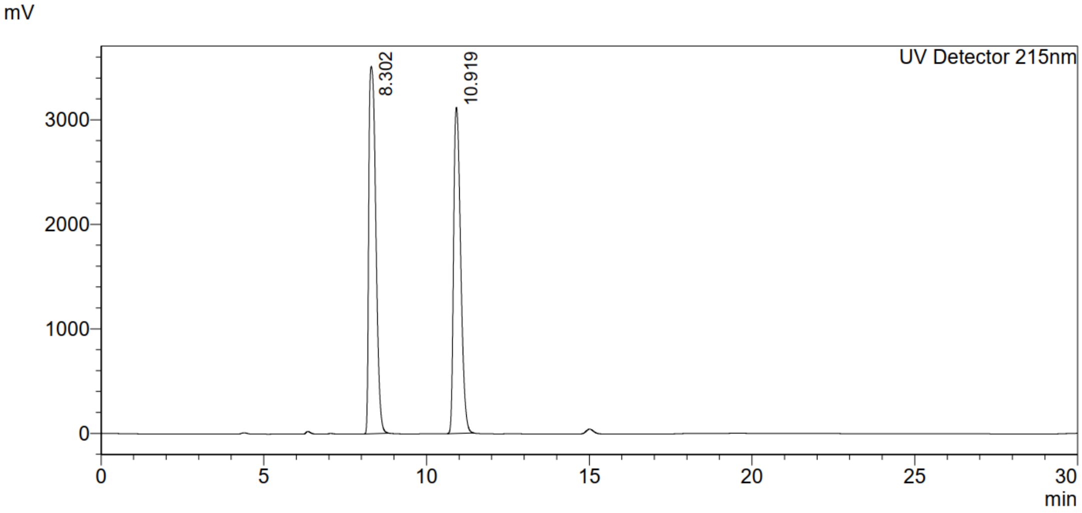

<Peak Table>

UV Detector 215nm

Peak#	Ret. Time	Area	Height	Conc.	Unit	Mark	Name
1	8.302	52893138	3512577	52.982		M	
2	10.919	46939393	3118448	47.018		M	
Total		99832531	6631025				

Figure S57. The HPLC chromatogram of the solution extracted from EM encapsulated **CMOM-5[NO_3]**.

Table S20. Chiral resolution performances of **CMOM-5[BF$_4$]** *versus* the other relevant CMOM pair.

Materials	Ligands	*ee*, %			Reference
		1P1B	MM	EM	
**CMOM-5[BF$_4$]**	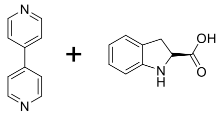	7.4 (*R* > *S*)	82.6 (*S* > *R*)	78.4 (*S* > *R*)	This work
**CMOM-5[NO$_3$]**		36.2 (*R* > *S*)	93.5 (*S* > *R*)	6.0 (*S* > *R*)	1
**[DyNaL(H$_2$O)$_4$]$_6$(H$_2$O)**	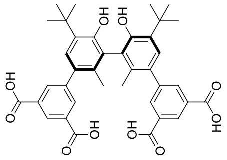	--	93.1 (*S* > *R*)	64.3 (*S* > *R*)	21

References

1. Deng, C.; Song, B.-Q.; Lusi, M.; Bezrukov; Haskins, M. M.; Gao, M.-Y.; Peng, Y.-L.; Ma, J.; Cheng, P.; Mukherjee, S.; Zaworotko, M. J. Crystal Engineering of a Chiral Crystalline Sponge That Enables Absolute Structure Determination and Enantiomeric Separation. *Cryst. Growth Des.* **2023**, *23* (7), 5211-5220. DOI: 10.1021/acs.cgd.3c00446.
2. Davankov, V. A. Analytical Chiral Separation Methods. *Pure & Appl. Chem.*, **1997**, *69* (7), 1469-1474. DOI: 10.1351/pac199769071469.
3. Groom, C. R.; Bruno, I. J.; Lightfoot, M. P.; Ward, S. C. The Cambridge Structural Database. *Acta Crystallogr., Sect. B.: Struct. Sci., Cryst. Eng. Mater.* **2016**, *B 72*, 171-179. DOI: 10.1107/S2052520616003954.
4. Zhang, S.-Y.; Yang, C.-X.; Shi, W.; Yan, X.-P.; Cheng, P.; Wojtas, L.; Zaworotko, M. J. A Chiral Metal-Organic Material that Enables Enantiomeric Identification and Purification. *Chem* 2017, 3 (2), 281-289. DOI: 10.1016/j.chempr.2017.07.004.
5. Hu, T.; Liu, T.; Zhang, Z.; Wang, Y.; Yang, Y.; Young, D. J.; Hu, C.; Lang, J.-P. Precise Control of Chirality Transfer by Adjusting the Alkyl Substituents of Guests. *Dyes Pigm.* **2018**, *160*, 692-699. DOI: 10.1016/j.dyepig.2018.09.004.
6. Akazome, M.; Takahashi, T.; Ogura, K. Enantiomeric Inclusion of *α*-Hydroxy Esters by (*R*)-(1-Naphthyl)glycyl-(*R*)-phenylglycine and the Crystal Structures of the Inclusion Cavities. *J. Org. Chem.* **1999**, *64* (7), 2293-2300. DOI: 10.1021/jo9818778.
7. Mravik, A.; Böcskei, Z.; Katona, Z.; Markovits, I.; Pokol, G.; Menyhárd D. K.; Fogassy, E. A New Optical Resolution Method: Coordinative Resolution of Mandelic Acid Esters. The Crystal Structure of Calcium Hydrogen (2*R*,3*R*)-O,O′-Dibenzoyl Tartrate–2(*R*)-(–)-Methyl Mandelate. *Chem. Commun.*, **1996**, (16), 1983-1984. DOI: 10.1039/CC9960001983.
8. Albrecht, M.; Borba, A.; Barbu-Debus, K. L.; Dittrich, B.; Fausto, R.; Grimme, S.; Mahjoub, A.; Nedić, M.; Schmitt, U.; Schrader, L.; Suhm, M. A.; Zehnacker-Rentien, A.; Zischang, J. Chirality Influence on the Aggregation of Methyl Mandelate. *New J. Chem.* **2010**, *34* (7), 1266-1285. DOI: 10.1039/c0nj00142b.
9. Tumanova, N.; Payen, R.; Springuel, G.; Norberg, B.; Robeyns, K.; Le Duff, C.; Wouters, J.; Leyssens, T. Cocrystallization Out of the Blue: DL-Mandelic Acid/Ethyl-DL-Mandelate Cocrystal. *J. Mol. Struct.* **2017**, *1127*, 397-402. DOI: 10.1016/j.molstruc.2016.07.109.
10. APEX4 v2021.10-0, Bruker AXS Inc., Madison, Wisconsin, USA, 2021.
11. Krause, L.; Herbst-Irmer, R.; Sheldrick, G. M.; Stalke, D., Comparison of silver and molybdenum microfocus X-ray sources for single-crystal structure determination. *J. Appl. Cryst.* **2015**, *48* (1), 3-10. DOI: 10.1107/S1600576714022985.
12. XPREP Ver. 2014/2, Bruker AXS Inc., Madison, Wisconsin, USA, 2014.
13. Sheldrick, G. M. SHELXT – Integrated space-group and crystalstructure determination. *Acta Crystallogr., Sect. A: Found. Adv.*, **2015**, *A 71*, 3–8. DOI: 10.1107/S2053273314026370.
14. Sheldrick, G. M. Crystal structure refinement with SHELXL. *Acta Crystallogr., Sect. C: Struct. Chem.*, **2015**, *C 71*, 3–8. DOI: 10.1107/S2053229614024218.
15. Dolomanov, O. V.; Bourhis, L. J.; Gildea, R. J.; Howard, J. A. K.; Puschmann, H. OLEX2: a Complete Structure Solution, Refinement and Analysis Program. *J. Appl. Cryst.* **2009**, *42*, 339-341. DOI: 10.1107/S0021889808042726.
16. Spek, A. L. PLATON SQUEEZE: A Tool for the Calculation of the Disordered Solvent Contribution to the Calculated Structure Factors. *Acta Crystallogr., Sect. C: Struct. Chem.*, **2015**,

C 71, 9–18. DOI: 10.1107/s2053229614024929.

17. Langmuir, I. THE DISSOCIATION OF HYDROGEN INTO ATOMS. III. THE MECHANISM OF THE REACTION. *J. Am. Chem. Soc.* **1916**, *38* (6), 1145–1156. DOI: 10.1021/ja02263a001.
18. Langmuir, I. THE ADSORPTION OF GASES ON PLANE SURFACES OF GLASS, MICA AND PLATINUM. *J. Am. Chem. Soc.* **1918**, *40* (9), 1361–1403. DOI: 10.1021/ja02242a004.
19. Langmuir, I. The Evaporation, Condensation and Reflection of Molecules and the Mechanism of Adsorption. *Phys. Rev.* **1916**, *8* (2), 149–176. DOI: 10.1103/physrev.8.149.
20. Fulmer, G. R.; Miller, A. J. M.; Sherden, N. H.; Gottlieb, H. E.; Nudelman, A.; Stoltz, B. M.; Bercaw, J. E.; Goldberg, K. I. NMR Chemical Shifts of Trace Impurities: Common Laboratory Solvents, Organics, and Gases in Deuterated Solvents Relevant to the Organometallic Chemist, *Organometallics*, **2010**, *29* (9), 2176-2179, DOI: 10.1021/om100106e.
21. Peng, Y.; Gong, T.; Cui, Y. A Homochiral Porous Metal–organic Framework for Enantioselective Adsorption of Mandelates and Photocyclizaton of Tropolone Ethers. *Chem. Commun.* **2013**, *49* (74), 8253. DOI: 10.1039/c3cc43549k.
